# A Web-Based, Provider-Driven Mobile App to Enhance Patient Care Coordination Between Dialysis Facilities and Hospitals: Development and Pilot Implementation Study

**DOI:** 10.2196/36052

**Published:** 2022-06-10

**Authors:** Laura C Plantinga, Courtney Hoge, Ann E Vandenberg, Kyle James, Tahsin Masud, Anjali Khakharia, Carol Gray, Bernard G Jaar, Janice P Lea, Christopher M O'Donnell, Richard Mutell

**Affiliations:** 1 Department of Medicine Emory University Atlanta, GA United States; 2 Emory University Hospital Midtown Emory Healthcare Atlanta, GA United States; 3 Department of Medicine Johns Hopkins School of Medicine Baltimore, MD United States; 4 Department of Epidemiology Johns Hopkins Bloomberg School of Public Health Baltimore, MD United States; 5 Nephrology Center of Maryland Baltimore, MD United States; 6 Apex Health Innovations Williamsburg, VA United States

**Keywords:** dialysis, hospitals, physicians, advanced practice providers, nurses, care coordination, mobile app

## Abstract

**Background:**

We piloted a web-based, provider-driven mobile app (*DialysisConnect*) to fill the communication and care coordination gap between hospitals and dialysis facilities.

**Objective:**

This study aimed to describe the development and pilot implementation of *DialysisConnect*.

**Methods:**

*DialysisConnect* was developed iteratively with focus group and user testing feedback and was made available to 120 potential users at 1 hospital (hospitalists, advanced practice providers [APPs], and care coordinators) and 4 affiliated dialysis facilities (nephrologists, APPs, nurses and nurse managers, social workers, and administrative personnel) before the start of the pilot (November 1, 2020, to May 31, 2021). Midpilot and end-of-pilot web-based surveys of potential users were also conducted. Descriptive statistics were used to describe system use patterns, ratings of multiple satisfaction items (1=not at all; 3=to a great extent), and provider-selected motivators of and barriers to using *DialysisConnect*.

**Results:**

The pilot version of *DialysisConnect* included clinical information that was automatically uploaded from dialysis facilities, forms for entering critical admission and discharge information, and a direct communication channel. Although physicians comprised most of the potential users of *DialysisConnect*, APPs and dialysis nurses were the most active users. Activities were unevenly distributed; for example, 1 hospital-based APP recorded most of the admissions (280/309, 90.6%) among patients treated at the pilot dialysis facilities. End-of-pilot ratings of *DialysisConnect* were generally higher for users versus nonusers (eg, “I can see the potential value of *DialysisConnect* for my work with dialysis patients”: mean 2.8, SD 0.4, vs mean 2.3, SD 0.6; *P*=.02). Providers most commonly selected reduced time and energy spent gathering information as a motivator (11/26, 42%) and a lack of time to use the system as a barrier (8/26, 31%) at the end of the pilot.

**Conclusions:**

This pilot study found that APPs and nurses were most likely to engage with the system. Survey participants generally viewed the system favorably while identifying substantial barriers to its use. These results inform how best to motivate providers to use this system and similar systems and inform future pragmatic research in care coordination among this and other populations.

## Introduction

### Background

The coordination of care is a significant challenge in the fragmented US health care system [1]. For US patients receiving dialysis, who are hospitalized for an average of 9.5 days per year (associated with annual Medicare costs of approximately US $12 billion) [2], transitions between the outpatient dialysis facility and the hospital present unique challenges. In addition to the usual elements of successful care transitions [3], dialysis-specific issues must be coordinated with providers during hospitalization and at discharge, such as identification and mitigation of difficulty with dialysis adherence; maintenance of patients’ dialysis schedules during hospitalization; and communication of changes in dry weight, vascular access status, or medications (particularly those that should be administered intravenously during outpatient dialysis treatments, such as antibiotics) to dialysis providers [4]. Although the frequency of dialysis treatment presents an opportunity to improve timely coordination for patients receiving dialysis [4], patients are likely to return to their facilities without having seen other outpatient providers who could help coordinate postdischarge care, and patients themselves may be unable to provide the reason for hospitalization or updated medical information after discharge. These care transition challenges contribute to poor outcomes, such as 30-day readmissions, which occur after approximately one-third of hospitalizations among patients receiving dialysis [2] and often before the patient presents to the dialysis facility [5]. Thus, early follow-up care after discharge is a necessary component of successful care transitions for these patients [6]: we and others have shown in previous work that time-sensitive clinical factors such as documented changes in dry weight [7], timely medication reconciliation [8], and increased physician encounters after discharge [9] are all associated with lower hospital readmission risk among patient receiving dialysis.

Electronic health records (EHRs) represent a possible means of instant information exchange between dialysis and hospital providers to address these issues; however, US outpatient dialysis facilities are usually managed independently and do not share EHRs with the hospitals to which their patients are admitted. Regional health care information exchanges are meant to circumvent these issues and close the patient information gap across health care settings [10]; however, the evidence for better outcomes is mixed [11], given that communication may not be timely, there may be no context for the information shared, and the information may not include what the provider is seeking (eg, information in medical notes rather than in the EHR). Thus, although the Centers for Medicare and Medicaid Services have explicitly prioritized the reduction of hospital readmissions in patients receiving dialysis [12-14]—and, implicitly, the improvement of care coordination between US hospitals and dialysis facilities—the lack of tools to ensure timely exchange of critical information during the hospital–dialysis facility transition remains a primary barrier to improving hospital outcomes in this population [15].

### Objective

To address this barrier, we conducted a pragmatic pilot study in which we developed *DialysisConnect*, a secure web-based provider communication platform, and implemented it at our 4 Emory Dialysis outpatient facilities and Emory University Hospital Midtown (EUHM). Emory Dialysis and EUHM share an academic affiliation but do not share health care management or, importantly, an EHR. We aimed to (1) gather information on the critical components of the system from stakeholders and potential users, including hospital providers (hospitalists, advanced practice providers [APPs; nurse practitioners or physician assistants], and social workers or care coordinators), and dialysis providers (nephrologists, APPs, nurses, and social workers); (2) develop and introduce the system into the clinical setting with the help of site champions to determine which potential users engaged with the system and how; and (3) examine provider perceptions of the system. Together, this information on the feasibility, acceptability, and potential sustainability of this system, as piloted, is important for future research addressing communication gaps and care coordination between settings for patients receiving dialysis. Here, we describe the development and pilot implementation of *DialysisConnect*.

## Methods

### Development of DialysisConnect

The initial wireframes for *DialysisConnect*, which were created by the technical team (led by RM) and showed each intended task (admission, communication, and discharge), were based on an existing platform for the exchange of transplant referral information for patients receiving dialysis [16]. The proposed workflow process for *DialysisConnect* was developed by the research team (including experts in epidemiology and health services research, hospital medicine, nephrology, gerontology, and engineering) [17]. Initial feedback on this *DialysisConnect* prototype (video simulation using static wireframes) was collected from 4 focus groups of stakeholders and potential users of *DialysisConnect* (excluding the site champions), as previously described [17]. The purpose of the focus groups (led by AEV) was to gather feedback on the desired elements and features of the system and increase future buy-in by the inclusion of potential users of *DialysisConnect* in its creation. Data regarding feedback on the proposed system were collated from the transcripts for the technical team (Multimedia Appendix 1). Incorporation of this initial feedback into the test system was prioritized by its importance to the focus group participants, the degree of automation that was possible, and the ease of building features into the system over a short period. Research team members thoroughly evaluated this test system and presented it to the clinical teams at the hospital and dialysis facilities in February 2020; disruptions to clinical care early in the COVID-19 pandemic led to an 8-month delay in the rollout, during which additional planned future upgrades to the system and user testing (n=9 potential users, including the *site champions* [JPL and TM at Emory Dialysis and CMO and KJ at EUHM]) were performed. Each participant was asked to perform a standardized set of tasks on the test system, with guidance from the research team (LCP and CH). Observations and feedback (Multimedia Appendix 2) were provided to the technical team for a final round of fixes and updates to the system before rollout.

**Figure 1:**
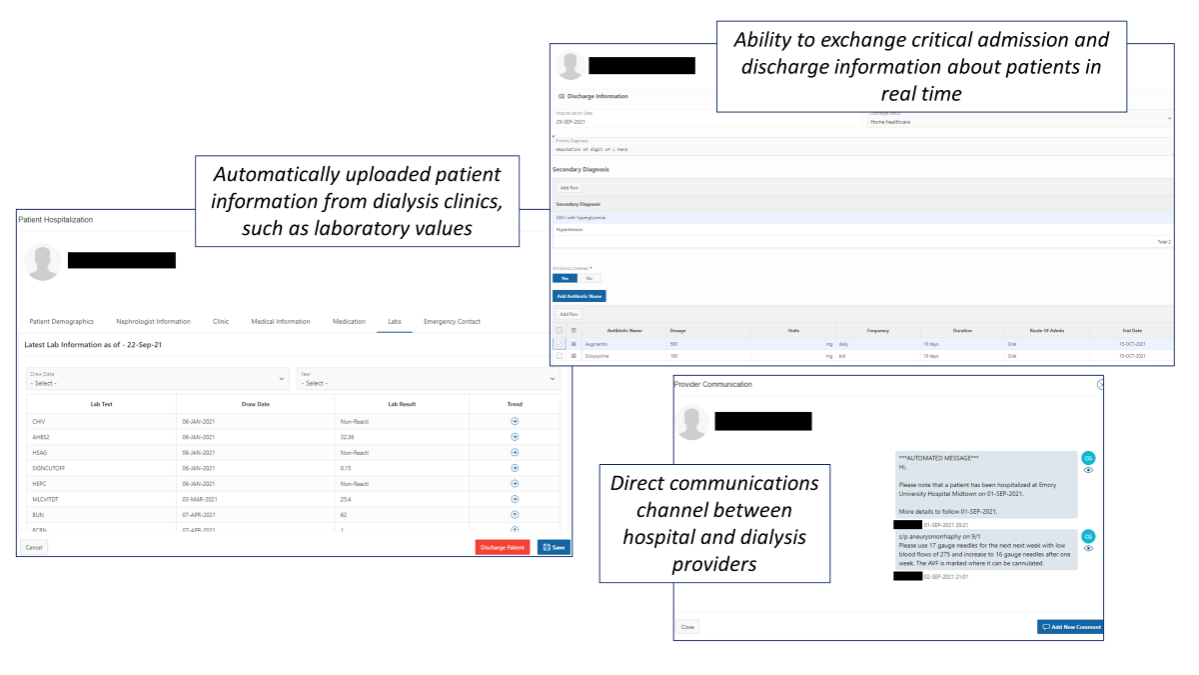
Overview of the major features of the DialysisConnect system.

Just before rollout, the team conducted remote group training sessions using the pilot version of *DialysisConnect* (Figure 1; Multimedia Appendix 3), and group emails were sent to all potential *DialysisConnect* users (as identified by champions) to announce the live rollout, along with detailed user guides (Multimedia Appendices 4 and 5) and contact information for questions. Throughout the pilot study, the list of potential users was updated as needed, and all significant upgrades (Textbox 1) were announced via email, including reminders about the availability of on-demand training sessions and revised user guides (Multimedia Appendices 4 and 5). The user guides included an idealized scenario of use for both hospital and dialysis providers, in which (1) a hospital provider identified an Emory Dialysis patient at admission; (2) the hospital provider reviewed any relevant, automatically uploaded information about the patient (eg, nephrologist, clinic, recent laboratories, and medications) and checked the reasons for admission; (3) a dialysis provider received an automated message about admission generated by the hospital provider’s entry and logged in to review the patient and status; (4) hospital and dialysis providers communicated via instant messages as needed or desired about the patient’s status throughout the hospitalization; (5) a hospital provider clicked through the discharge elements at the patient’s discharge; and (6) dialysis providers reviewed the discharge elements and, as needed, confirmed receipt (eg, for antibiotic orders). Given that this was a pilot in which we hoped to learn how staff engaged with the system, no specific expectations or benchmarks for the use of the system were communicated by the research team, although site champions were free to encourage use through whatever means they preferred. *DialysisConnect* remains available to all users; however, there has been no active encouragement of the use of *DialysisConnect* since May 2021.

**Substantial upgrades made to DialysisConnect during the pilot study.**

**Upgrades**

- November 19, 2020: automated file feed from Emory Dialysis updated to include nephrologist name and emergency contact information
- January 29, 2021: automated file feed from Emory Dialysis updated to include laboratory values and medications; option for users to view graphs of laboratory values over time added
- April 14, 2021: addition of “response required” option on messages (vs “read” status only), so that recipient was required to acknowledge the sender’s message (eg, to acknowledge antibiotic orders were received and acted upon)

### Pilot Implementation of DialysisConnect

#### Data Sources

Evaluation of the pilot implementation of *DialysisConnect* was primarily based on the system and provider survey data. System data downloaded directly from *DialysisConnect* included data on users, hospitalizations, page activities, and exchanged messages. Users were defined as potential users (all providers who had access to *DialysisConnect*), active users (logged in at least once during the pilot), and top users (defined by the ≥90th percentile of the number of log-ins or ≥45 log-ins). Daily user and activity data were aggregated by study week. Brief web-based surveys were sent to current potential users (regardless of actual use) at both Emory Dialysis and EUHM in January 2021 (n=106; midpilot) and in May 2021 (n=116; end of pilot); midpilot and end-of-pilot surveys included identical items related to actions and beliefs regarding *DialysisConnect*, adapted from implementation measures that were guided by the normalization process theory [18,19] and scored on a Likert scale (1=not at all; 2=to some extent; 3=to a great extent), as well as items assessing motivators of and barriers to using the system (Multimedia Appendix 6). Finally, EHR data from EUHM were used to identify hospitalizations that had occurred from November 1, 2020, to May 31, 2021, among Emory Dialysis patients.

#### Analysis

The system and user survey data were summarized using descriptive statistics. For survey data, the overall characteristics of participants and participant-identified motivators of and barriers to the use of *DialysisConnect* were described, and ratings of items related to actions and beliefs regarding *DialysisConnect* were described overall and stratified by user status and setting (dialysis facility vs hospital) at the time of the survey; paired 2-tailed *t* tests were used to compare midpilot and end-of-pilot survey ratings for the users who responded to both surveys. The capture of hospitalizations in *DialysisConnect* was estimated as the percentage of hospitalizations documented in the EHR among Emory Dialysis patients who were entered into *DialysisConnect* over the course of the pilot. All analyses were performed using Stata (version 17.0; StataCorp).

### Ethical Considerations

Provider participants in the initial focus groups and midpilot and end-of-pilot surveys provided informed consent and were incentivized with meals (focus groups) and nominal (US $10) gift cards (surveys). In this pragmatic study, potential users in the pilot were not incentivized to use the system. The need for consent was waived for the EHR and system data, which were only reported in aggregated form. The entire study protocol was approved by the Emory Institutional Review Board (IRB00102971).

## Results

### Features of DialysisConnect

An overview of the features of the pilot version of *DialysisConnect* is presented in Figure 1. The home page included an overall hospitalization report, listings of patients who were hospitalized at the time of the study and were previously hospitalized, and a message center (Multimedia Appendix 3). The admission feature allowed hospital providers to search for and select patients and review medical information about the patients and identify reasons for hospitalization; instant automated messages informed dialysis providers of admissions (Multimedia Appendix 3). The message feature allowed providers to view automated messages (at admission, discharge, and document upload), send and view user-initiated messages, check *read* status for messages, and request and upload documents (Multimedia Appendix 3). To prompt log-ins, users also received automated messages externally, by email (default) or SMS text messages, with links to the system. Hospital providers could use the discharge feature to complete a brief, instantaneously delivered discharge report, including discharge date, status, and diagnosis; antibiotics to order; medication changes; and dialysis prescription or dry weight changes (Multimedia Appendix 3). The system was flexible and allowed modifications during the pilot based on user requests or inputs (Textbox 1).

### DialysisConnect Users and Activity

#### Characteristics of Users

A total of 120 individuals at EUHM and Emory Dialysis (identified by project champions) had access to *DialysisConnect* over the course of the pilot (n=61, 50.8% from EUHM and n=59, 49.2% from Emory Dialysis; Figure 1). Potential hospital users were primarily hospitalists but also included APPs, nephrology fellows, and care coordinators and social workers. Most potential dialysis facility users were nurses or nurse managers, followed by nephrologists, APPs, vascular access team members, and a dietitian (Table 1).

**Table 1:** Cumulative *DialysisConnect* user activity and message activity during the pilot (November 1, 2020 to May 31, 2021)—overall and by role.

User role		Potential users (n=120), n (%)	Active users^a^ (n=46), n (%)	Top users^b^ (n=11), n (%)	Total log-ins (n=1699), n (%)	Total messages^c^ (n=1145), n (%)	User-initiated messages only (n=573), n (%)
**Hospital**							
	All hospital users	61 (100)	16 (100)	1 (100)	300 (100)	913 (100)	348 (100)
	Hospitalist	49 (80.3)	9 (56.3)	0 (0)	21 (7)	7 (0.8)	5 (1.4)
	APP^d^	4 (6.6)	3 (18.8)	1 (100)	238 (79.3)	861 (94.3)	339 (97.4)
	Nephrology fellow	5 (8.2)	4 (25)	0 (0)	41 (13.7)	45 (4.9)	4 (1.1)
	Care coordinator or social worker	3 (4.9)	0 (0)	0 (0)	0 (0)	0 (0)	0 (0)
**Dialysis facility**							
	All dialysis facility users	59 (100)	30 (100)	10 (100)	1399 (100)	232 (100)	225 (100)
	Nephrologist	12 (20.3)	7 (23.3)	0 (0)	54 (3.9)	7 (3)	7 (3.1)
	APP	2 (3.4)	2 (6.7)	2 (20)	277 (19.8)	202 (87.1)	202 (89.8)
	Dialysis nurse or nurse manager	26 (44.1)	21 (70)	8 (80)	1068 (76.3)	23 (9.9)	16 (7.1)
	Dietitian	1 (1.7)	0 (0)	0 (0)	0 (0)	0 (0)	0 (0)
	Vascular access team	2 (3.4)	0 (0)	0 (0)	0 (0)	0 (0)	0 (0)
	Administrative assistant	7 (11.9)	0 (0)	0 (0)	0 (0)	0 (0)	0 (0)
	Social worker	9 (15.3)	0 (0)	0 (0)	0 (0)	0 (0)	0 (0)

^a^Logged into the system at least once over the course of the pilot.

^b^Top users were in the ≥90th percentile for all potential users (≥45 log-ins).

^c^Includes automated messages sent by the system at admission, at discharge, and when documents were uploaded.

^d^APP: advanced practice provider.

#### DialysisConnect Log-Ins

**Figure 2:**
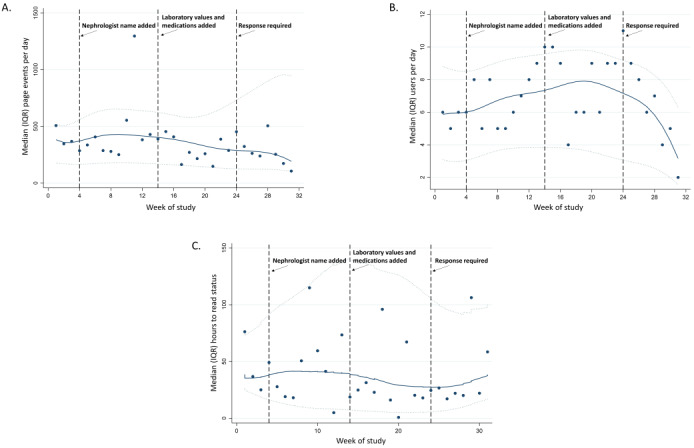
The (A) median number of DialysisConnect page events (number of pages with which users interacted; eg, home page and admission page), (B) number of DialysisConnect users, and (C) the time (hours) from when the message was sent to when the message was read per week over the period from November 1, 2020, to May 31, 2021.

From November 1, 2020, to May 31, 2021, of the 120 potential users included in the analysis, 46 (38.3%) were active users and 11 (9.1%) were top users (Table 1). Hospital APPs (238/300, 79.3% log-ins; 217/238, 91.2% by a single APP [CG]), dialysis facility APPs (277/1399, 19.80% log-ins), and dialysis facility nurses and nurse managers (1068/1399, 76.34% log-ins) were responsible for most of the system log-ins (Table 1). Physicians were responsible for 4.41% (75/1699) of log-ins; care coordinators (hospital) and the dietitian, vascular access team members, administrative assistants, and social workers (dialysis facility) never logged in to *DialysisConnect* (Table 1). Over the course of the pilot, activity, as measured by the number of page events (number of pages with which users interacted, eg, home page and admission page) and unique users logging in (Figure 2) per week, was relatively stable, with no evidence of increase at the time of substantial upgrades (Textbox 1), and there was a decline in both measures at the end of the pilot.

#### DialysisConnect Messages

Table 1 shows the total message activity in *DialysisConnect* during the pilot. A total of 1145 messages were sent, 573 (50.04%) of which were user-initiated messages (541/573, 94.4% by a single hospital APP [CG] and dialysis facility APPs; Table 1). Over the course of the pilot, the median number of hours to *read* status for messages sent varied but was <48 hours in all weeks of the pilot (Figure 2). All 21 messages that were sent with the *response required* option (April 14, 2021, to May 31, 2021) received a response, with a median response time of 18.3 (IQR 2.3-70.9) hours.

#### Hospitalization Events Entered Into DialysisConnect

A total of 309 incident hospitalization events, representing 184 unique patients, were entered into *DialysisConnect* during the study period by a single hospital APP (CG; 280/309, 90.6%) and 2 nephrology fellows (29/309, 9.4%). Most events (276/309, 89.3%) were among patients receiving hemodialysis, and the remaining events were among patients treated with peritoneal dialysis (31/309, 10%) or an unspecified modality (2/309, 0.6%). Of the 309 hospital events, 296 (95.8%; 178/184, 96.7% of individuals) were among active Emory Dialysis patients whose events occurred in the period from November 1, 2020, to May 31, 2021, and were included in the EUHM EHR (n=296, 81.3% of all 364 hospital events recorded for Emory Dialysis patients in the EUHM EHR in the same period). The capture of events was higher for inpatient admissions (223/260, 85.8%) than for observational stays (73/104, 70.2%). After 11 months from the end of the pilot (April 30, 2022), 335 additional hospitalization events were entered into the system.

### User Perceptions of DialysisConnect

#### Characteristics of Survey Participants

Midpilot and end-of-pilot surveys were completed by 21.7% (23/106) and 22.4% (26/116) of potential users; 23% (9/40) of respondents completed both surveys. Participants were primarily physicians, APPs, and nurses who had been working in their setting for >12 months. Most were actual users at the time of the survey, although this dropped from 68% (15/22) to 54% (14/26) by the end of the pilot (Table 2).

**Table 2:** Characteristics of *DialysisConnect* user survey participants (N=40^a^).

Characteristic			Survey administered, n (%)		
			Midpilot (n=23)		End of pilot (n=26)
**Site^b^**					
	Dialysis facility	10 (43)		15 (58)	
	Hospital	13 (57)		11 (42)	
**Role**					
	Physician	11 (48)		10 (39)	
	APP^c^	5 (22)		4 (15)	
	Nurse	3 (13)		7 (27)	
	Social worker	1 (4)		2 (8)	
	Other	3 (13)		3 (12)	
**Length of time in role (months)**					
	<6	3 (13)		1 (4)	
	6-12	3 (13)		0 (0)	
	>12	17 (74)		25 (96)	
**User of the system at time of survey**					
	Yes	15 (68)		14 (54)	
	No	7 (32)		12 (46)	

^a^Nine participants filled out both surveys, leaving 40 unique individuals across both surveys.

^b^Nephrologists included in the dialysis facility group.

^c^APP: advanced practice provider.

#### Participant Ratings of DialysisConnect

Overall, the mean ratings of the items representing actions and beliefs about *DialysisConnect* were positive (Table 3). For both the midpilot and end-of-pilot surveys, users rated items more positively than nonusers; for example, end-of-pilot ratings were 2.8 versus 2.3 (*P*=.02) for “I can see the potential value of DialysisConnect for my work with dialysis patients” and 2.4 versus 1.8 (*P*=.04) for “Sufficient training is provided to enable staff to implement DialysisConnect” (Table 3). In general, participants working in dialysis facilities provided more positive ratings than those working in the hospital, although they rated the system as more disruptive to working relationships (end-of-pilot ratings, 2.5 vs 1.9; *P*=.03); only a few of the differences between participants in the 2 settings were statistically significant (Table 4). For the participants who completed both surveys (9/40, 23%), there were no differences between the midpilot and end-of-pilot survey ratings, except for the management support of *DialysisConnect*, which, on average, was rated more positively at midpilot than at the end of pilot (2.9 vs 2.3), although this difference was not statistically significant (*P*=.10).

**Table 3:** Ratings of *DialysisConnect* by survey participants in midpilot (1/21) and end-of-pilot (5/21) surveys—overall and by user status—at the time of the survey.

Item	Rating (1=not at all; 2=to some extent; 3=to a great extent)							
	Midpilot survey				End-of-pilot survey			
	Overall^a^, mean (SD)	Users^a^, mean (SD)	Nonusers^a^, mean (SD)	*P* value^b^	Overall, mean (SD)	Users^a^, mean (SD)	Nonusers^a^, mean (SD)	*P* value^b^
I can see how *DialysisConnect* differs from usual ways of communicating with dialysis facilities or hospitals	2.2 (0.5)	2.3 (0.6)	2.0 (0.0)	.17	2.5 (0.6)	2.6 (0.5)	2.3 (0.7)	.18
I understand how *DialysisConnect* could improve transitions of care for dialysis patients	2.3 (0.5)	2.4 (0.5)	2.1 (0.4)	.25	2.6 (0.6)	2.8 (0.4)	2.3 (0.7)	.04
I can see the potential value of *DialysisConnect* for my work with dialysis patients	2.4 (0.6)	2.5 (0.6)	2.1 (0.4)	.23	2.5 (0.6)	2.8 (0.4)	2.3 (0.6)	.02
Users of the system in this organization have a shared understanding of the purpose of *DialysisConnect*	2.4 (0.5)	2.5 (0.5)	2.2 (0.4)	.14	2.3 (0.6)	2.3 (0.7)	2.3 (0.5)	.76
There are key people at my institution who drive *DialysisConnect* forward and get others involved	2.5 (0.6)	2.6 (0.5)	2.2 (0.8)	.11	2.3 (0.6)	2.4 (0.8)	2.1 (0.4)	.30
I believe that participating in *DialysisConnect* is a legitimate part of my role in caring for dialysis patients	2.3 (0.7)	2.4 (0.7)	2.0 (0.6)	.22	2.5 (0.6)	2.7 (0.6)	2.2 (0.6)	.03
I am open to working with colleagues to optimize our use of *DialysisConnect* for patient care	2.6 (0.5)	2.7 (0.5)	2.3 (0.5)	.05	2.5 (0.6)	2.6 (0.6)	2.3 (0.5)	.18
I support *DialysisConnect*	2.5 (0.5)	2.7 (0.5)	2.1 (0.4)	.02	2.6 (0.5)	2.8 (0.4)	2.3 (0.5)	.02
I can easily integrate *DialysisConnect* into my existing work	2.1 (0.7)	2.3 (0.7)	1.7 (0.5)	.08	2.3 (0.8)	2.5 (0.9)	2.2 (0.7)	.30
*DialysisConnect* disrupts working relationships^c^	1.3 (0.7)	1.4 (0.8)	1.2 (0.4)	.48	1.3 (0.6)	1.2 (0.6)	1.5 (0.5)	.27
I have confidence in other people’s ability to use *DialysisConnect*	2.2 (0.5)	2.3 (0.5)	2.0 (0.6)	.25	2.2 (0.7)	2.3 (0.7)	2.2 (0.6)	.66
Work is assigned to those with skills appropriate to *DialysisConnect*	2.2 (0.7)	2.3 (0.8)	2.0 (0.0)	.57	2.1 (0.6)	2.1 (0.8)	2.0 (0.0)	.80
Sufficient training is provided to enable staff to implement *DialysisConnect*	2.2 (0.4)	2.3 (0.5)	2.0 (0.0)	.25	2.1 (0.7)	2.4 (0.7)	1.8 (0.5)	.04
Sufficient resources are available to support *DialysisConnect*	2.3 (0.5)	2.4 (0.5)	2.0 (0.0)	.16	2.4 (0.6)	2.5 (0.7)	2.1 (0.4)	.21
Management adequately supports *DialysisConnect*	2.5 (0.5)	2.6 (0.5)	2.0 (0.0)	.03	2.3 (0.7)	2.5 (0.7)	2.0 (0.5)	.10
I value the effects that *DialysisConnect* has had on my work	2.2 (0.9)	2.4 (0.8)	1.5 (0.6)	.08	2.4 (0.7)	2.5 (0.7)	2.0 (0.6)	.11
The staff here agree that *DialysisConnect* is worthwhile	2.3 (0.6)	2.4 (0.5)	2.0 (0.7)	.19	2.3 (0.5)	2.5 (0.5)	2.0 (0.0)	.06
Feedback about *DialysisConnect* can be used to improve it in the future	2.5 (0.5)	2.7 (0.5)	2.3 (0.5)	.10	2.5 (0.5)	2.6 (0.5)	2.4 (0.5)	.27
I can easily modify how I work with *DialysisConnect*	2.1 (0.7)	2.3 (0.7)	1.7 (0.5)	.05	2.2 (0.7)	2.4 (0.8)	2.0 (0.4)	.20

^a^N=22 (n=15, 68% users and n=7, 32% nonusers) for the midpilot survey; N=26 (n=14, 54% users and n=12, 46% nonusers) for the end-of-pilot survey. One of the respondents did not answer the question regarding system use.

^b^Users versus nonusers at the time of the survey by *t* test.

^c^Ratings were flipped for this item (1=not at all disruptive; 3=disruptive to a great extent).

**Table 4:** Ratings of *DialysisConnect* by survey participants in midpilot (1/21) and end-of-pilot (5/21) surveys—overall and by participant setting.

Item	Rating (1=not at all; 2=to some extent; 3=to a great extent)								
	Midpilot survey					End-of-pilot survey			
	Overall^a^, mean (SD)	Hospital^a^, mean (SD)	Dialysis facility^a^, mean (SD)	*P* value^b^	Overall^a^, mean (SD)		Hospital^a^, mean (SD)	Dialysis facility^a^, mean (SD)	*P* value^b^
I can see how *DialysisConnect* differs from usual ways of communicating with dialysis facilities or hospitals	2.2 (0.5)	2.2 (0.4)	2.3 (0.7)	.52	2.5 (0.6)		2.3 (0.6)	2.7 (0.5)	.09
I understand how *DialysisConnect* could improve transitions of care for dialysis patients	2.3 (0.5)	2.2 (0.4)	2.5 (0.5)	.20	2.6 (0.6)		2.3 (0.6)	2.8 (0.4)	.02
I can see the potential value of *DialysisConnect* for my work with dialysis patients	2.4 (0.6)	2.2 (0.6)	2.6 (0.5)	.14	2.5 (0.6)		2.2 (0.6)	2.8 (0.4)	.005
Users of the system in this organization have a shared understanding of the purpose of *DialysisConnect*	2.5 (0.5)	2.4 (0.5)	2.6 (0.5)	.42	2.3 (0.6)		2.0 (0.5)	2.5 (0.5)	.05
There are key people at my institution who drive *DialysisConnect* forward and get others involved	2.5 (0.6)	2.5 (0.5)	2.6 (0.7)	.84	2.3 (0.6)		2.2 (0.7)	2.4 (0.7)	.58
I believe that participating in *DialysisConnect* is a legitimate part of my role in caring for dialysis patients	2.3 (0.7)	2.2 (0.7)	2.5 (0.7)	.25	2.5 (0.6)		2.1 (0.7)	2.7 (0.5)	.01
I am open to working with colleagues to optimize our use of *DialysisConnect* for patient care	2.6 (0.5)	2.5 (0.5)	2.7 (0.5)	.45	2.5 (0.6)		2.3 (0.6)	2.7 (0.5)	.09
I support *DialysisConnect*	2.5 (0.5)	2.5 (0.5)	2.5 (0.5)	.86	2.6 (0.5)		2.4 (0.5)	2.7 (0.5)	.06
I can easily integrate *DialysisConnect* into my existing work	2.1 (0.7)	2.0 (0.7)	2.2 (0.6)	.49	2.3 (0.8)		1.9 (0.8)	2.7 (0.6)	.01
*DialysisConnect* disrupts working relationships^c^	1.3 (0.7)	1.2 (0.6)	1.5 (0.8)	.32	1.3 (0.6)		1.3 (0.5)	1.3 (0.6)	.92
I have confidence in other people’s ability to use *DialysisConnect*	2.2 (0.5)	2.1 (0.5)	2.4 (0.5)	.14	2.2 (0.7)		1.9 (0.5)	2.5 (0.6)	.03
Work is assigned to those with skills appropriate to *DialysisConnect*	2.2 (0.7)	2.1 (0.8)	2.3 (0.5)	.55	2.1 (0.6)		1.9 (0.6)	2.2 (0.6)	.30
Sufficient training is provided to enable staff to implement *DialysisConnect*	2.3 (0.5)	2.3 (0.5)	2.3 (0.5)	.87	2.1 (0.7)		2.1 (0.6)	2.1 (0.7)	.91
Sufficient resources are available to support *DialysisConnect*	2.3 (0.5)	2.3 (0.5)	2.4 (0.5)	.76	2.4 (0.6)		2.3 (0.5)	2.4 (0.7)	.81
Management adequately supports *DialysisConnect*	2.5 (0.5)	2.3 (0.5)	2.7 (0.5)	.18	2.3 (0.7)		2.0 (0.8)	2.5 (0.5)	.10
I value the effects that *DialysisConnect* has had on my work	2.2 (0.8)	2.0 (0.9)	2.4 (0.7)	.35	2.4 (0.7)		2.1 (0.8)	2.5 (0.5)	.19
The staff here agree that *DialysisConnect* is worthwhile	2.3 (0.6)	2.3 (0.6)	2.3 (0.5)	.78	2.3 (0.5)		2.3 (0.5)	2.4 (0.5)	.74
Feedback about *DialysisConnect* can be used to improve it in the future	2.6 (0.5)	2.6 (0.5)	2.5 (0.5)	.60	2.5 (0.5)		2.4 (0.5)	2.7 (0.5)	.14
I can easily modify how I work with *DialysisConnect*	2.2 (0.7)	2.1 (0.8)	2.3 (0.7)	.47	2.2 (0.7)		2.0 (0.8)	2.3 (0.6)	.23

^a^N=23 (n=13, 57% hospital and n=10, 43% dialysis facilities) for the midpilot survey; N=26 (n=11, 42% users and n=15, 58% nonusers) for the end-of-pilot survey.

^b^Participants from the hospital versus dialysis facilities at the time of the survey by *t* test.

^c^Ratings were flipped for this item (1=not at all disruptive; 3=disruptive to a great extent).

#### User-Identified Motivators of and Barriers to Using DialysisConnect

**Figure 3:**
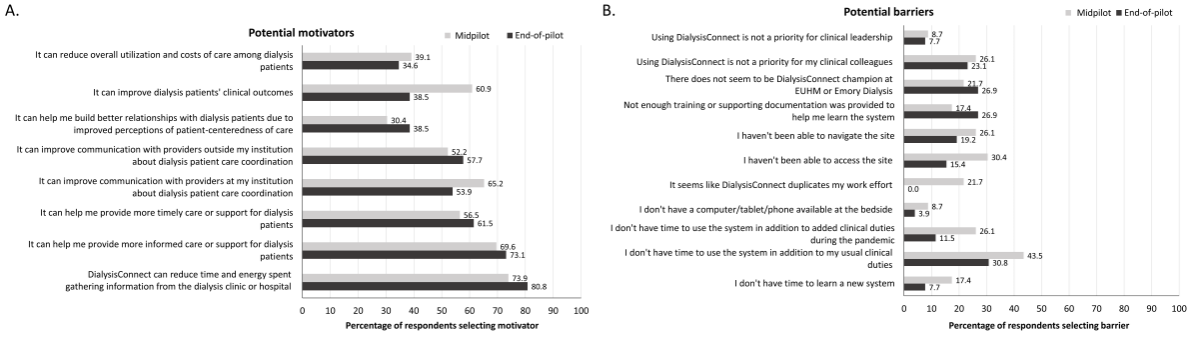
(A) Motivators of and (B) barriers to the use of DialysisConnect identified by user survey participants in the user survey. Participants could select >1 motivator and barrier; hence, total percentages exceed 100%. EUHM: Emory University Hospital Midtown.

Overall, among the lists of potential motivators and barriers, motivators (Figure 3) were more commonly selected by the survey participants than barriers (Figure 3). The most common motivators selected were the reduction in time spent gathering information, more informed care, and more timely care and support (21/26, 81%; 19/26, 73%; and 16/26, 62%, respectively, at the end of the pilot; Figure 3). The most common barriers selected were lack of time in addition to usual duties, lack of site champions, and insufficient training or documentation (8/26, 31%; 7/26, 27%; and 7/26, 27%, respectively, at the end of the pilot; Figure 3). When asked to rank the top motivator, participants most commonly selected the potential to gather information, the ability to provide more informed care, and the potential to improve communication with outside providers (midpilot: 6/23, 26%; 6/23, 26%; and 4/23, 17%, respectively; end of pilot: 11/26, 42%; 4/26, 15%; and 4/26, 15%, respectively). At midpilot, the top-ranked barriers were lack of time in addition to usual duties (4/23, 17%) and insufficient training or documentation, inability to navigate the site, and perceived duplication of work (3/23, 13% each); at the end of the pilot, lack of time, in addition to usual duties (8/26, 31%) and inability to navigate the site (5/26, 19%), were the most common top-ranked barriers, and no participants named perceived duplication of work as a top barrier to the use of *DialysisConnect*.

#### Other Participant Feedback

By the end of the pilot, 86% (12/14) of those using *DialysisConnect* reported intending to keep using the system, and 92% (11/12) of those who did not use *DialysisConnect* reported intending to start. When asked for suggestions for improvement, participants suggested features that were not included (eg, including patients admitted to non-Emory hospitals and integration of *DialysisConnect* into the EHR); however, they also listed multiple features and support that were provided either throughout the pilot (eg, web-based training) or during the pilot in response to real-time feedback (eg, updating laboratories and medications and sending notifications to physicians only about their patients). In free-text responses, participants identified issues with suboptimal use (“...system functions well if staff will use it”) and internal communication about the system (“...don’t recall any of the clinical admin or patient care staff mentioning DialysisConnect...”) but also praised the system’s utility (“I believe it to be excellent source to improve communication and care for our patients” and “It has been and continues to be a great help in caring for the in-center dialysis patients...”) and ease of use (“...system is user-friendly and even on the busiest days doesn’t take provider more than a few minutes to add or update a patient...”).

## Discussion

### Principal Findings

In this study, we developed and piloted a flexible web-based communication portal between providers at dialysis facilities and hospitals, the design and content of which were driven by potential users in both settings, suggesting that *DialysisConnect* is a potentially feasible solution to the gap in communication between dialysis facilities and hospitals. Although most of the potential users with access to the system were physicians, APPs in both settings and dialysis nurses were the most active users during the pilot. Our data also suggest that *DialysisConnect* is a usable solution. In midpilot and end-of-pilot surveys, both users and nonusers of the system rated *DialysisConnect* positively, and users also stated that the system was easy to use and did not disrupt their workflow. Survey participants identified multiple motivators for using *DialysisConnect*, predominantly the potential of the system to reduce time and energy spent in gathering information from other settings. However, future work in this area would need to address several reported barriers to the use of *DialysisConnect*, including a lack of time in addition to usual clinical duties, perceived lack of a system champion, and insufficient training and documentation.

Provider perceptions of lack of time in addition to usual clinical duties was an expected barrier in this pilot, given the new, untested nature of the system and the general challenges of affecting provider behavior change. Including multiple, active, and contextually relevant behavior change strategies, such as continuing education incentives, might help overcome this barrier [20,21]. In fact, we found that the barrier of perceived duplicated work effort was no longer identified as a barrier by participants at the end of the pilot study, suggesting that users may have dropped processes made obsolete by *DialysisConnect* (eg, phone calls).

Providers also commonly reported that a lack of training and supporting documentation was a barrier to using *DialysisConnect*. However, our research team provided multiple group and individual training sessions and detailed user guides for potential users in both settings at the start of the pilot, as well as additional, as-needed group or individual training sessions and updated user guides with every *DialysisConnect* user communication. The perception of lack of training and documentation despite these efforts may have been partially because of COVID-19–related disruptions; the research team was unable to provide formal and informal in-person training, as originally planned, and many potential users may not have had web-based meetings and email communications in their usual workflow and, thus, may have missed training opportunities. Importantly, the perception of lack of training is also likely partially because of another commonly reported barrier: the reported lack of support by management and key people driving *DialysisConnect* forward. This lack of support may have driven poor attendance in the web-based group training sessions.

In addition to the abovementioned barriers, several potential users noted in the surveys that the lack of integration, or even an icon to enter *DialysisConnect* from the desktop rather than navigating to the website, was a barrier. The lack of EHR integration also meant that automated information on patients had to be uploaded via patient census files that were provided by the dialysis facilities (but not hospitals), causing some lags in the information, particularly for new patients. The feasibility of including this information for other dialysis facilities or hospitals in future implementation would depend on the willingness of health care administration and information technology to provide such files or facilitate EHR integration of the system. However, the lack of EHR integration provided some advantages as well, as the Health Insurance Portability and Accountability Act–compliant system was web-based and, therefore, EHR-agnostic. It was implementable in both settings with fewer institution-specific, often burdensome information technology compliance requirements than EHR-integrated systems. Thus, we were able to offer the system to potential users across both settings and make changes to the system in real time.

Despite these barriers, 81.3% (296/364) of hospital admissions were captured over the course of the pilot study. Furthermore, this capture rate was accomplished by a single APP in the hospital setting and suggests that fidelity to the intervention can be high, even with few users: overall, a small number of users, who were primarily APPs and nurses, were responsible for most *DialysisConnect* activities. APPs were far more active than physicians at either site, which may reflect the APPs’ greater time available for clinical care, site champion encouragement of APPs specifically, and the existing roles of APPs in institutions as independent physician extenders. In fact, our APPs were already performing most of the intersetting communication at the start of the pilot. Although social workers expressed initial enthusiasm for *DialysisConnect* in early focus groups [17], none of the social workers, who were given full access to the system and who facilitated critical nonclinical support (eg, transportation and financial assistance) to ensure a successful care transition, logged into the system during the pilot. This may reflect a lack of site champion encouragement of social workers; the perception of *DialysisConnect* as a platform to exchange medical information only; or particular disengagement of social workers, given pandemic-related increased workloads (and, for dialysis social workers, adjustments because of working from home during our pilot). Other essential care transition roles, such as care coordinators and vascular access team members, were also not represented among the users.

Finally, there is evidence that *DialysisConnect* could be a sustainable intervention. Most survey participants who were users of *DialysisConnect* stated that they would continue using the system at the end of the pilot. Furthermore, we found that many hospitalization events continued to be entered into *DialysisConnect* after the conclusion of the pilot when no communication encouraging its use was being sent by the research team. Site champions who identify and assign users at defined points in the workflow are likely to increase their sustainability. Sustainability is a key attribute for interventions such as *DialysisConnect*, which would be used in pragmatic settings where research teams are usually not embedded.

### Comparison With Prior Work

The reporting of interventions to reduce readmissions, specifically in the population of patients receiving dialysis, is limited to a few quality improvement projects and is generally focused on effectiveness rather than implementation. For example, the study by Wingard et al [22] reported the results of a pre-post study of the effectiveness of a phased, multicomponent intervention aimed at reducing hospital readmissions among patients at 26 US dialysis facilities (with patients from 18 nonrandomized facilities serving as controls). Readmissions were reduced after the introduction of the intervention (0.88-0.66 per patient-year) in the intervention facilities; however, the decline was not statistically significant compared with the decline in the control facilities (*P*=.26). Sparse information about implementation was provided, except that they found that only 42% of the patients were successfully contacted by assigned case managers within 30 days of discharge for the posthospitalization call or visit intervention component. Similarly, in another pre-post quality improvement study with a single-component intervention (postdischarge telephone follow-up) aimed at reducing readmissions in a dialysis population admitted to a single hospital, readmissions were reduced (from 28.4% to 24.6% in the 3-month project). However, in an audit, investigators found that only 71% of visits were assigned to a manager; of these, only 80% recorded a patient call attempt, and of these, 38% of patients completed the interview [23]. Although our intervention did not include a direct patient contact component, these prior results highlight the challenges for staff, particularly nurses, in dedicating time to such tasks, even when the tasks are supported by health care administration and assigned as part of the clinical workflow. This difficulty is exacerbated by inadequate dialysis nurse staffing levels, which have been shown to be predictive of hospital readmission [24,25].

### Limitations

Limitations other than those noted previously deserve mention. First, the design of *DialysisConnect* precluded the inclusion of nephrologists who attended Emory Dialysis and EUHM as users in both settings; thus, they were only able to log in as dialysis facility users. Second, the user surveys had low response rates, which may have biased our results in either direction; however, we were able to collect data from users versus nonusers and from both settings. Third, some suggested changes to the system may have increased its use but were very labor intensive for our initial, limited pilot, including adding the ability for dialysis providers to *start* a hospitalization event when they send the patient to the hospital and incorporate information from the emergency department. Fourth, *DialysisConnect* was developed without patient or surrogate input and was unlikely to address the sociodemographic factors associated with hospital readmission in previous studies [26,27]. Fifth, system use logs are temporary and potentially helpful information, such as pages visited more frequently, SMS text messages versus email preferences for alerts, and access via mobile versus web browsers, which cannot be determined; for future studies, these and similar systems could be modified to create permanent logs of system use to better inform implementation. Sixth, the number of users trained was difficult to track in the virtual environment, and it was even more difficult to track the level of engagement with training; future studies could include brief surveys about the level of confidence in using the system to inform training efforts. Finally, the COVID-19 pandemic both delayed and shortened our pilot and altered our approach to rollout and training. Although few providers named lack of time because of additional duties related to COVID-19 in our surveys (none by the end of the pilot), it is likely that the pandemic also had some impact on provider behavior and willingness to use *DialysisConnect*, especially given that a surge of patients receiving dialysis and hospitalized at EUHM for COVID-19 and related complications occurred during our pilot (January 2021 to February 2021).

### Lessons Learned for Future Studies

Future work using *DialysisConnect* or similar systems could leverage the most commonly reported motivator: the potential to save time over current processes. Testimonials from current users, who were generally satisfied and reported that *DialysisConnect* not only provided informed care but also saved time, may be helpful in convincing reluctant users to invest initial time to learn the system and incorporate it into work processes. These users, who are peers rather than members of the research team and are current and frequent users of the system, would be ideal system champions and essential for successful implementation. When targeting potential users and champions, investigators should consider that APPs (if available) may be more likely to engage with the system than physicians. Dialysis nurses, who are critical to the postdischarge processes, could be encouraged and potentially incentivized to learn and use the system. In addition, social workers should be engaged directly to ensure that they understand their essential role in care transitions, as well as the ability of the system to inform timely recognition of the need for services. The addition of content highly relevant to social workers (such as transportation needs for follow-up appointments or requests for referrals for mental health services) might also improve engagement. As much as possible, involving hospital or dialysis facility administration to encourage providers to use the system and facilitate the workflow changes required could increase provider engagement. EHR integration, if possible, is ideal; however, the inclusion of automated file feeds where available, the use of checkboxes and drop-down lists to limit typing, and the inclusion of only the most critical elements for admission and discharge considerably minimizes the time spent in the system, which is a message that can be prioritized by site champions.

### Conclusions

*DialysisConnect* is a flexible and adaptable intervention for enhancing care coordination between dialysis facilities and hospitals that demonstrates the feasibility, usability, and sustainability. In addition, this pilot implementation study informs strategies to overcome multiple potential barriers to the use of *DialysisConnect* and similar interventions. Future work in this area should consider appropriate system champions, identification of key users and tasks to be incorporated into the workflow, and site-specific methods to encourage provider behavior changes.

## Appendix

Multimedia Appendix 1 Focus group feedback.

Multimedia Appendix 2 User testing feedback.

Multimedia Appendix 3 Additional screenshots.

Multimedia Appendix 4 Hospital user guide.

Multimedia Appendix 5 Dialysis user guide.

Multimedia Appendix 6 Provider survey.

## Abbreviations

APP: advanced practice provider
EHR: electronic health record
EUHM: Emory University Hospital Midtown
