# Supplementary material for: A Web-Based, Provider-Driven Mobile App to Enhance Patient Care Coordination Between Dialysis Facilities and Hospitals: Development and Pilot Implementation Study
Source: JMIR Form Res. 2022 Jun 10;6(6):e36052. doi: 10.2196/36052 (PMC9233252; doi:10.2196/36052)
Supplement: Multimedia Appendix 1 [file formative_v6i6e36052_app1.docx]

**Initial focus group feedback: DialysisConnect**

Initial feedback on the DialysisConnect prototype was collected in four focus groups of stakeholders and potential users of DialysisConnect in March 2019. We purposively recruited participants from four groups of potential users: hospitalists affiliated with Emory University Hospital Midtown (EUHM), EUHM staff, nephrologists affiliated with Emory Dialysis, and Emory Dialysis staff. The purpose of the focus groups (led by A.E.V.) was to gather feedback on the desired elements and features of the system and increase future buy-in, by including potential users of DialysisConnect in its creation. Data regarding feedback on the proposed system were collated from transcripts for the technical team (below). Incorporation of this initial feedback by the technical team was prioritized by its importance to the focus group participants (=number of unique mentions), degree of automation that was possible, and the ease of building features into the system over a short period. Using these data, the technical team built the test system.

| **Category** | **Feedback** |
| --- | --- |
| Patient information | -Dialysis and hospital groups requested: name, DOB, address, phone, insurance information, emergency contact  -Multiple participants in hospital groups preferred SSN to be masked or removed |
| Hospitalization reason | -Hospitalists requested ability for dialysis facilities to enter information regarding reason for hospitalization pre-emptively  -Dialysis staff desired information that patient was in hospital as well as reason  -Common reasons mentioned across groups:   - Fluid overload/shortness of breath/suspected heart failure - De-clotting the line/ clotted graft/ access issue/ access placement - Suspected line-related bacteremia - Low blood pressure/ hypotensive episode/syncopal episode - Hyperkalemia - Chest pain |
| Documents | -Most requested a standard set of documents for most hospitalization reasons or list of documents to be checked as needed  -Hospital groups desired:   - Dialysis prescription/orders - Flow sheets - Medication lists   -Hospitalists suggested status icons to avoid re-requests |
| Provider information | -All groups agreed dialysis facility and nephrologist names and contact information should be included |
| Medical information | -Suggestions across groups:   - Dry weight goals - Type of access - Problem lists/comorbid conditions - DNR status (dialysis staff only) |
| Discharge information | -Suggestions across groups:   - Medication changes, especially antibiotic orders - Follow-up appointments and referrals such as for mental health - Changes in the dialysis prescription - Last hospital note - Discharge status including AMA - Hospital labs - H&P |

AMA, against medical advice; DNR, do not resuscitate; DOB, date of birth; H&P, history and physical; SSN, Social Security Number.
