# Supplementary material for: A Web-Based, Provider-Driven Mobile App to Enhance Patient Care Coordination Between Dialysis Facilities and Hospitals: Development and Pilot Implementation Study
Source: JMIR Form Res. 2022 Jun 10;6(6):e36052. doi: 10.2196/36052 (PMC9233252; doi:10.2196/36052)
Supplement: Multimedia Appendix 2 [file formative_v6i6e36052_app2.docx]

**User testing feedback: DialysisConnect**

Research team members thoroughly evaluated the test version of the system, including the processes of admission, sending messages and documents, and discharge. In February 2020, the team presented the test system to the clinical teams at the hospital and dialysis facilities, with the intention of rolling out a live version of the system in the following month. Due to disruptions to clinical care early in the COVID pandemic, this rollout was delayed, and this time was used to add additional planned future upgrades to the system, perform user testing, and incorporate user testing feedback prior to rollout. Nine potential DialysisConnect users [two Emory University Hospital Midtown (EUHM) advanced practice providers (APPs), two EUHM hospitalists, two Emory Dialysis facility APPs, and three nephrologists (two at Emory Dialysis, one outside Emory)] participated in virtual user testing (led by L.C.P. and C.H.) with the research team in August–September 2020. All physicians were team members; with the exception of the external physician (B.G.J.), all were designated as DialysisConnect “site champions” (J.P.L. and T.M. at Emory Dialysis; C.M.O. and K.J. at EUHM). Each participant was asked to perform a standardized set of tasks on the test system, with guidance from the research team. Observations and feedback were collated (below) and provided to the technical team for a final round of fixes and updates to the system prior to rollout.

| **System issue** | **No. of testers having issue** |
| --- | --- |
| **Hospital users (*n*=4)** | |
| **Content** |  |
| “Hospitalized this month” shows a different number than if user runs a report for prior 30 days (especially if it is earlier in the month) | 2 |
| Nephrologist information is missing | 4 |
| Antibiotic route of administration: add i.v. | 4 |
| Antibiotic units: add g | 4 |
| Antibiotic frequency/duration: antibiotics may be started in hospital so it might unclear to dialysis facility when the duration is up, especially for long treatments (e.g., osteomyelitis) | 1 |
| Antibiotic required tests (for infectious disease physicians) | 1 |
| “Patient Communication” as a title for this function is confusing since it’s not with patient, but about the patient | 1 |
| Dialysis contact listed as user instead of actual contact | 1 |
| Add a list of routinely prescribed antibiotics | 1 |
| **Aesthetics** |  |
| Banner crowded content below | 2 |
| Banner jumbled (made it difficult to see report at the top) | 2 |
| Discharge button was hard to find (user expected it at bottom right) | 3 |
| Could not find “find patient” button | 1 |
| Discharge process was difficult to see on phone (e.g., like antibiotics where there are multiple columns) | 1 |
| **Functionality** |  |
| Could not change initial message at hospital admission | 1 |
| Hospital getting messages about events that they are initiating (that start “Hi Emory Dialysis”---might be confusing) | 1 |
| Meaning of flashing icon unclear | 2 |
| User could not find patient using lower case letters | 1 |
| Unclear that pages had to be refreshed to see changes/messages | 2 |
| Clicking on banners at top sent user to Oracle sign-in page | 2 |
| Receipt of message at the dialysis center is not clear | 1 |
| Confused current/previous hospitalizations | 3 |
| Unclear where to type secondary diagnosis | 1 |
| Emory EMR integration | 1 |
| Document upload: users had difficulty “linking” document after uploading | 1 |
| Users cannot send document without a request (nothing to link it to) | 2 |
| Sort option for discharge date was not clear/hard to find | 3 |
| Emails going to junk folder | 4 |
| Creating a link to system on phone | 1 |
| Would like to be able to review documents on phone | 1 |
| Unclear if kidney team at hospital can use the system (including antibiotic info on discharge)? | 2 |
| **Dialysis facility users (*n*=5)** | |
| **Content** |  |
| “161” showed up in antibiotics (first column) | 1 |
| SSN should be masked | 2 |
| Use number of documents required | 1 |
| Cross-covering nephrologist | 1 |
| Dialysis contact listed as user instead of actual contact | 1 |
| Route of admin: add i.v. | 1 |
| Timestamp on messages is wrong | 1 |
| Recommended having the patient medication end/stop date listed | 1 |
| Recommended adding the “bug” next to the antibiotic name like “Pseudomonas” | 1 |
| **Aesthetics** |  |
| Banner jumbled (made it difficult to see the report at the top) | 2 |
| Secondary diagnoses in discharge “report” were crowded (had to expand) | 1 |
| Icon (like app) vs. bookmarking page | 2 |
| Number of documents required | 1 |
| Icons are small for phone or iPad (using finger), needs enlarging | 2 |
| **Functionality** |  |
| Sorting function in list of hospitalizations is not obvious | 3 |
| “Other” reason for hospitalization not coming through from hospital | 5 |
| “Changes to dry weight” in the discharge information (either “yes” or the actual weight) were not sent to dialysis facility (they received “no changes”) | 5 |
| How to respond to messages was not clear | 3 |
| Connection to EMR unclear | 1 |
| Having admins on dialysis/hospital side upload documents | 2 |
| Clinicians will be on virtual desktop, cannot pull documents from there | 1 |
| Confusion about whether links in texts/emails go to home page or to the event | 1 |
| Emails going to the junk folder | 4 |
| Workaround for not having nephrologists on both sides (due to requirement for unique usernames), having APP add dry weight/dialysis changes while hospitalists do the rest | 1 |
| Text only for admission/discharge, and document sent, but not document request from other side | 1 |
| Have hospital automatically upload run sheet if there are changes to dialysis | 1 |
| Order of new messages is “documents required” at top; should this be “most recent first”? | 1 |
| Confusion on current hospitalizations tab to find new patient | 1 |
| Difficulty finding the messages | 1 |

APP, advanced practice provider; EMR, electronic medical record; SSN, Social Security Number.
