# Supplementary material for: A Web-Based, Provider-Driven Mobile App to Enhance Patient Care Coordination Between Dialysis Facilities and Hospitals: Development and Pilot Implementation Study
Source: JMIR Form Res. 2022 Jun 10;6(6):e36052. doi: 10.2196/36052 (PMC9233252; doi:10.2196/36052)

**Detailed screenshots showing features of the DialysisConnect system**

**DialysisConnect home (landing) page:**


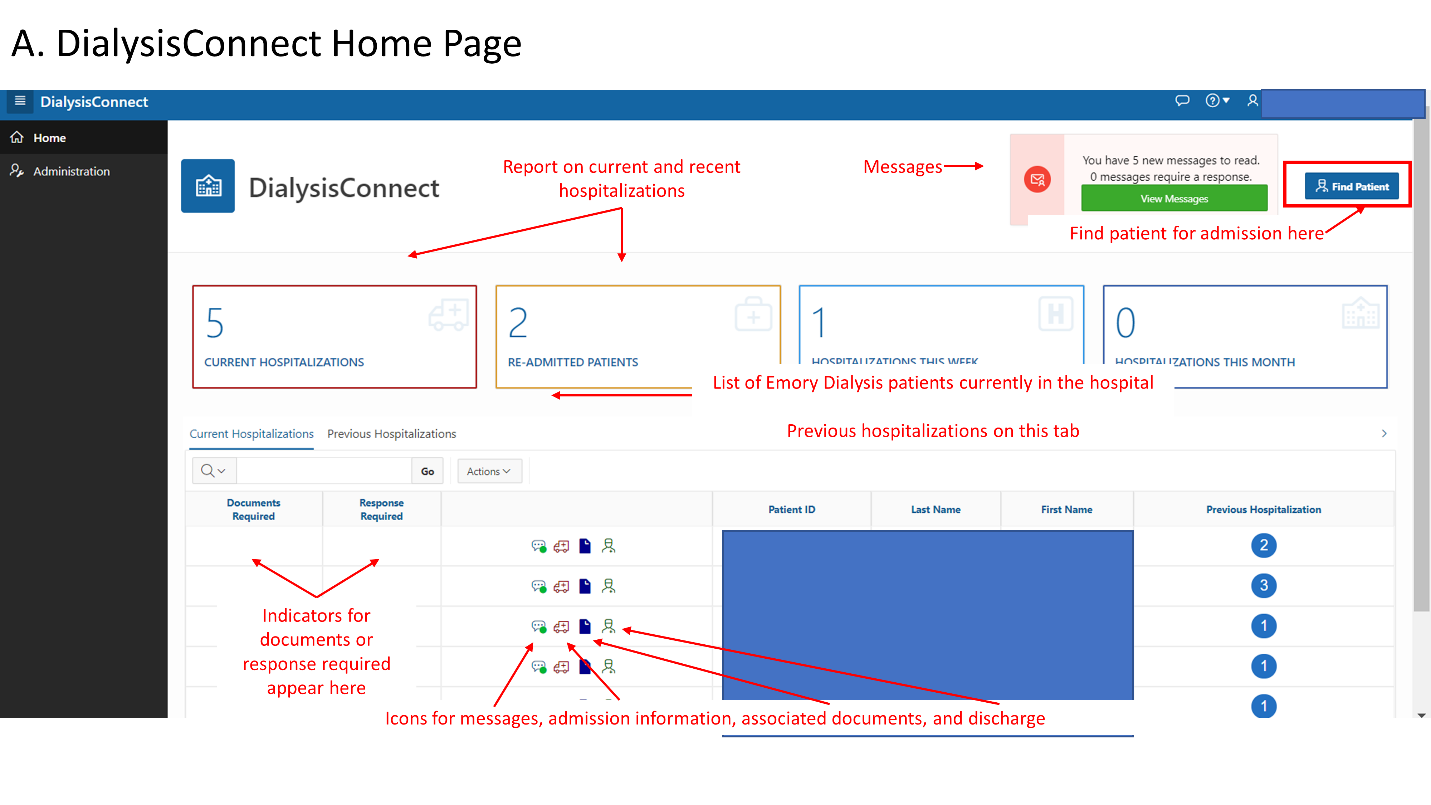


**Admission page (hospital providers):**


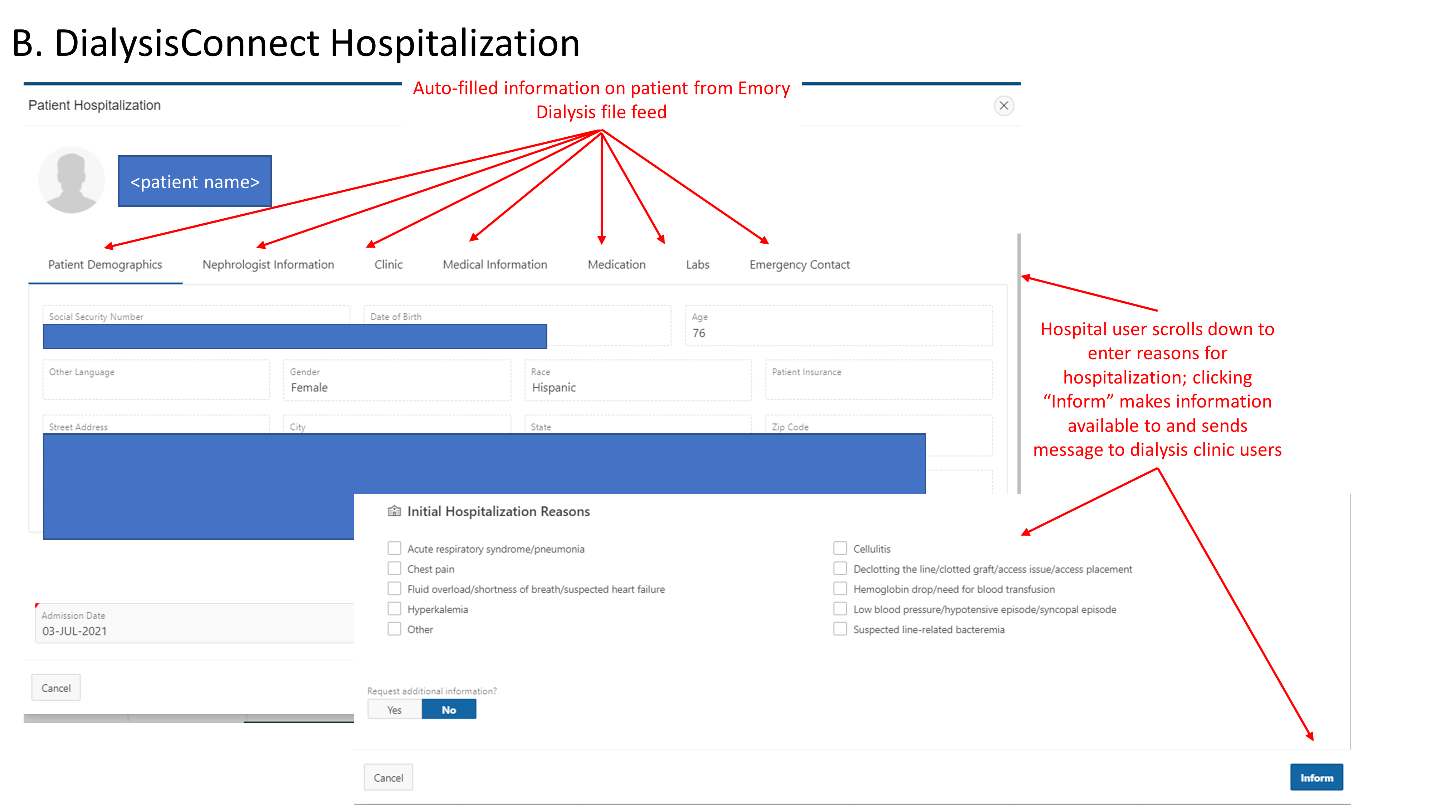


**Message page (all users):**


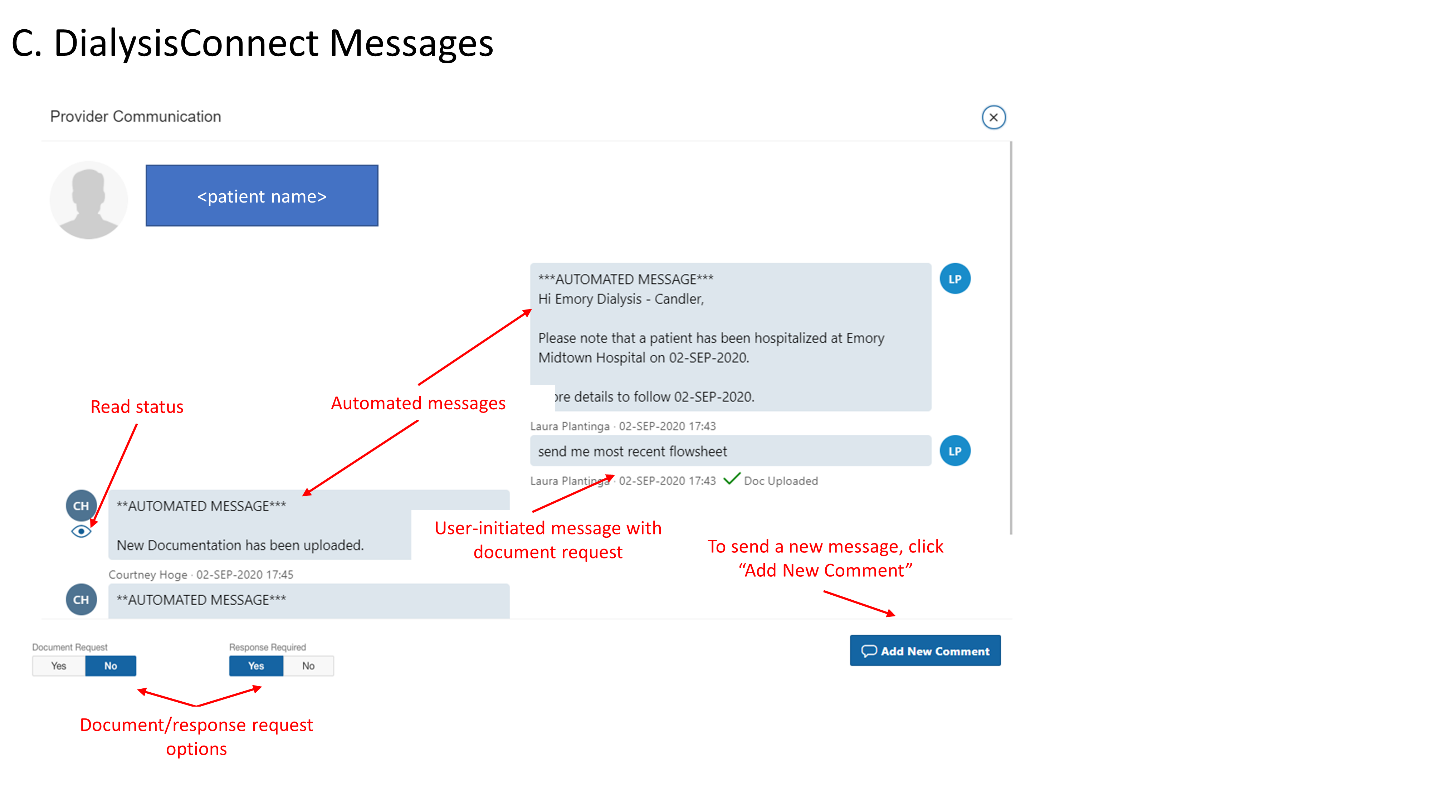


**Discharge page (hospital users):**


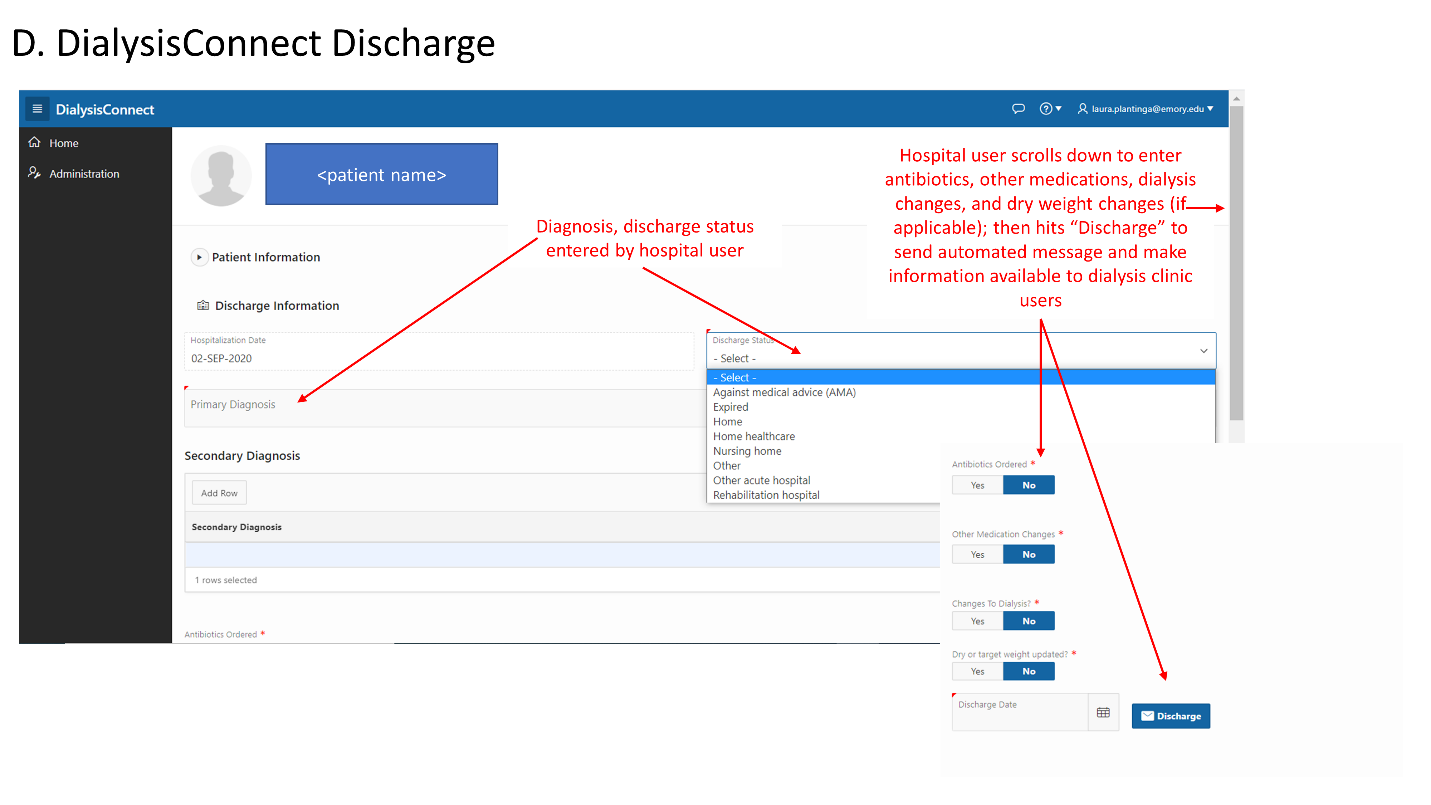

Supplement: Multimedia Appendix 3 [file formative_v6i6e36052_app3.docx]
