# Supplementary material for: A Web-Based, Provider-Driven Mobile App to Enhance Patient Care Coordination Between Dialysis Facilities and Hospitals: Development and Pilot Implementation Study
Source: JMIR Form Res. 2022 Jun 10;6(6):e36052. doi: 10.2196/36052 (PMC9233252; doi:10.2196/36052)
Supplement: Multimedia Appendix 4 [file formative_v6i6e36052_app4.pdf]

A Quick Start Guide for Hospital Users

Version 2.0, 4/14/21

Table of Contents

Part 1. About DialysisConnect.......3

What is DialysisConnect?.......3

How was DialysisConnect developed?.......3

Can we propose changes to DialysisConnect?.......3

Is DialysisConnect part of the EMR?.......3

Who is using DialysisConnect?.......3

How will I know that a patient is from Emory Dialysis?.......3

Where can I find DialysisConnect?.......4

How do I access DialysisConnect?.......4

Will I be able to use DialysisConnect after the pilot study is over?.......4

What browser should I use to view DialysisConnect?.......4

Can I use my mobile phone to view DialysisConnect?.......4

Can I create an icon on my laptop/computer or smartphone that goes directly to DialysisConnect?.......5

Will DialysisConnect "time out" if I am logged in but inactive?.......5

How will DialysisConnect let me know there is a request for information to review?.......5

Contact us.......6

Part 2. How to Use Dialysis Connect.......7

Explore the home page.......7

What happens when a dialysis patient is admitted.......7

Step 1: Confirm the patient is an Emory Dialysis patient.......7

Step 2: Inform the dialysis clinic that a patient is being admitted.......9

Step 3: Sending messages and documents.......10

Step 4: Discharging the patient.......18

Acknowledgements.......21

Study Team.......21

Study Funding.......21

Part 1. About DialysisConnect

What is DialysisConnect?

DialysisConnect is a secure, HIPAA-compliant, web-based platform to facilitate coordination of care of dialysis patients during and after hospitalizations.

How was DialysisConnect developed?

We have developed DialysisConnect through an iterative process, through the ongoing feedback of our study team (which consists of hospitalists and nephrologists as well as scientists and developers); feedback on an initial proposed system via focus groups of Emory University Hospital Midtown (EUHM) and Emory Dialysis staff involved in care transitions of dialysis patients; and feedback during user testing of the working beta system.

Can we propose changes to DialysisConnect?

Yes. The iterative nature of DialysisConnect development means that we can make changes and enhancements during the pilot phase. It is important to note that some proposed changes may be too intensive to roll out quickly during the pilot phase; however, feedback from users during the pilot phase will be collected and used to develop the next version(s) of DialysisConnect. We will also seek feedback during the pilot phase via brief online provider surveys.

Is DialysisConnect part of the EMR?

No. At this time, DialysisConnect is a stand-alone system that is not integrated with Emory's electronic medical record (EMR). However, we hope this pilot study will provide evidence that this system can help improve patient outcomes and support this eventual integration.

Who is using DialysisConnect?

While we eventually aim to roll Dialysis Connect out to a larger group of providers, for this pilot study, only *hospital providers at EUHM* and *dialysis providers at Emory Dialysis* are using DialysisConnect. Thus, for the pilot study period (6 months), the system will only be used for patients who are admitted to EUHM and are receiving dialysis at an Emory Dialysis facility (Northside, Candler, Greenbriar, and North Decatur) upon hospital admission.

How will I know that a patient is from Emory Dialysis?

You will be able to either confirm the patient's status as a current patient at one of Emory Dialysis' clinics (if the patient or surrogate has indicated they are treated at Emory Dialysis), or search for the patient (if the patient or surrogate does not know or cannot communicate their dialysis clinic at

admission). This information will be updated by the technical team at least once per month. If a patient has very recently begun treatment at Emory Dialysis, it is possible they will not yet appear in the system.

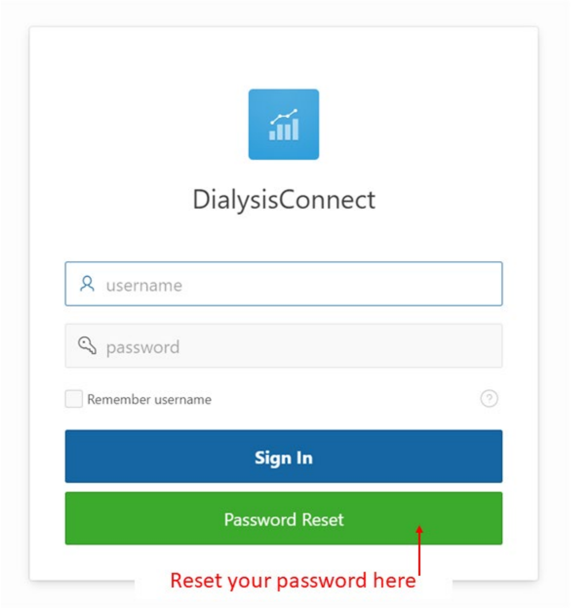

Where can I find DialysisConnect?

DialysisConnect is a web-based application that can be found at the following URL: https://dialysisconnect.com/. This page may be bookmarked. Some browsers also allow webpages to be pinned to the Start menu or added to the Desktop. Please contact us if you would like to do this and need help.

How do I access DialysisConnect?

Once you navigate to the DialysisConnect page, you will enter your username and password to enter the system (left). Your username is your email address. Your password will be assigned when you are added to the system; you can change this by clicking on "Password Reset."

The site is only available to identified users of the system. If you would like to be added as a user, or are having trouble accessing the system, please contact us.

Will I be able to use DialysisConnect after the pilot study is over?

Yes, DialysisConnect will remain available after the pilot study data collection period ends. However, technical support may be limited until or unless follow-up funding is secured.

What browser should I use to view DialysisConnect?

DialysisConnect was developed on Chrome and thus is optimally viewed with this browser. **Please note that DialysisConnect will not function on Internet Explorer 11** (which is no longer supported by Microsoft); you will be re-directed if you attempt to use Internet Explorer 11.

Can I use my mobile phone to view DialysisConnect?

Yes. However, note that pages configured for mobile phones are only available through links in SMS messages. If you log into DialysisConnect from a browser on your phone, the system will be visible and functional, but the pages will not be specifically configured for mobile phone viewing.

Can I create an icon on my laptop/computer or smartphone that goes directly to DialysisConnect?

Yes. You can create a shortcut on your desktop or on your phone's home screen to the DialysisConnect website, as shown below. If you need help adding an icon on your Android phone, please [contact us].

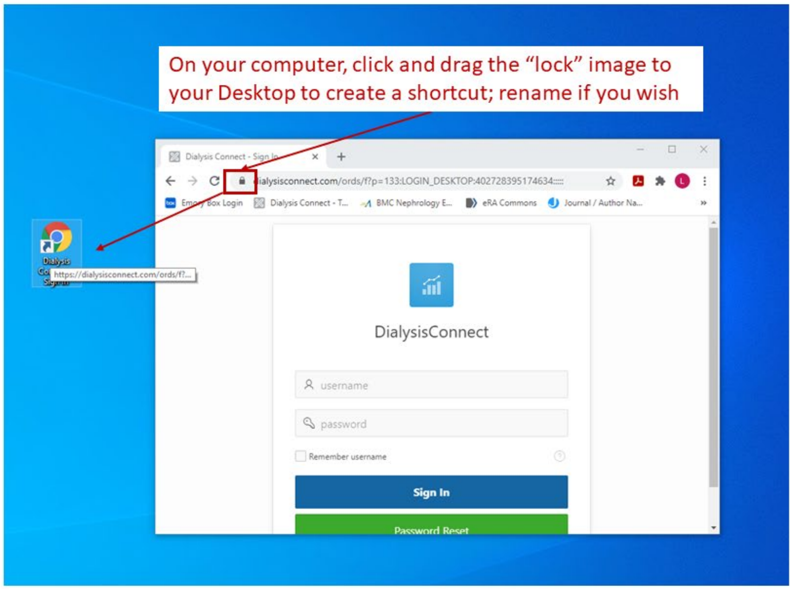

On your iPhone, with the website open in Safari, click "Share" at the bottom of the screen and then select "Add to Home Screen" in the popup window

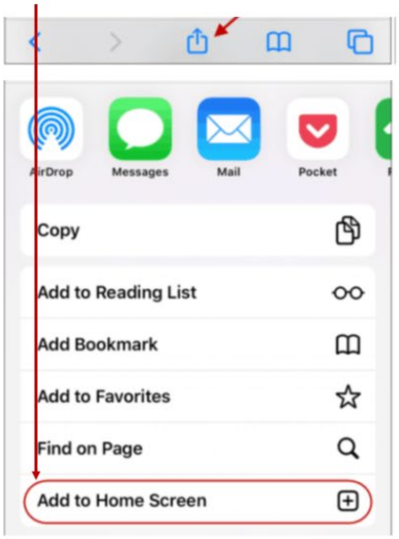

Will DialysisConnect "time out" if I am logged in but inactive?

Yes, to maintain security of the system, DialysisConnect will time out after **20 minutes** if the user is inactive. Information entered that has not already been submitted will be lost. A pop-up warning will let you know.

How will DialysisConnect let me know there is a request for information to review?

You can allow DialysisConnect to send automated email or SMS (or both) messages when the dialysis facility sends a request for information. If you would like to add your mobile number, please [contact us].

Because Emory Healthcare and University will not permanently “whitelist” these addresses, you may find the emails go to your junk folder. To add these automated emails to your “safe sender list” go to junk e-mail options and add the domain “@dialysisconnect.com”:

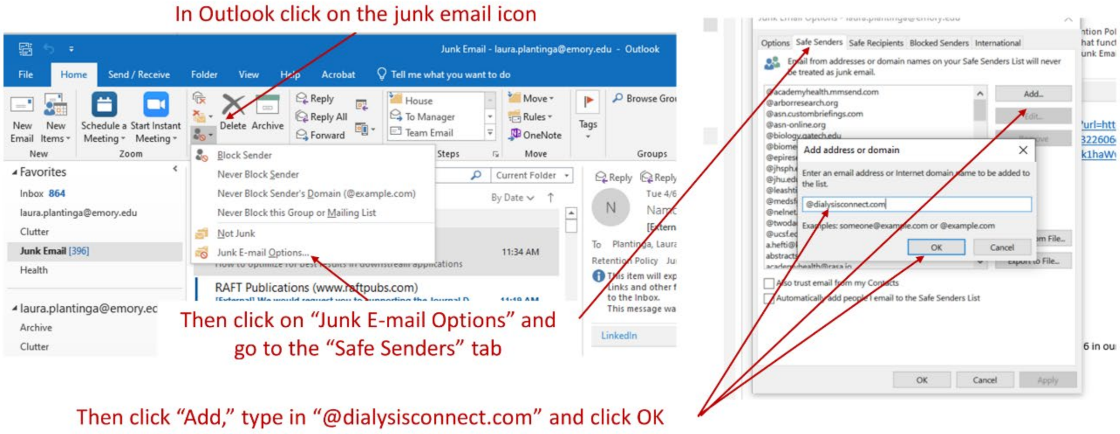

Contact us

If you have questions about the DialysisConnect pilot study or how to use the system, or you would like to request individual training:

Laura Plantinga, Principal Investigator, laura.plantinga@emory.edu
Courtney Hoge, Project Manager, courtney.hoge@emory.edu

If you have technical questions about DialysisConnect system:

dialysisconnect@apexhealthinnovations.com

Part 2. How to Use Dialysis Connect

Explore the home page

Once you log in, you will see the home page.

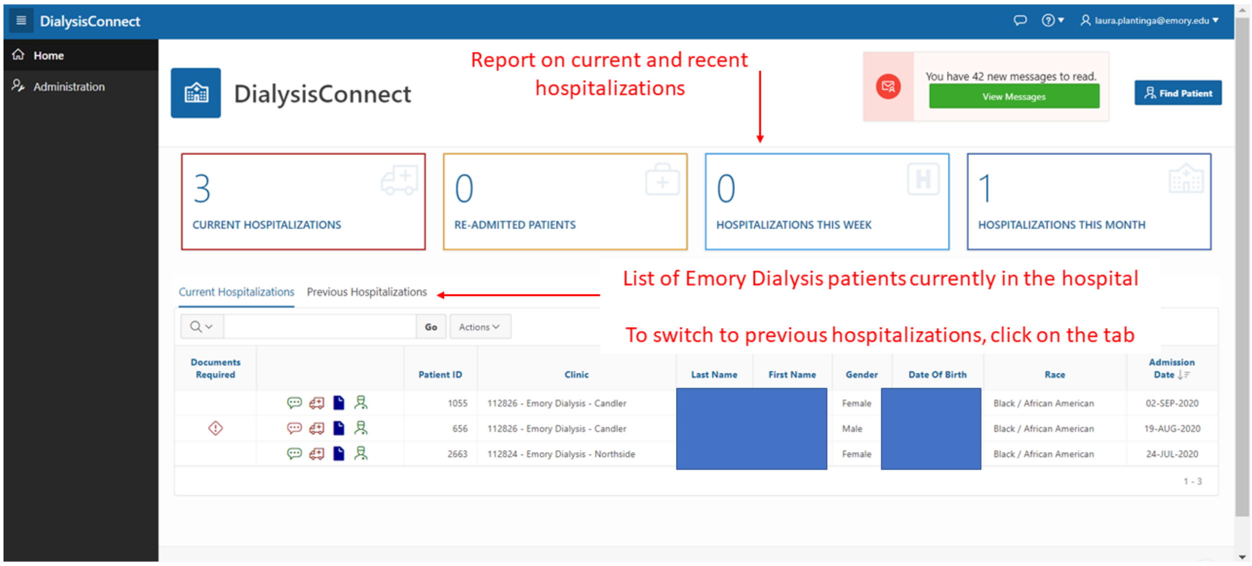

Here you will see an overview report showing the number of Emory Dialysis Patients currently in the hospital, the number re-admitted within 30 days, and the numbers of hospitalizations in the current week and month. You will also see a list of Emory Dialysis patients currently in the hospital (including their clinic and some demographic information, along with their admission dates). Previous hospitalizations can be seen by clicking the “Previous Hospitalizations” tab.

From the home page, messages, hospitalization information, documents received and discharge information (previous hospitalizations only) can be accessed through the text, ambulance, document, and doctor icons, respectively. These will be described in more detail under tasks.

What happens when a dialysis patient is admitted

Step 1: Confirm the patient is an Emory Dialysis patient

If the patient is known to receive dialysis outside of Emory Dialysis, you <u>do not</u> need to use DialysisConnect.

If you know the patient is from Emory Dialysis, *or* if the clinic is unknown, then the first step is to confirm this status in DialysisConnect, using the “Find Patient” button:

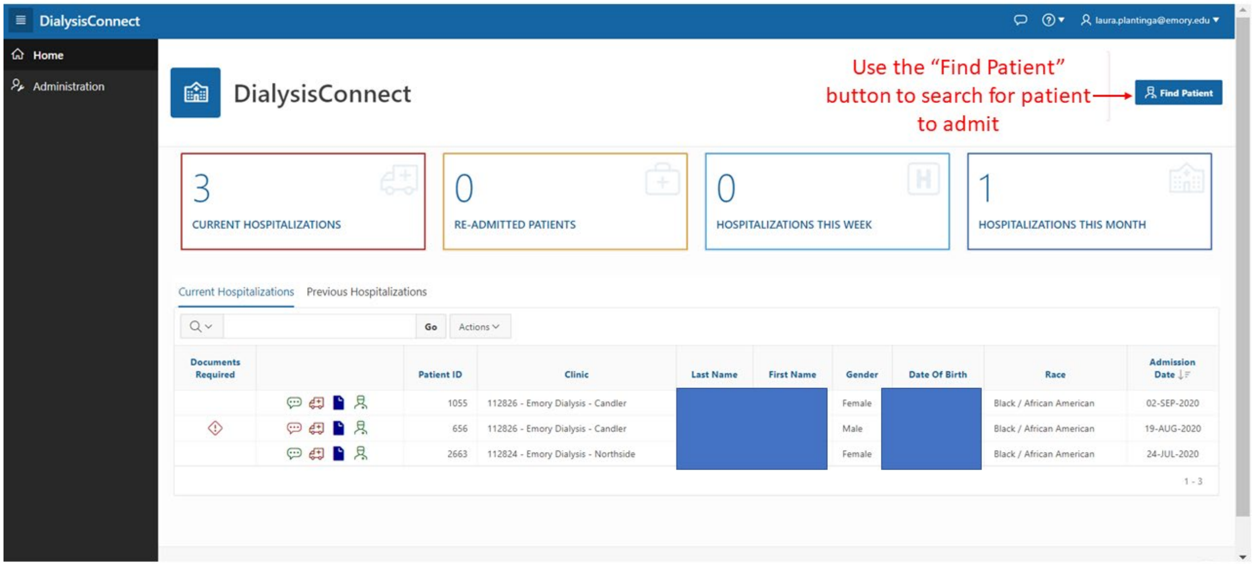

This will open a search window where you can search the patient’s name. Names will autofill as you type and you will be able to select the patient if they are in the system.

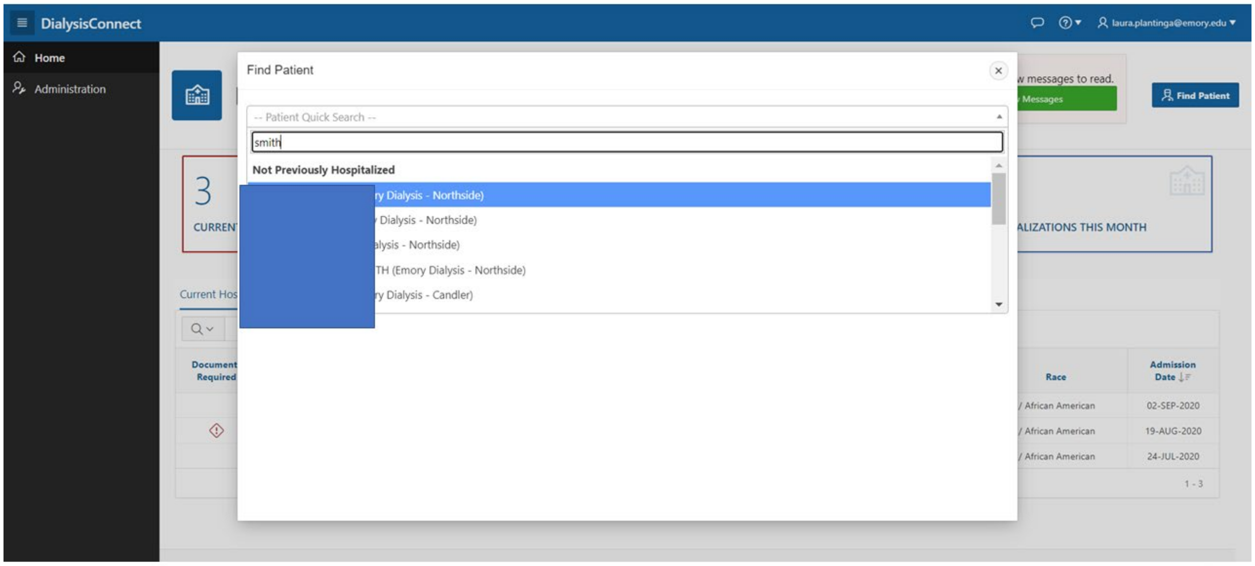

If the patient is not found, this means they are not currently a patient at Emory Dialysis (or have started treatments so recently that they are not found in the patient census). There is no more to do for this patient during this admission.

<u>If the patient is found</u>, the next step is to note the reasons for admission and inform the dialysis clinic.

Step 2: Inform the dialysis clinic that a patient is being admitted

Once you select the patient from the drop-down list above, a new window opens. This window includes auto-filled information about the patient from the dialysis clinic:

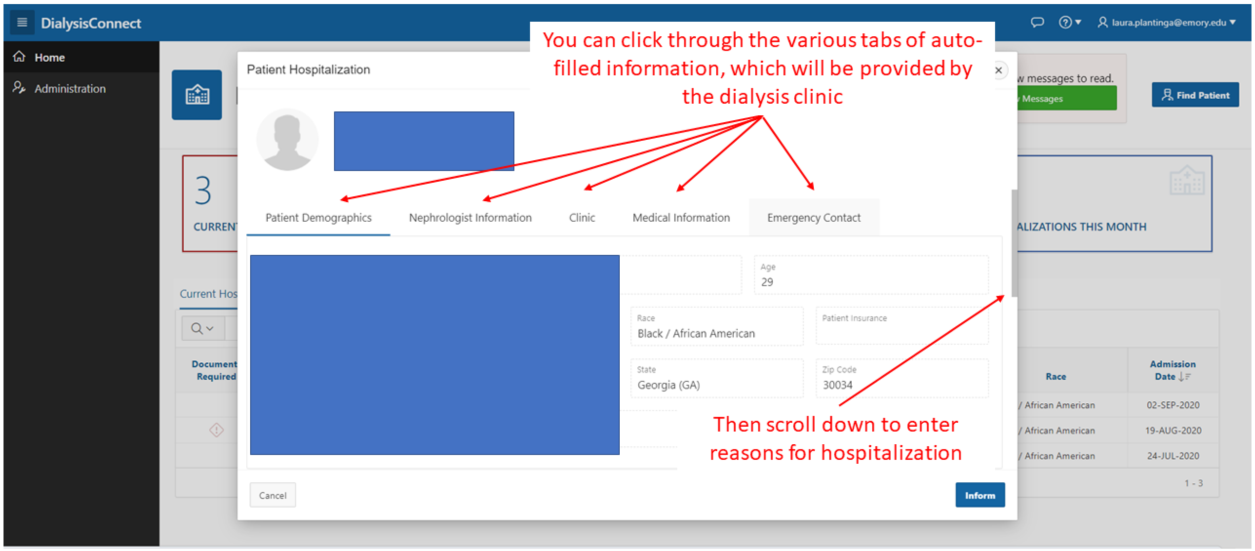

This popup window also allows you to enter the reasons for the current admission and inform the dialysis clinic:

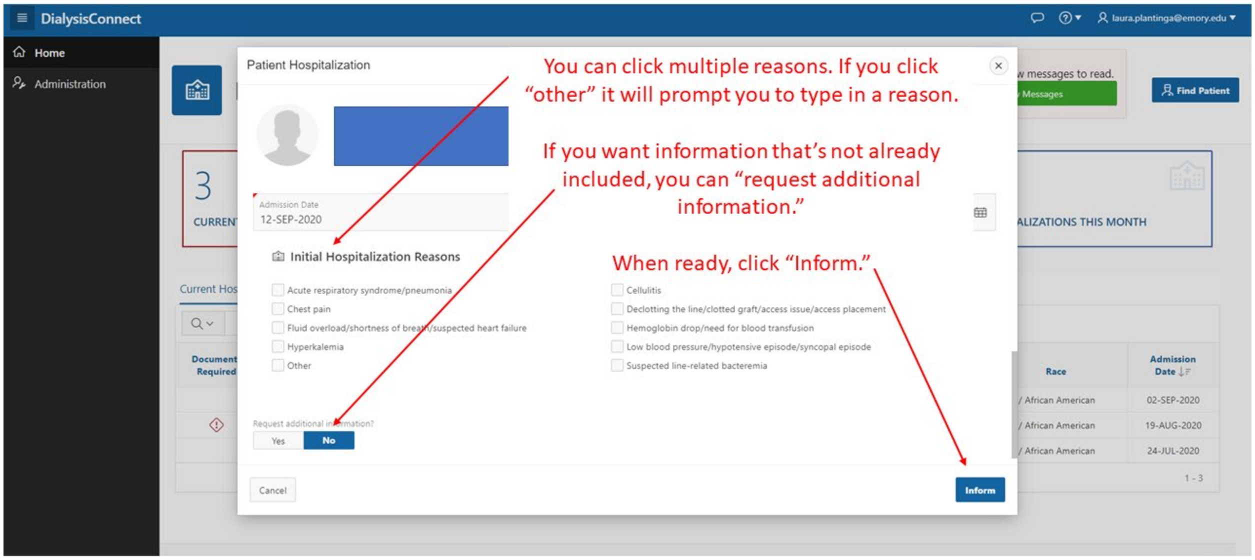

When you click inform, appropriate personnel on the dialysis side instantly receive a message that a patient has been admitted to EUHM, and they are prompted to log in to DialysisConnect review the information. You will also see that the patient is now on the "current hospitalizations" list. If you

indicate that you would like additional information at this stage, you will be prompted to enter a message to describe your request.

Note that the information about the patient can be accessed at any time from the "ambulance" icon from the home page:

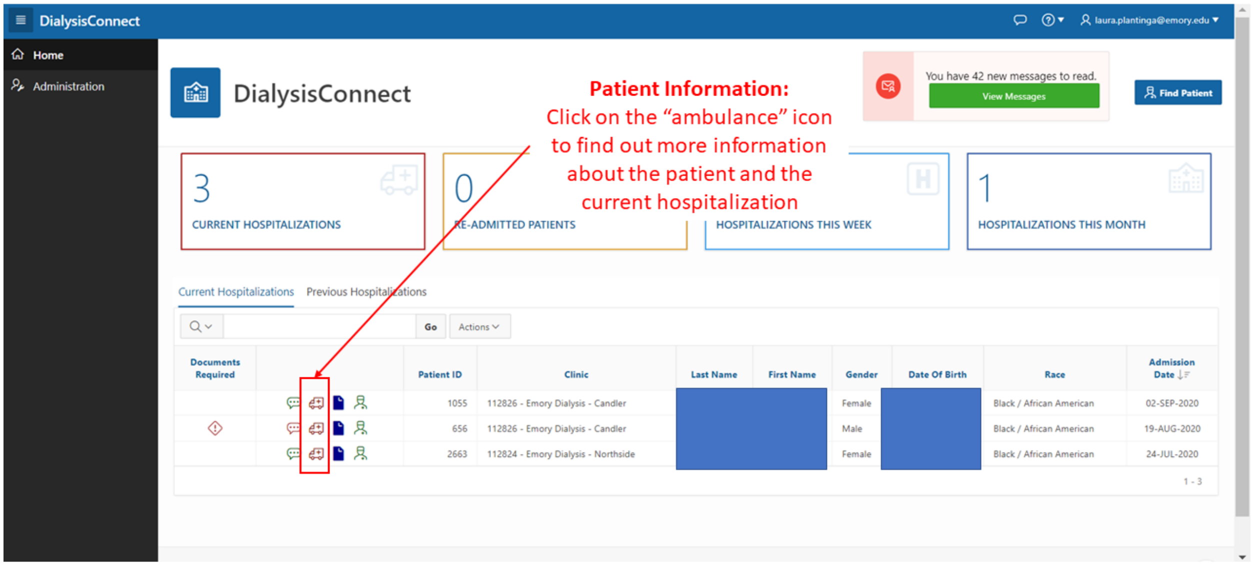

Step 3: Sending messages and documents

While the patient is in the hospital, there may be no DialysisConnect tasks until discharge. However, if you would like more information from the dialysis clinic about a patient, you can send a message at any time. Messages can be accessed from the "text bubble" icons or the message box:

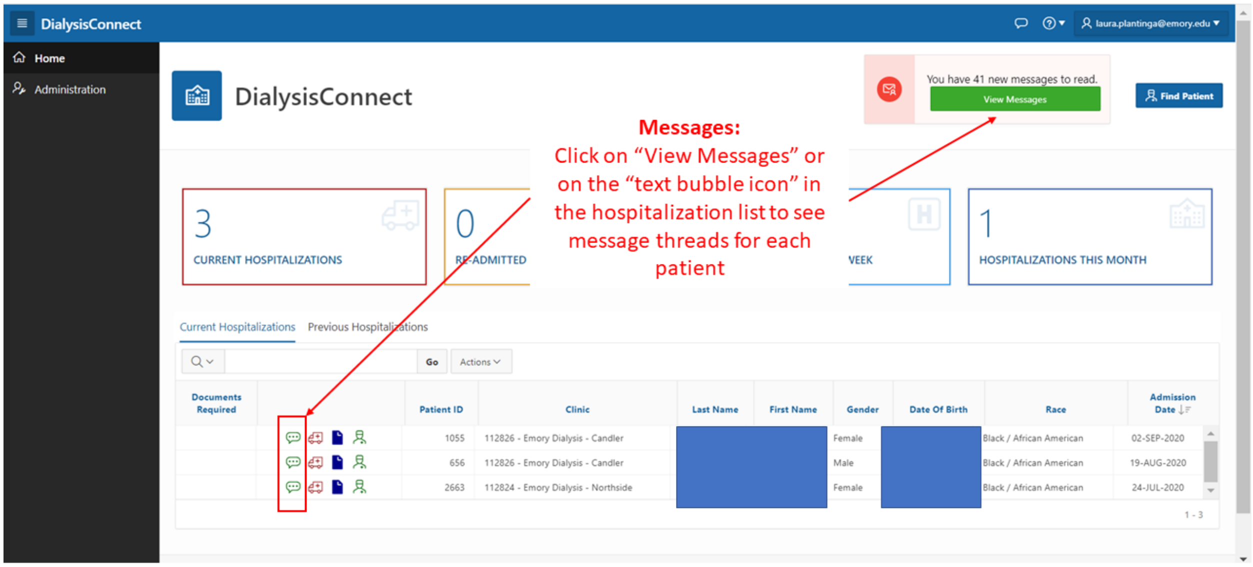

Messages will pop up and will look like a text message exchange:

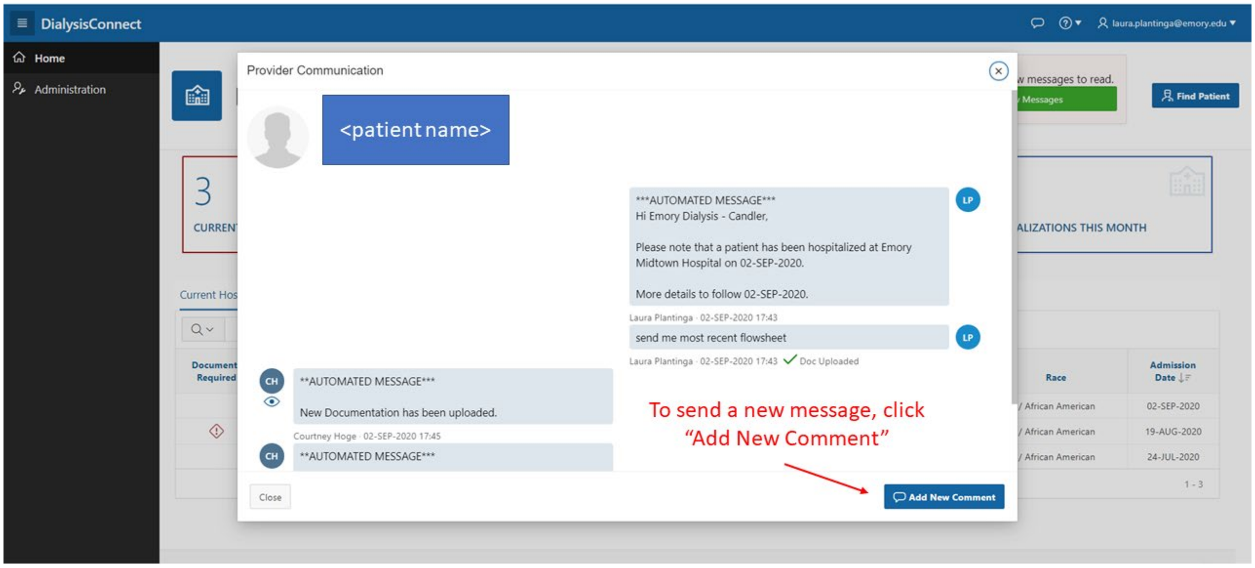

You can click "Add New Comment" to create a new message. If you are requesting a document, you can indicate that before clicking "Create":

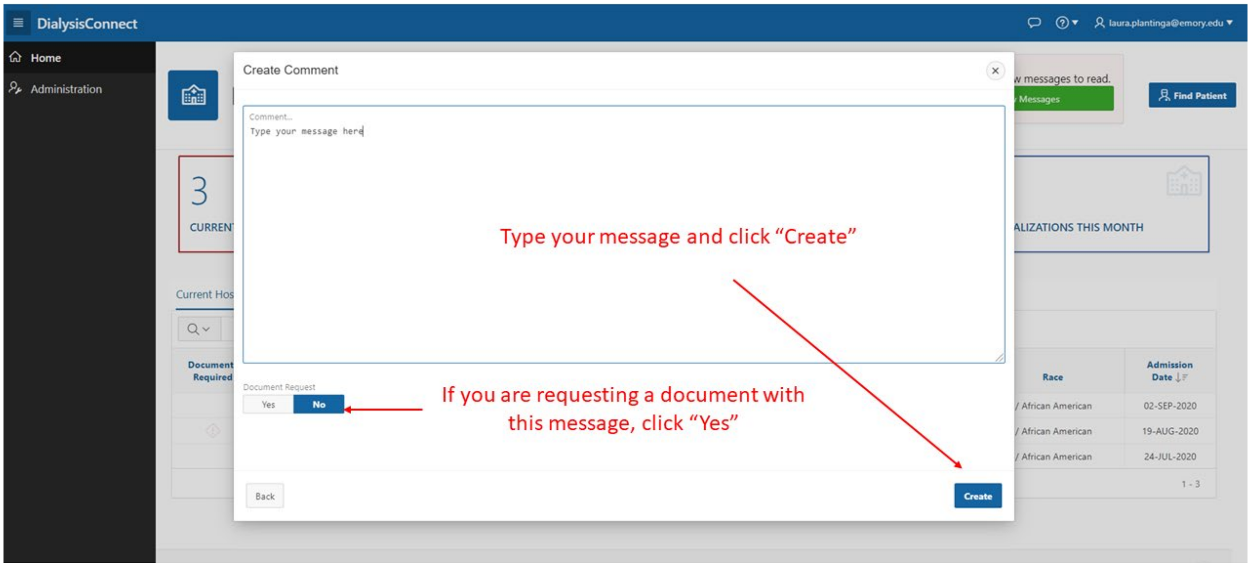

If you like to know whether your message to the dialysis clinic was "read," look for the eye icon next to the message:

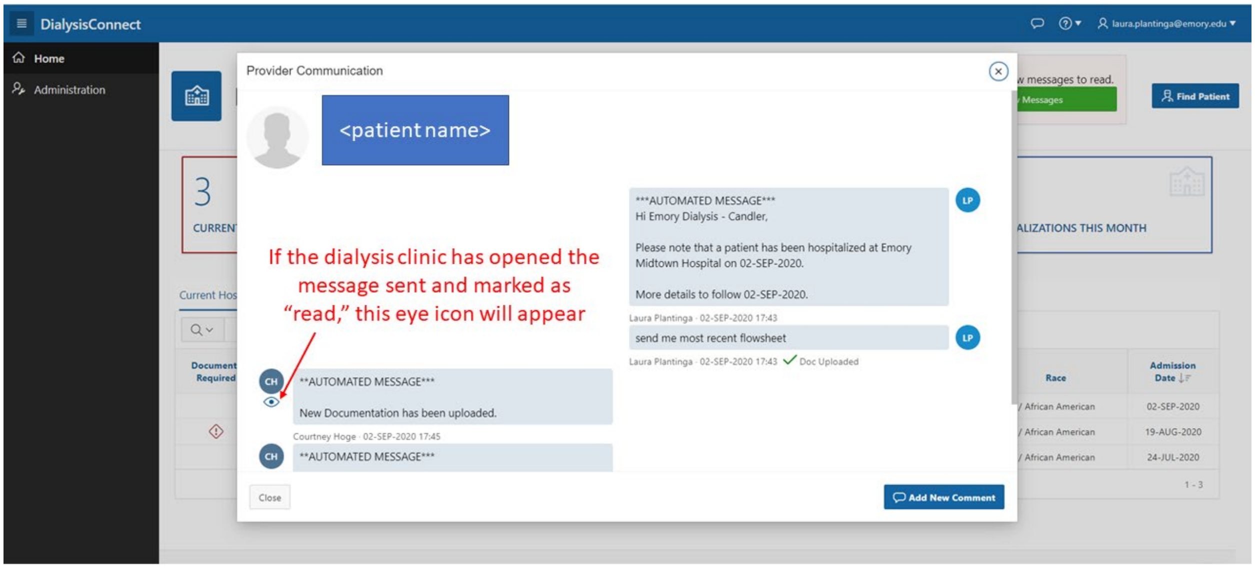

Dialysis clinics may also send you messages. When the dialysis clinic requests a document, you will receive a message by e-mail, SMS text messaging, or both, depending on your preferences for communication in the system. You can either click on the link from the message to log in, or navigate to the URL and log in.

Remember if you are on your mobile phone and do not use the link provided in the message (right), you will go to a page that is not configured for the mobile phone.

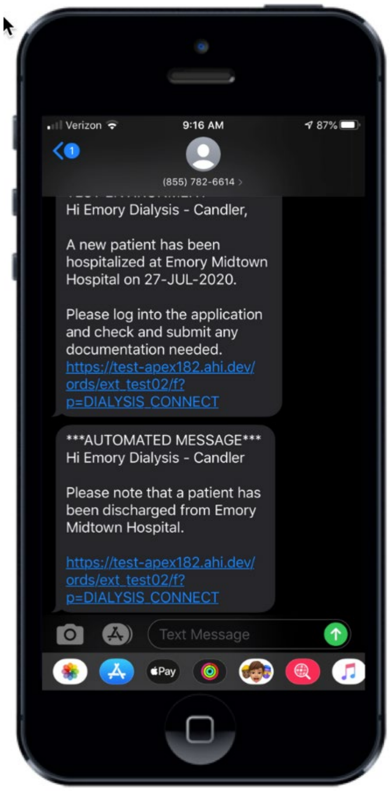

To indicate to the dialysis clinic that their message was opened and read, you will "mark all messages as read" for that thread:

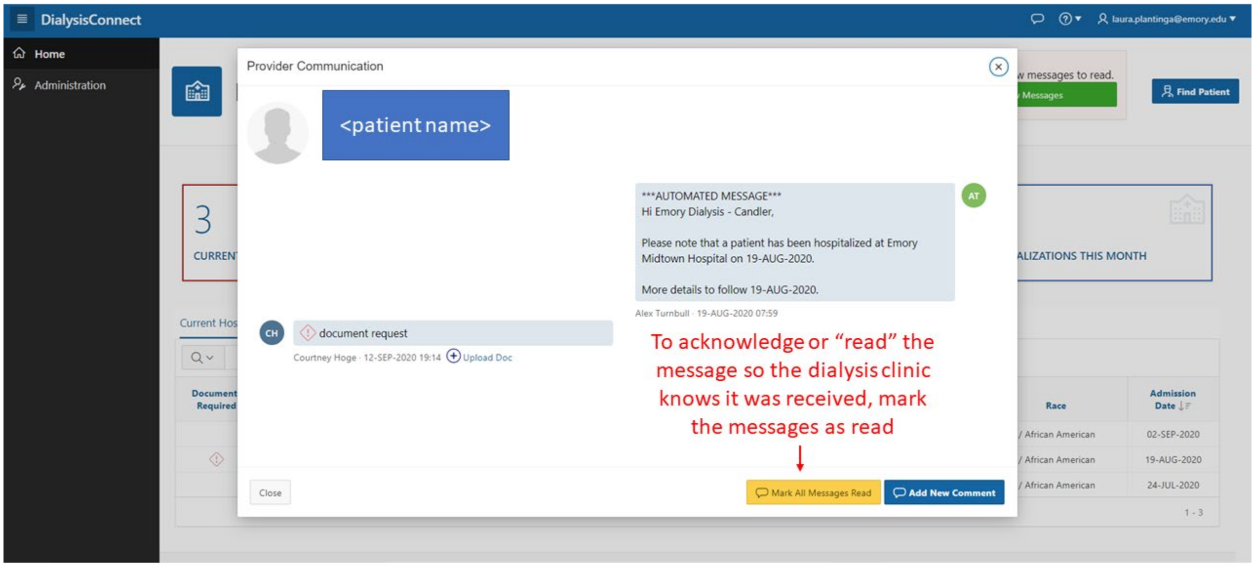

To access documents, you may click "Upload Document" directly from the message:

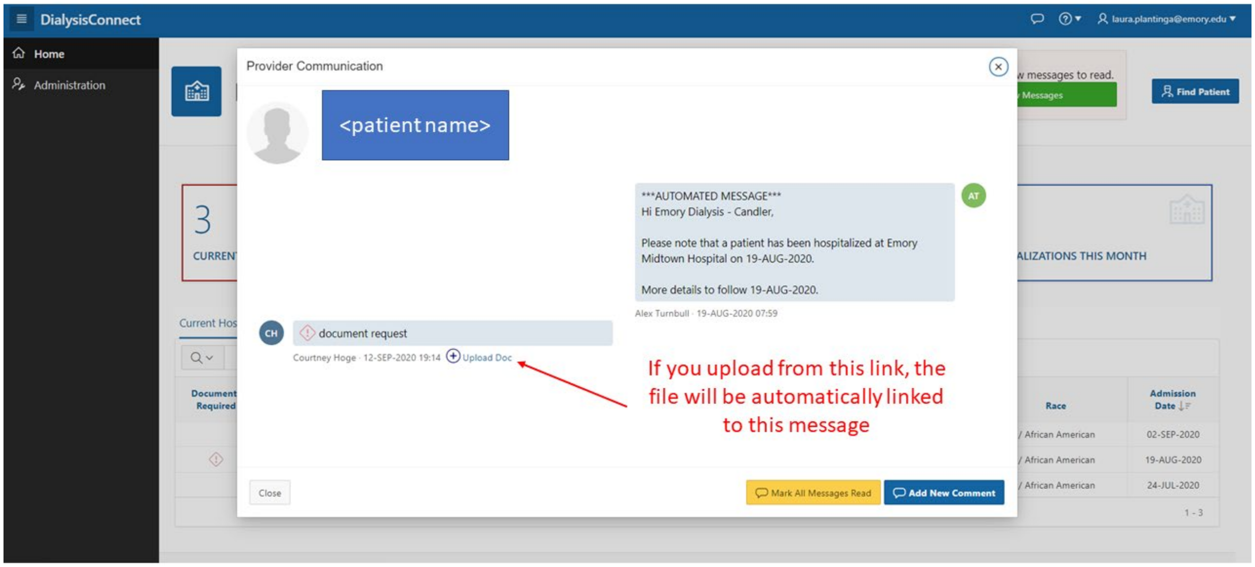

Or, you can navigate to documents:

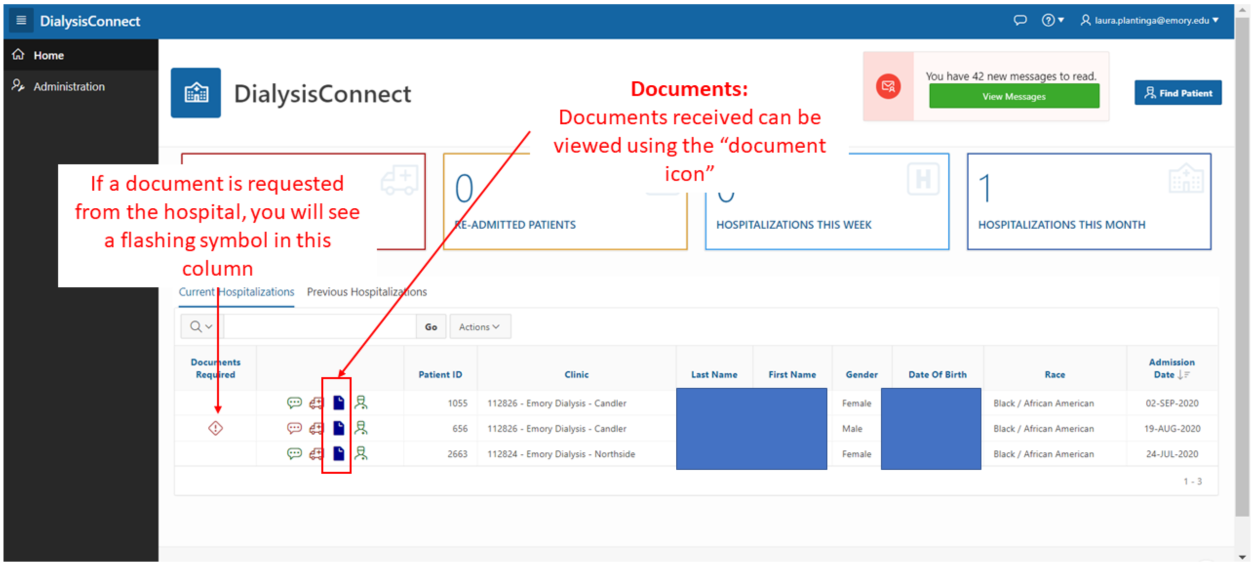

Then upload the document, and link to a message:

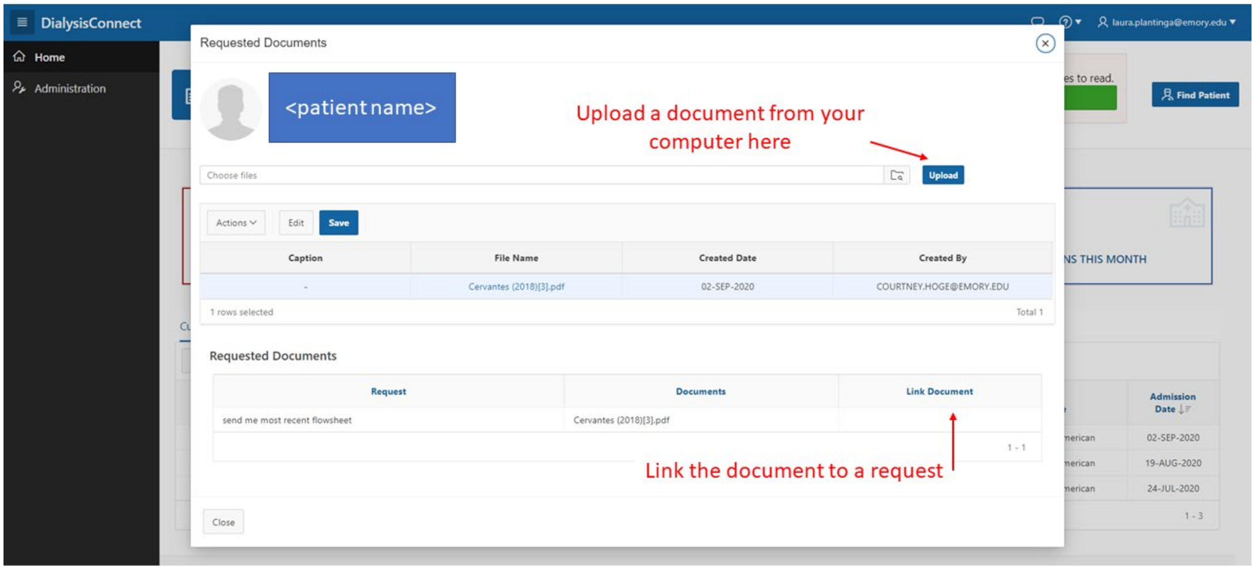

You may wish to request that the dialysis clinic confirm that information in a message is being acted upon; for this you may wish to add a “Response Required” tag to your message:

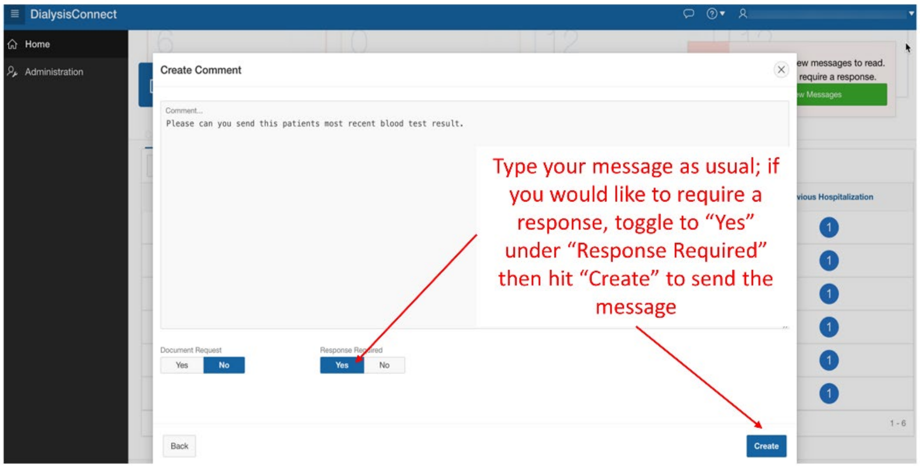

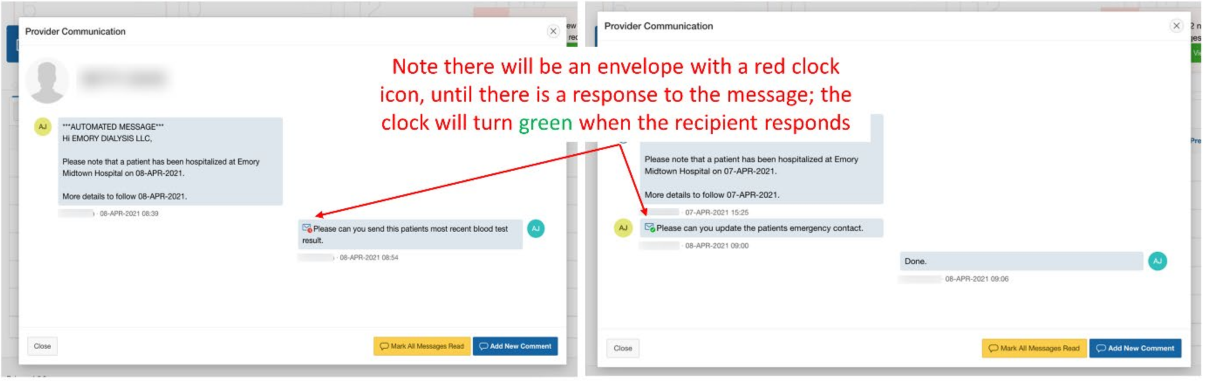

The dialysis clinic may also send a “Response Required” message to you. These messages will appear on your home page:

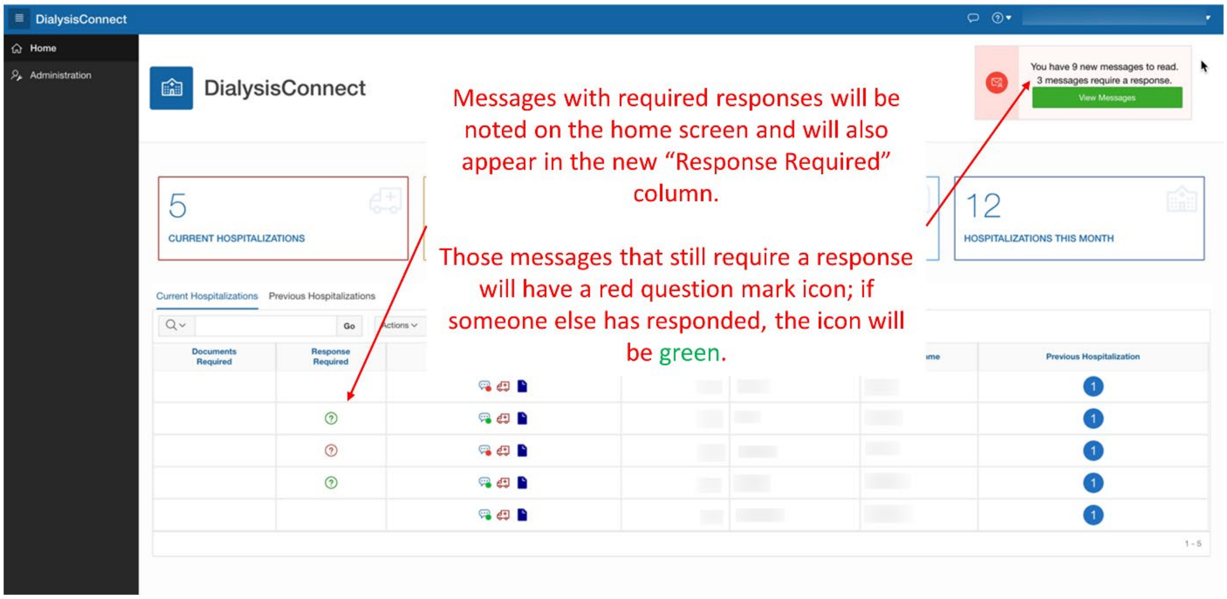

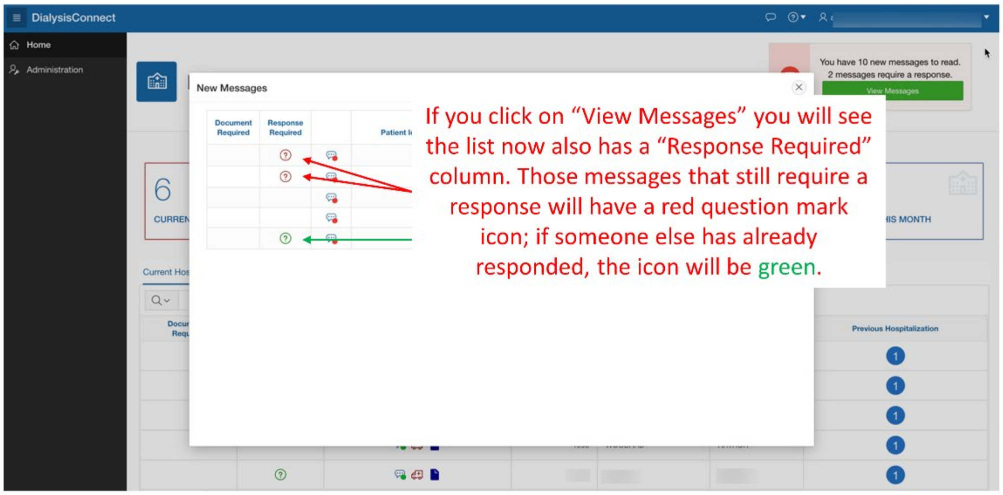

To respond to a message with a “Response Required”:

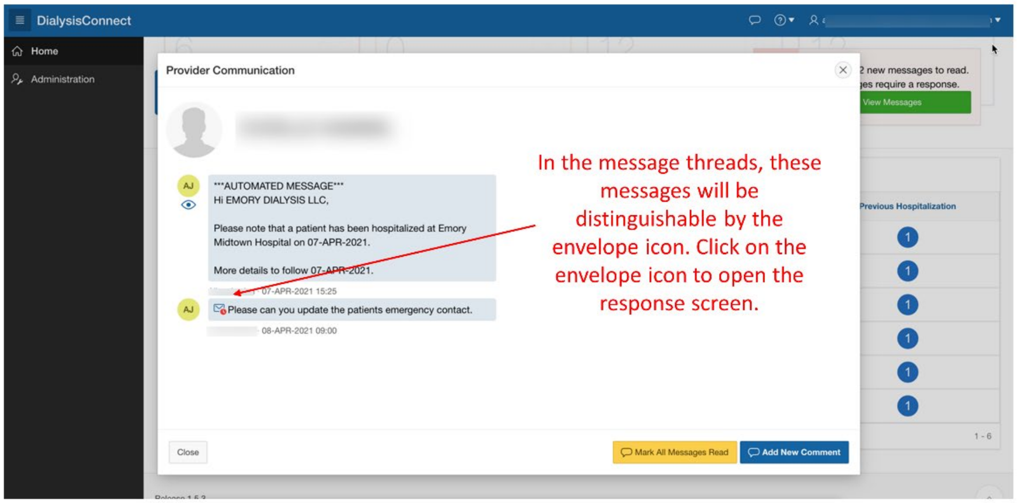

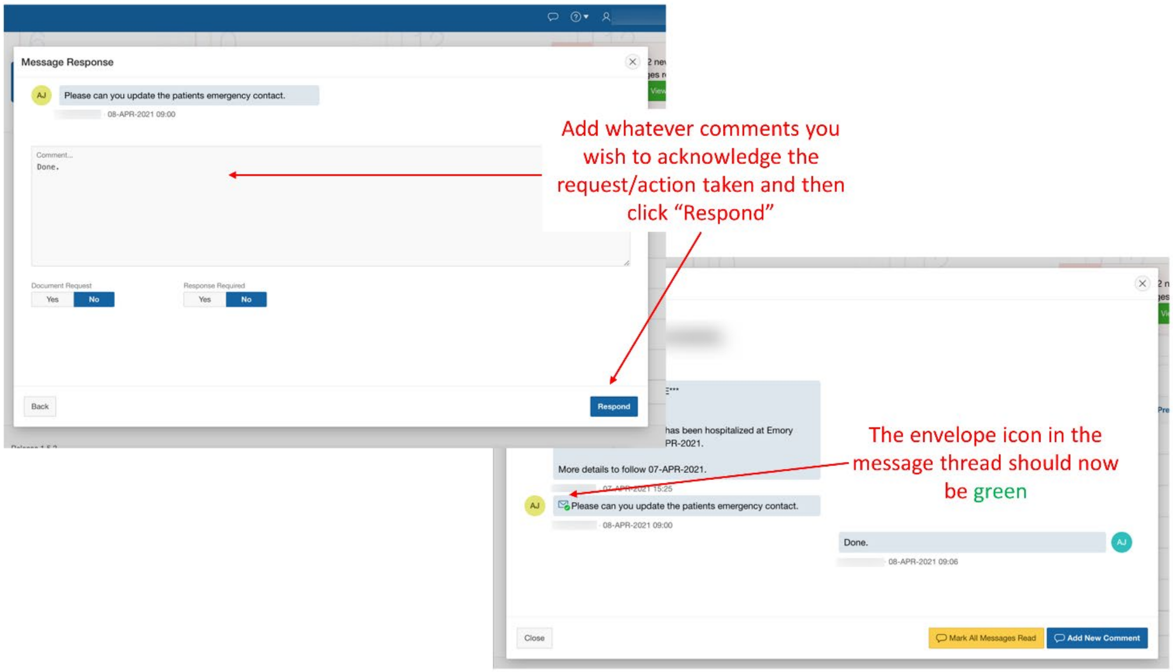

Step 4: Discharging the patient

When the patient is discharged, we ask that you let the dialysis clinic know and also enter some brief, critical information about the hospitalization to assist with the care transition. While they will receive a discharge summary, and some providers can access the EMR to view the summary as soon as it is ready, complete discharge summaries are often delayed, resulting in many patients arriving for dialysis with no information about their hospitalization event.

To initiate a discharge, you can click the “doctor” icon on the home page.

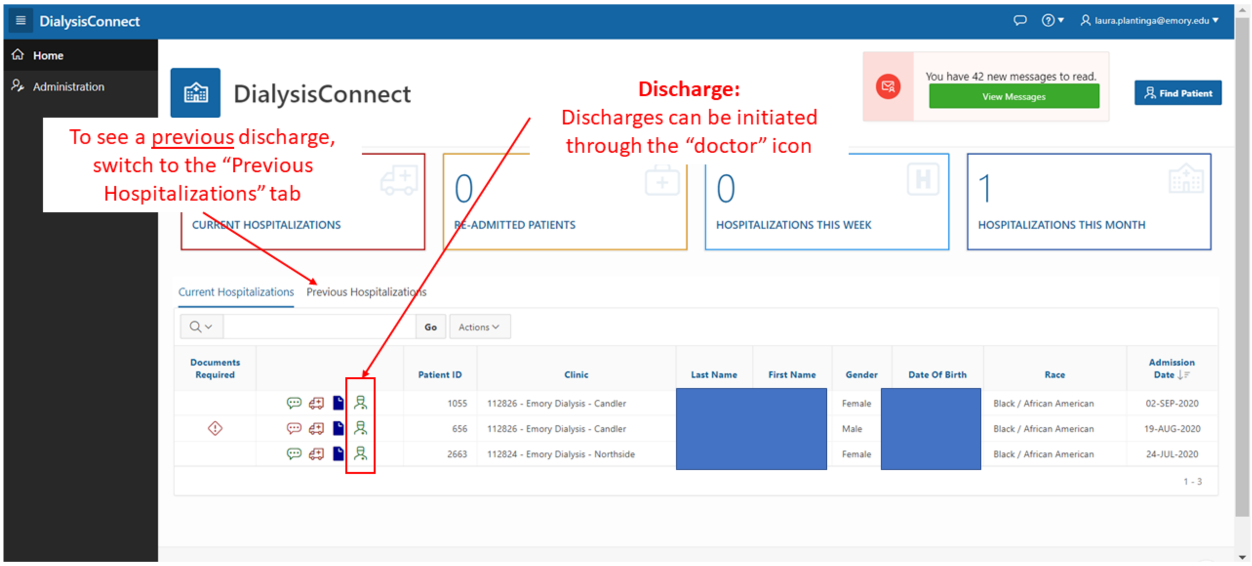

Discharge is also available through the hospitalization information tabs:

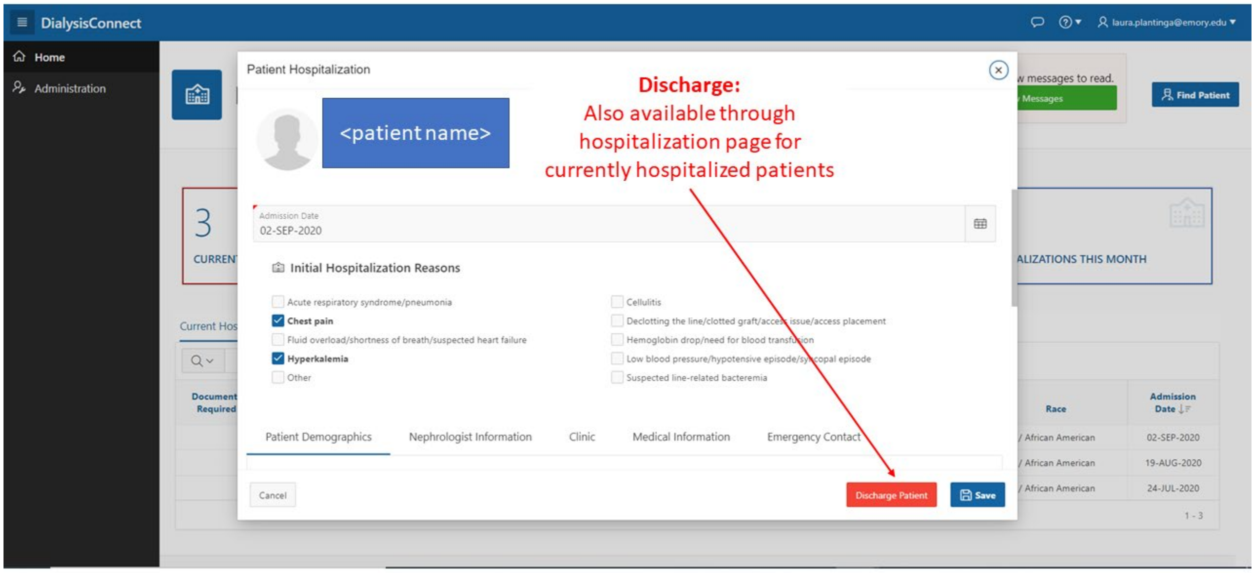

Either method will open a window to enter information and "discharge" the patient:

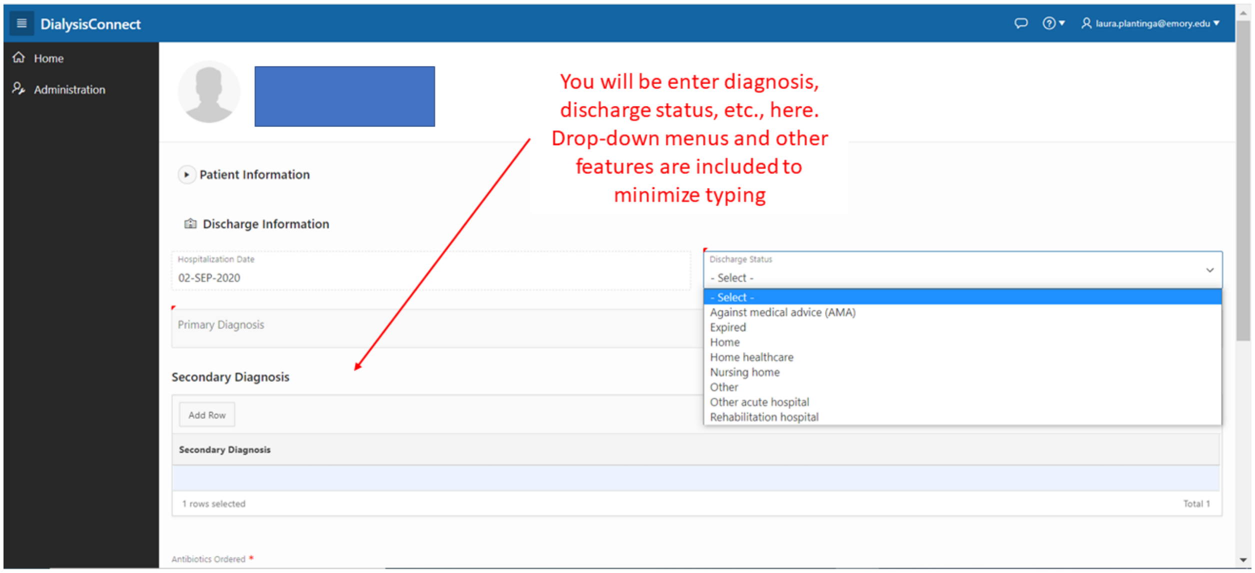

You will need to scroll to complete the discharge information:

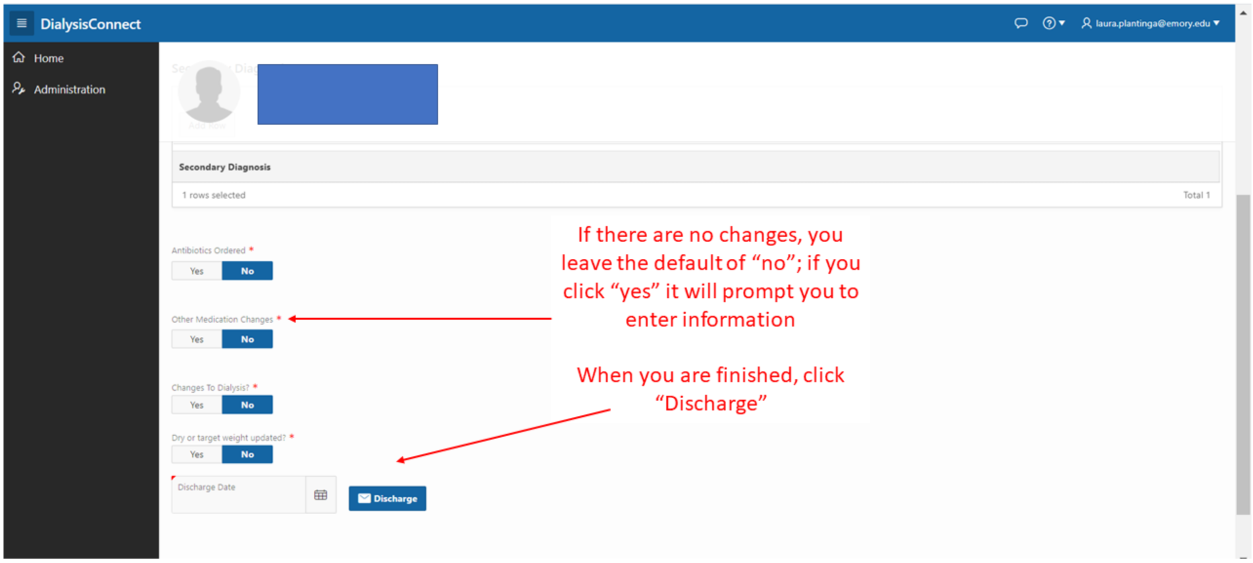

When you click "discharge," a message is instantly sent to the dialysis clinic that a patient has been discharged, with a prompt to log in and review the information. The patient will move to the "Previous Hospitalizations" tab.

Note: if you wish to require a response at discharge, you can click the response requested directly on the discharge page (without sending a separate message):

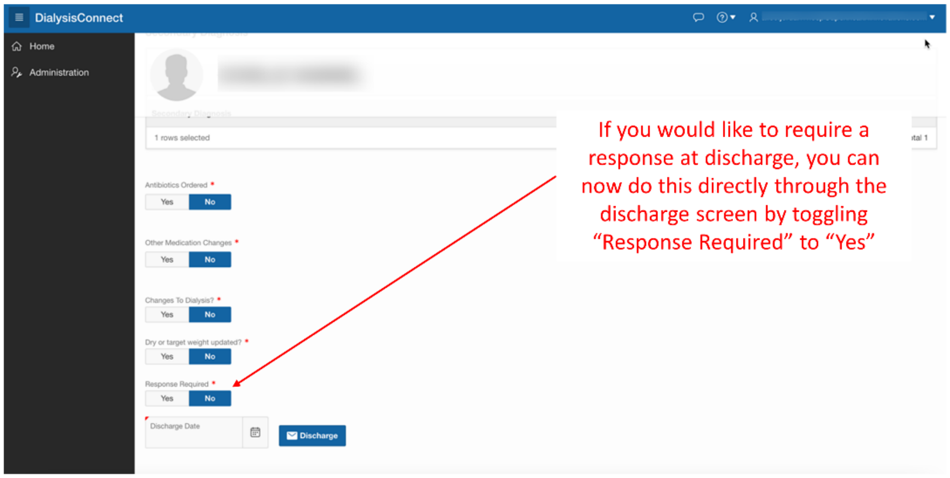

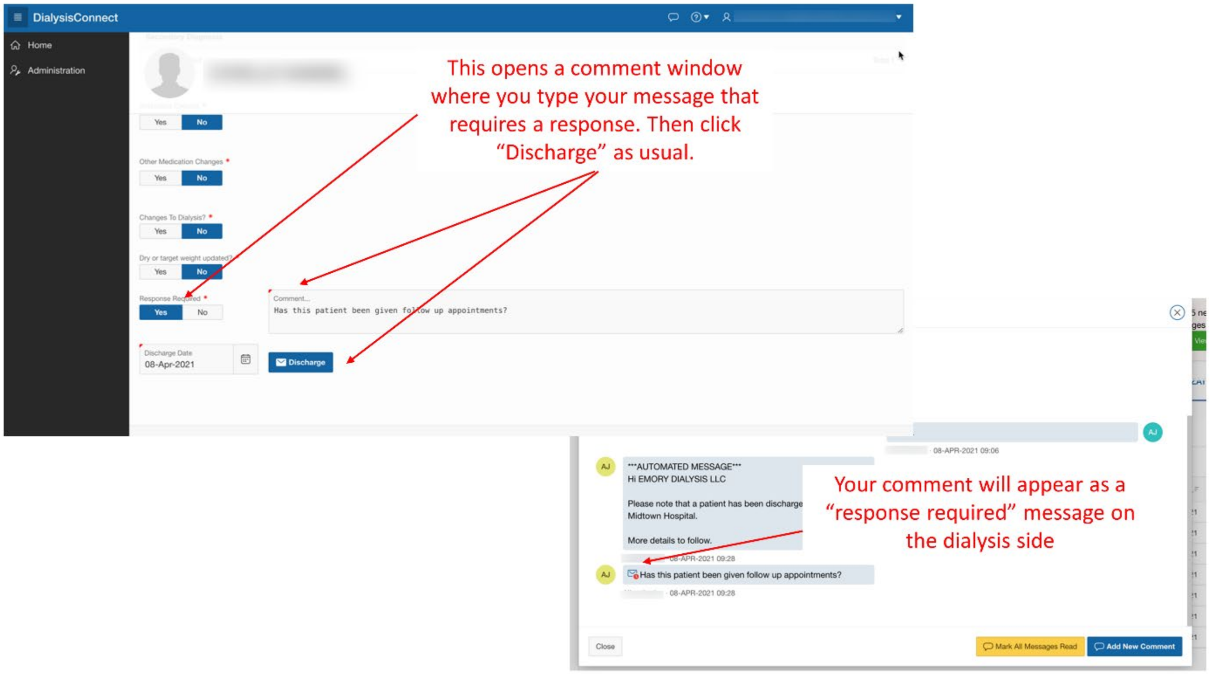

Acknowledgements

We thank all the providers who participated in our focus groups and user testing and provided invaluable feedback to create DialysisConnect. We also thank everyone at Emory Healthcare (Atlanta, GA) and Health Services Management, Inc. (Tifton, GA), who provided not only insight into current processes but also data to support DialysisConnect.

Study Team

Emory University:
Laura Plantinga (Principal Investigator)
Kyle James (Co-Investigator)
Janice Lea (Co-Investigator)
Tahsin Masud (Co-Investigator)
Christopher O'Donnell (Co-Investigator)
Ann Vandenberg (Co-Investigator)
Courtney Hoge (Project Manager)
Christian Park (Research Assistant)

Apex Health Innovations:
Rich Mutell (Chief Executive Officer; Consultant)
John Scott (Chief Technology Officer)
Charlie Bonar (Compliance Representative)
Richard Dacre (Head of Development)
Alex Turnbull (Senior Developer)
Amber Webster (Senior Developer)
Jasper Kirby (Developer)

Johns Hopkins University:
Bernard Jaar (Consultant)

DialysisConnect

EMORY UNIVERSITY SCHOOL OF MEDICINE

apex health innovations

Study Funding

This study is funded by the National Institute of Diabetes and Digestive and Kidney Diseases (NIDDK; R18DK118467). The content of this guide is solely the responsibility of the study team and does not necessarily reflect the views of the National Institutes of Health.

NIH National Institute of Diabetes and Digestive and Kidney Diseases
