# Supplementary material for: A Web-Based, Provider-Driven Mobile App to Enhance Patient Care Coordination Between Dialysis Facilities and Hospitals: Development and Pilot Implementation Study
Source: JMIR Form Res. 2022 Jun 10;6(6):e36052. doi: 10.2196/36052 (PMC9233252; doi:10.2196/36052)
Supplement: Multimedia Appendix 5 [file formative_v6i6e36052_app5.pdf]

A Quick Start Guide for Dialysis Clinic Users

Version 2.0, 4/14/21

Table of Contents

Part 1. About DialysisConnect....3

What is DialysisConnect?....3

How was DialysisConnect developed?....3

Can we propose changes to DialysisConnect?....3

Is DialysisConnect part of the EMR?....3

Who is using DialysisConnect?....3

Where can I find DialysisConnect?....4

How do I access DialysisConnect?....4

Will I be able to use DialysisConnect after the pilot study is over?....5

What browser should I use to view DialysisConnect?....5

Can I use my mobile phone to view DialysisConnect?....5

Can I create an icon on my laptop/computer or smartphone that goes directly to DialysisConnect?....5

Will DialysisConnect "time out" if I am logged in but inactive?....6

How will DialysisConnect let me know when a patient is admitted to or discharged from the hospital, or when there is a request for information to review?....6

Contact us....6

Part 2. How to Use Dialysis Connect....7

Explore the home page....7

What happens when a dialysis patient is admitted....8

Step 1: Notification that a patient has been admitted....8

Step 2: Sending messages and documents....10

Step 3: Notification that a patient has been discharged....17

Acknowledgements....21

Study Team....21

Study Funding....21

Part 1. About DialysisConnect

What is DialysisConnect?

DialysisConnect is a secure, HIPAA-compliant, web-based communications platform to facilitate coordination of care of dialysis patients during and after hospitalizations.

How was DialysisConnect developed?

We have developed DialysisConnect through an iterative process, through the ongoing feedback of our study team (which consists of nephrologists and hospitalists as well as scientists and developers); feedback on an initial proposed system via focus groups of Emory Dialysis and Emory University Hospital Midtown (EUHM) staff involved in care transitions of dialysis patients; and feedback during user testing of the working beta system.

Can we propose changes to DialysisConnect?

Yes. The iterative nature of DialysisConnect development means that we can make changes and enhancements during the pilot phase. It is important to note that some proposed changes may be too intensive to roll out quickly during the pilot phase; however, feedback from users during the pilot phase will be collected and used to develop the next version(s) of DialysisConnect. We will also seek feedback during the pilot phase via brief online provider surveys.

Is DialysisConnect part of the EMR?

No. At this time, DialysisConnect is a stand-alone system that is not integrated with Emory Dialysis' electronic medical record (EMR). *DialysisConnect not a patient care platform (in which you'd be able to send orders, etc.)*. Rather, it is a communications platform where you send information about the patient securely between settings.

However, some information from the EMR will be available: we are currently manually uploading regular file feeds from the Emory Dialysis system with patient information (demographic and clinical information) so that this information is available to hospital providers. However, we hope this pilot study will provide evidence that this system can help improve patient outcomes and support this eventual integration.

Who is using DialysisConnect?

While we eventually aim to roll Dialysis Connect out to a larger group of providers, for this pilot study, only *dialysis providers at Emory Dialysis* and *hospital providers at EUHM* are using DialysisConnect. Thus, for the pilot study period (6 months), the system will only be used for patients who are receiving dialysis at an Emory Dialysis facility (Northside, Candler, Greenbriar, and North Decatur) and are admitted to EUHM.

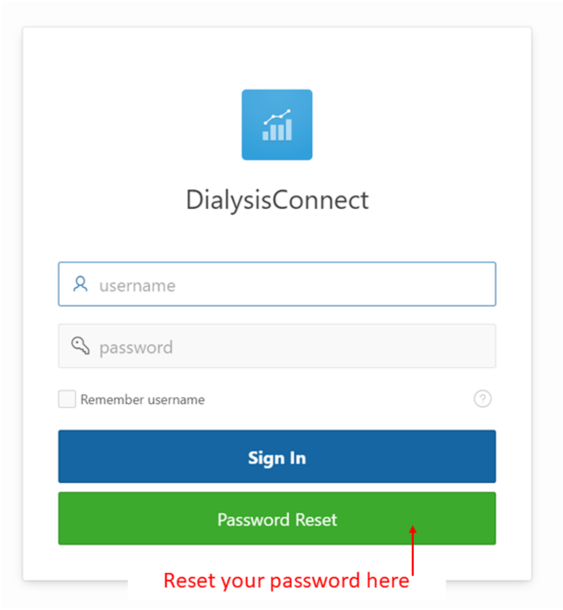

Where can I find DialysisConnect?

DialysisConnect is a web-based application that can be found at the following URL: https://dialysisconnect.com/. This page may be bookmarked. Some browsers also allow webpages to be pinned to the Start menu or added to the Desktop. Please contact us if you would like to do this and need help.

How do I access DialysisConnect?

Once you navigate to the DialysisConnect page, you will enter your username and password to enter the system (left). Your username is your email address. Your password will be assigned when you are added to the system; you can change this by clicking on "Password Reset."

DialysisConnect was built on the same platform as T-REX, the transplant referral exchange system. Upon login, you will have to select DialysisConnect:

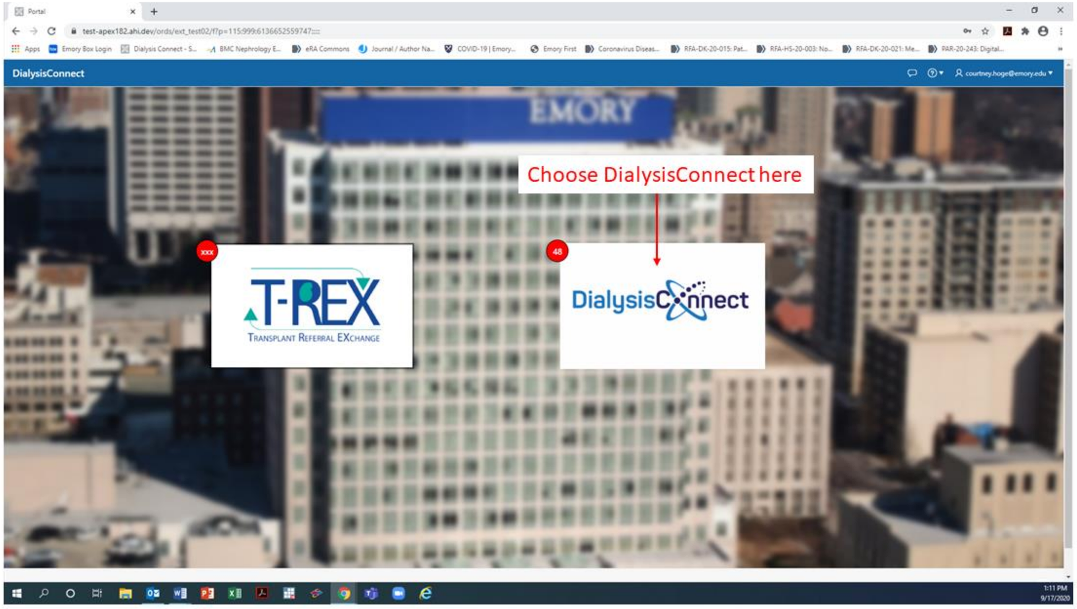

Both DialysisConnect and T-REX are only available to identified users of the respective systems. If you would like to be added as a user, or are having trouble accessing the system, please contact us.

Will I be able to use DialysisConnect after the pilot study is over?

Yes, DialysisConnect will remain available after the pilot study data collection period ends. However, technical support may be limited until or unless follow-up funding is secured.

What browser should I use to view DialysisConnect?

DialysisConnect was developed on Chrome and thus is optimally viewed with this browser. **Please note that DialysisConnect will not function on Internet Explorer 11** (which is no longer supported by Microsoft); you will be re-directed if you attempt to use Internet Explorer 11.

Can I use my mobile phone to view DialysisConnect?

Yes. However, note that pages configured for mobile phones are only available through links in SMS messages. If you log into DialysisConnect from a browser on your phone, the system will be visible and functional, but the pages will not be specifically configured for mobile phone viewing.

Can I create an icon on my laptop/computer or smartphone that goes directly to DialysisConnect?

Yes. You can create a shortcut on your desktop or on your phone’s home screen to the DialysisConnect website, as shown below. If you need help adding an icon on your Android phone, please contact us.

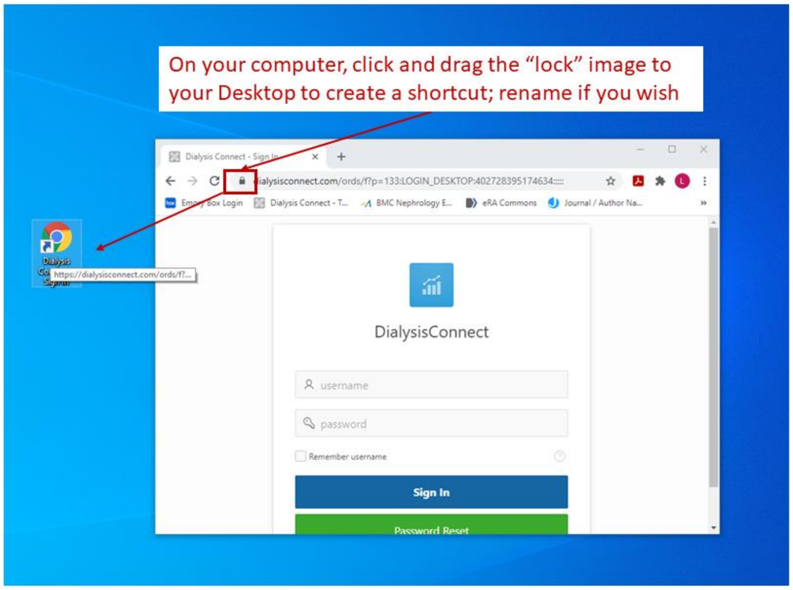

On your iPhone, with the website open in Safari, click “Share” at the bottom of the screen and then select “Add to Home Screen” in the popup window

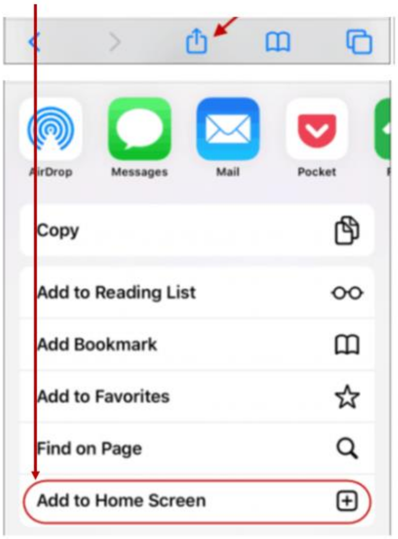

Will DialysisConnect “time out” if I am logged in but inactive?

Yes, to maintain security of the system, DialysisConnect will time out after **20 minutes** if the user is inactive. Information entered that has not already been submitted will be lost. A pop-up warning will let you know.

How will DialysisConnect let me know when a patient is admitted to or discharged from the hospital, or when there is a request for information to review?

You can allow DialysisConnect to send automated email or SMS (or both) messages when the hospital admits or discharges a patient from your facility, or when they send a request for additional information. To add your mobile number, please contact us.

Because Emory Healthcare and University will not permanently “whitelist” these addresses, you may find the emails go to your junk folder. To add these automated emails to your “safe sender list” go to junk e-mail options and add the domain “@dialysisconnect.com”:

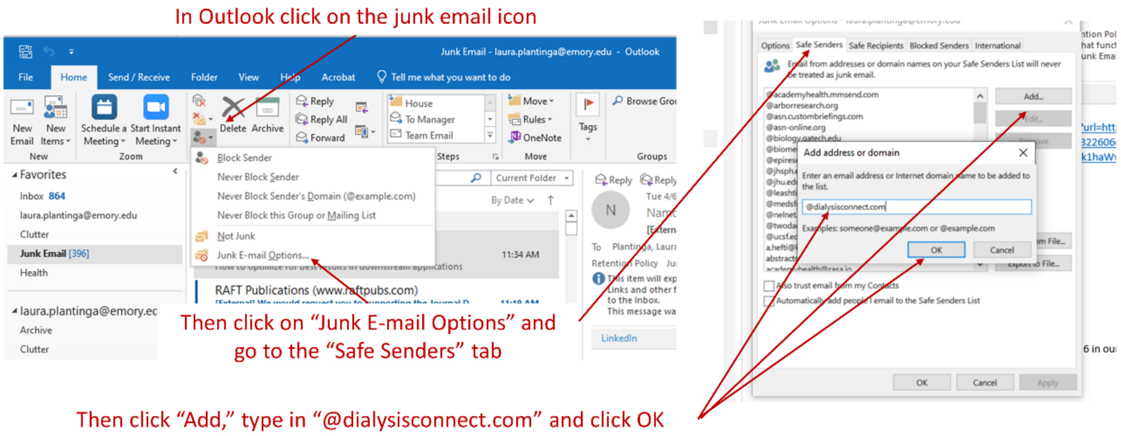

Contact us

If you have questions about the DialysisConnect pilot study or how to use the system, or you would like to request individual training:

Laura Plantinga, Principal Investigator, laura.plantinga@emory.edu
Courtney Hoge, Project Manager, courtney.hoge@emory.edu

If you have technical questions about DialysisConnect system:

dialysisconnect@apexhealthinnovations.com

Part 2. How to Use Dialysis Connect

Explore the home page

Once you log in, you will see the home page.

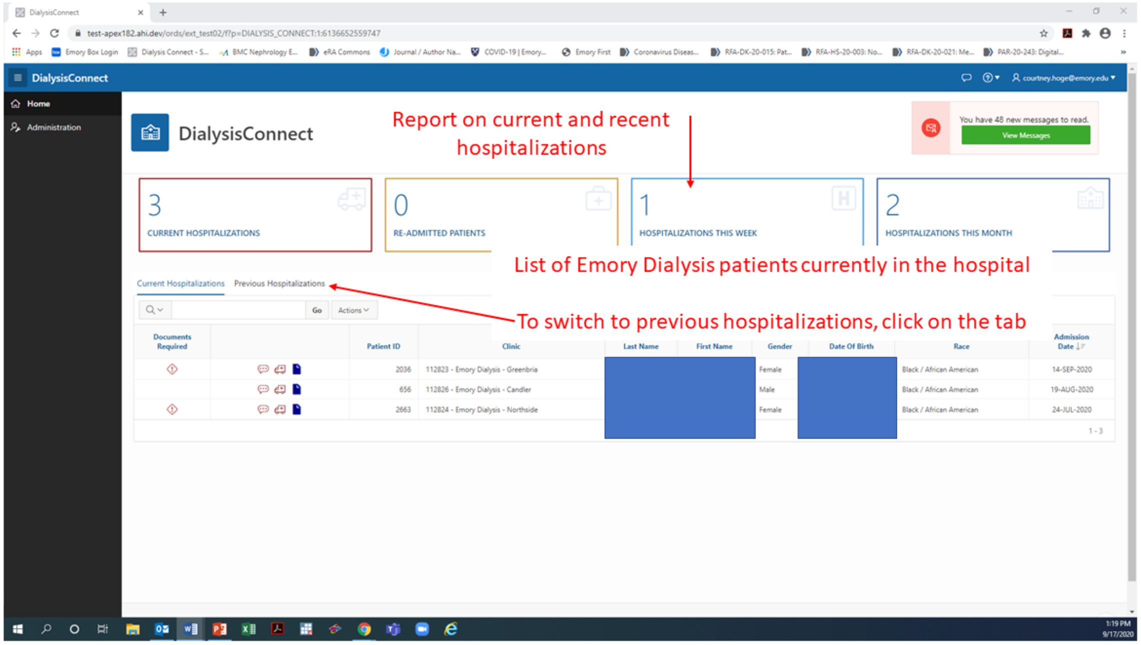

Here you will see an overview report showing the number of Emory Dialysis Patients currently at EUHM, the number re-admitted within 30 days, and the numbers of hospitalizations in the current week and month. You will also see a list of Emory Dialysis patients currently in the hospital (including their clinic and some demographic information, along with their admission dates). Previous hospitalizations can be seen by clicking the “Previous Hospitalizations” tab.

From the home page, messages, hospitalization information, documents received and discharge information (previous hospitalizations only) can be accessed through the text, ambulance, document, and doctor icons, respectively. These will be described in more detail under tasks.

What happens when a dialysis patient is admitted

Step 1: Notification that a patient has been admitted

When hospital staff admit a patient at EUHM and identify a patient as an Emory Dialysis patient, they enter reason(s) for hospitalization and an automated message will be generated. The message is sent via e-mail or SMS text message, or both, depending on your preferences:

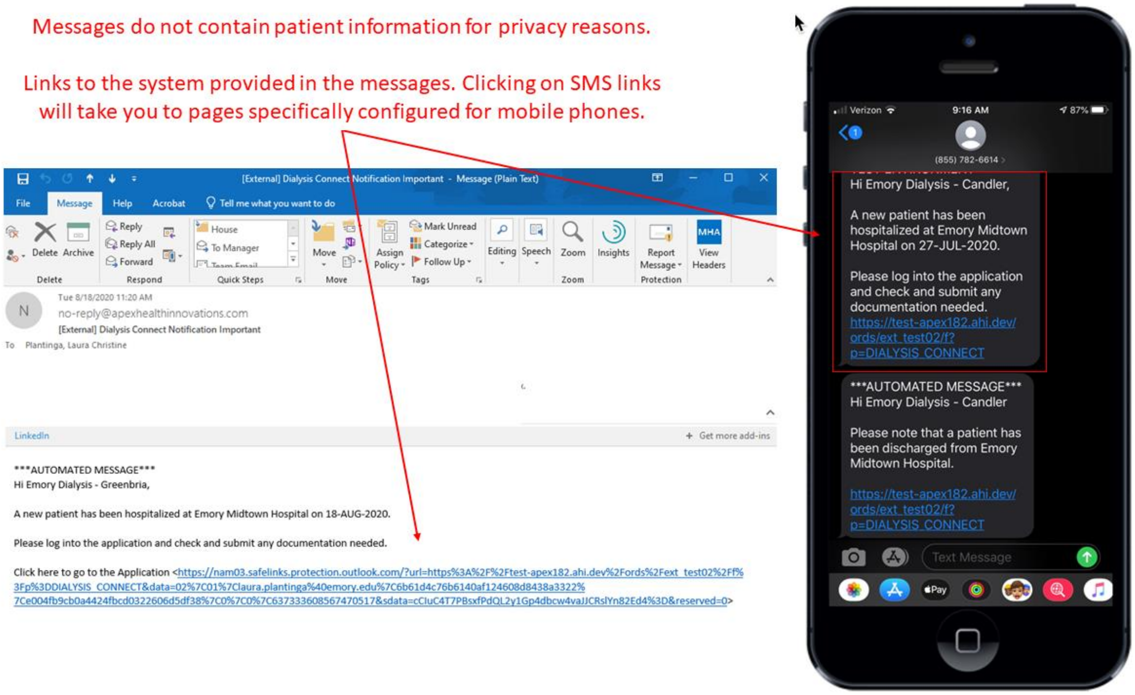

Links from the message can be used to view the information immediately, and/or you can log into the system later to review the information.

Remember if you are on your mobile phone and do not use the link provided in the message (right), you will go to a page that is not configured for the mobile phone.

Information about the hospitalization can be accessed at any time from the “ambulance” icon from the home page:

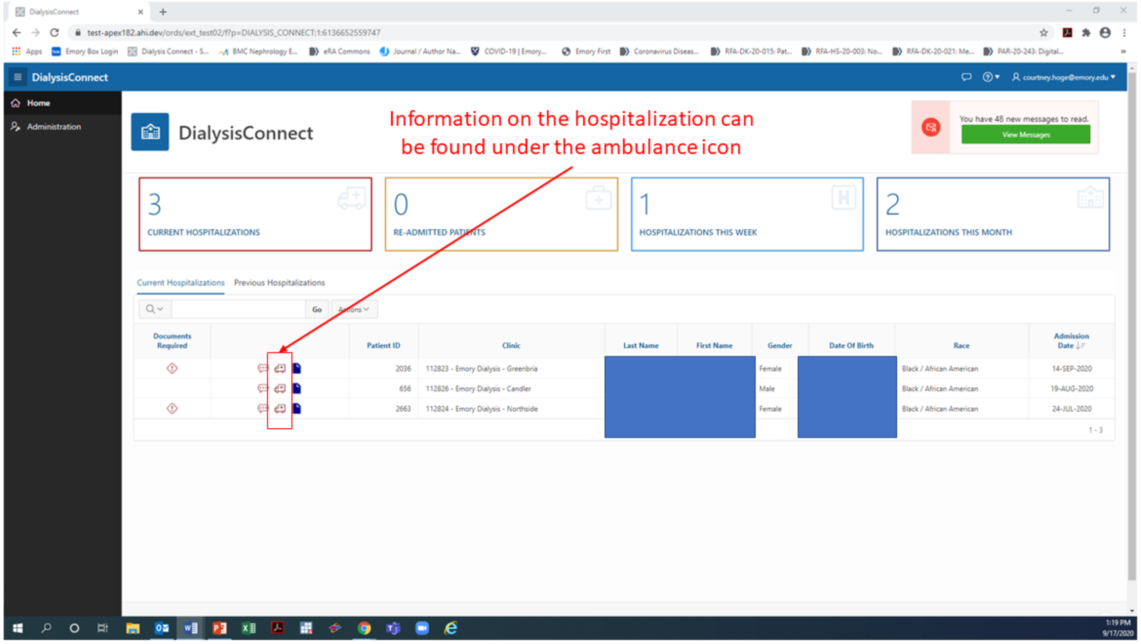

In the popup window, you will first see the reasons for admission as noted by the hospital:

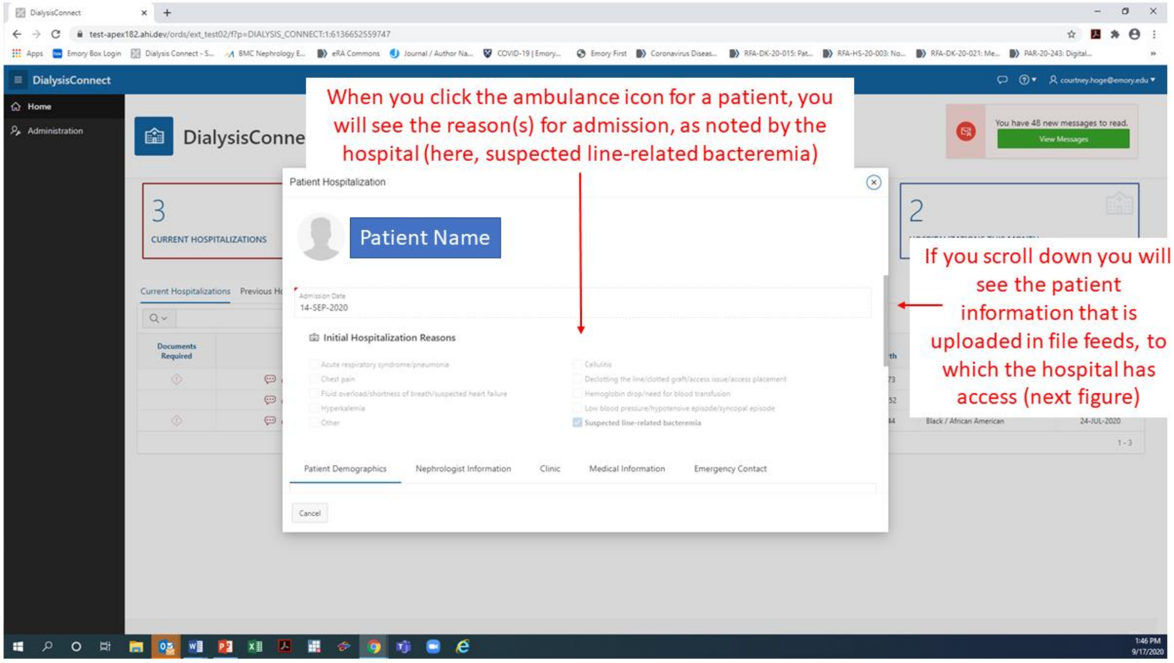

Then if you scroll down, you will see the information about the patient that has been automatically uploaded (and to which the hospital has access at admission):

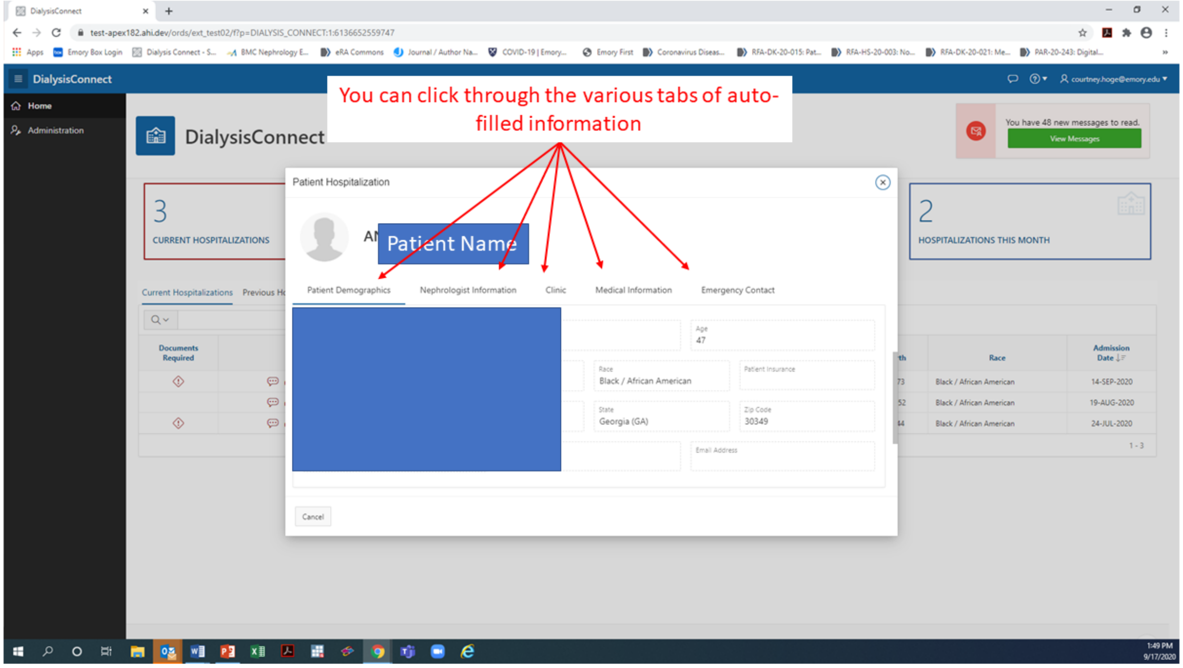

Step 2: Sending messages and documents

While the patient is in the hospital, there may be no DialysisConnect tasks until discharge. However, if you would like more information from the hospital about a patient, you can send a message at any time. Messages can be accessed from the "text bubble" icons or the message box:

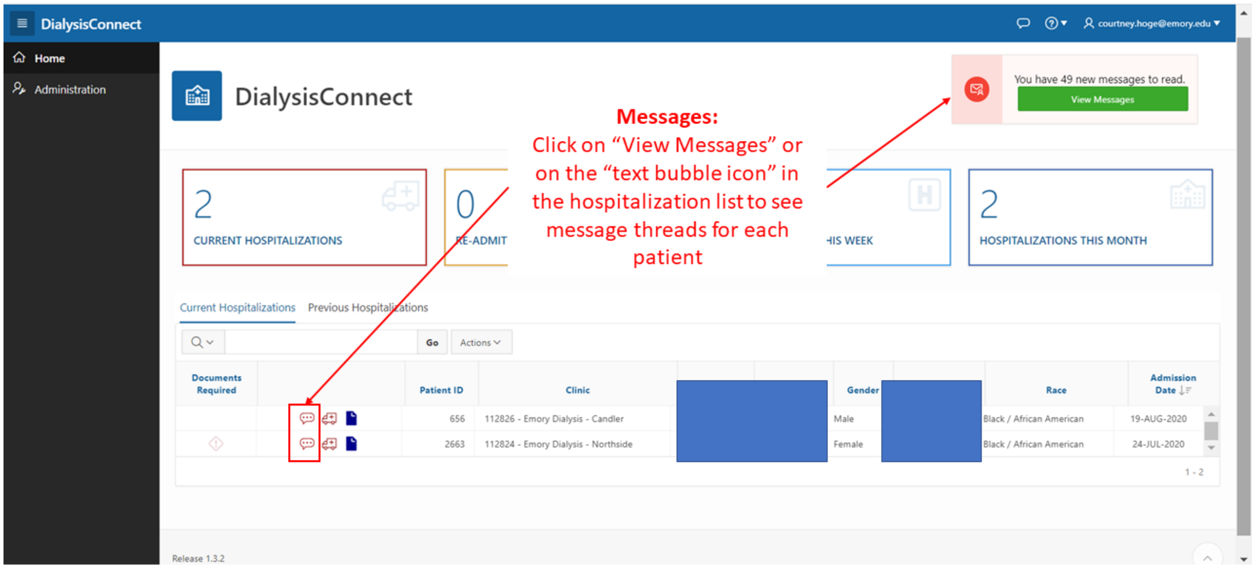

Messages will pop up and will look like a text message exchange:

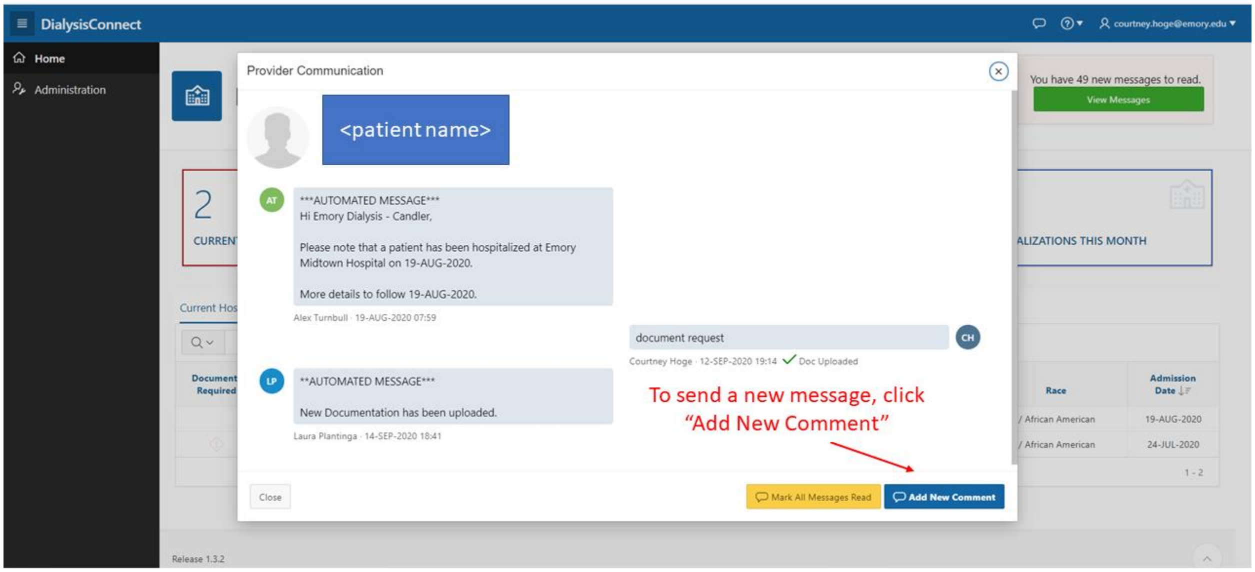

You can click “Add New Comment” to create a new message. If you are requesting a document, you can indicate that before clicking “Create”:

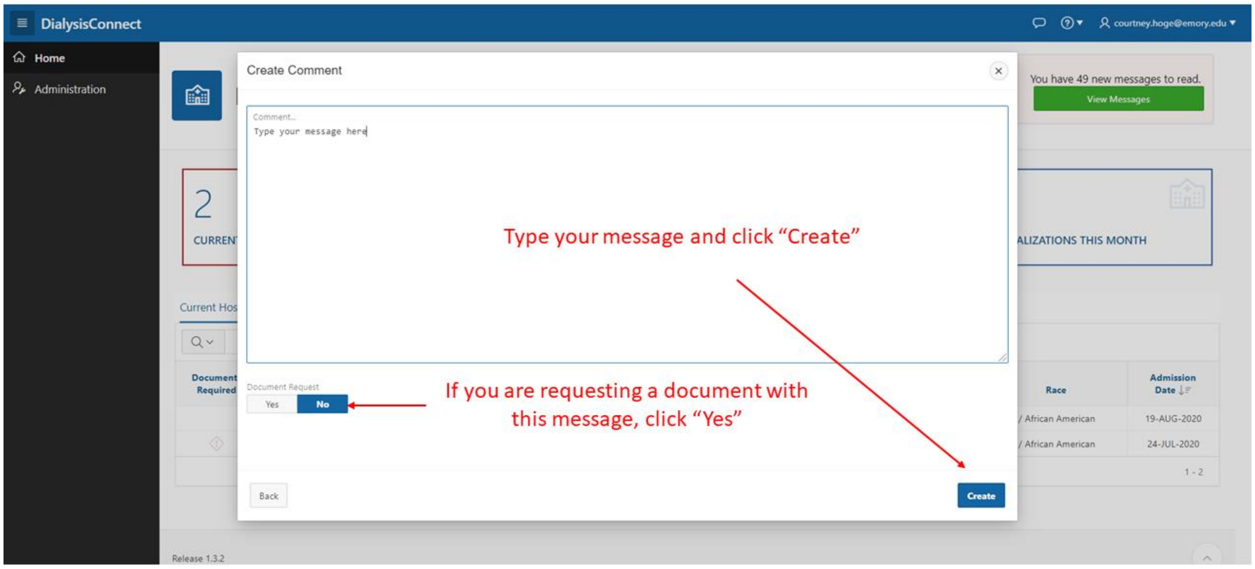

If you want to let the hospital know their messages were received, mark the messages as “read.” If you like to know whether your message to the hospital was “read,” look for the eye icon next to the message:

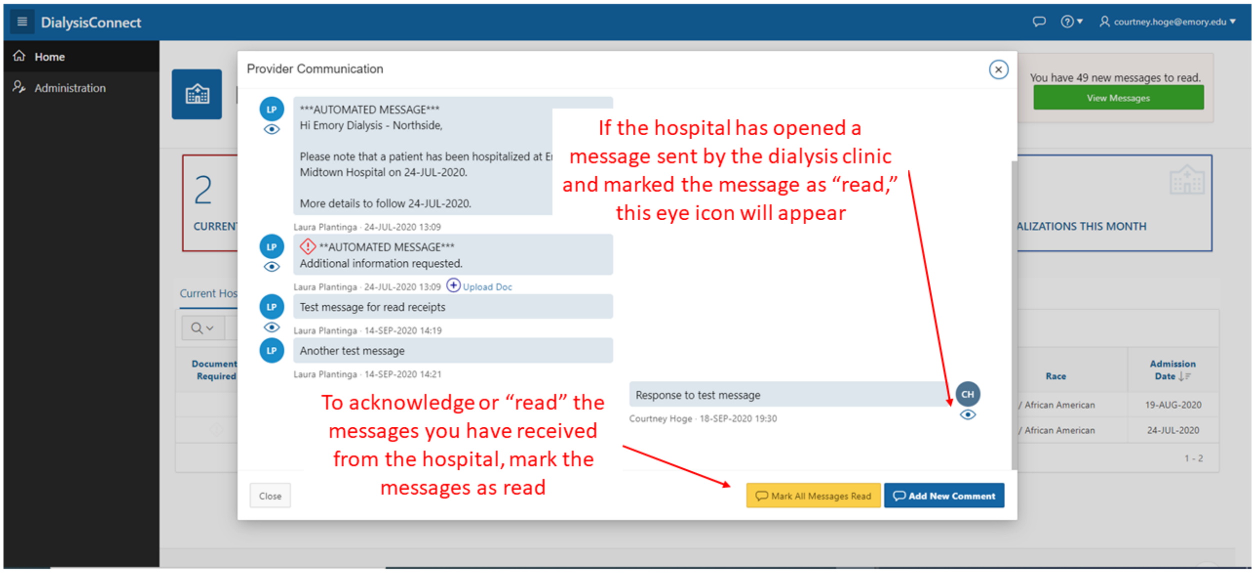

You may request documents from the hospital, or you may get a request for documents from the hospital. Documents can be accessed at any point from the home page:

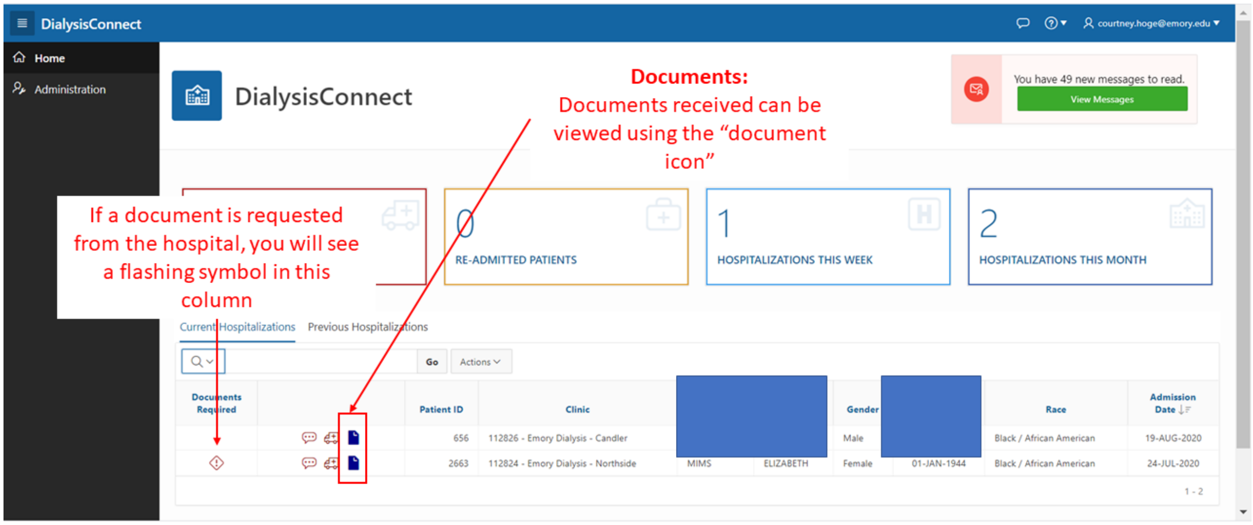

A flashing symbol appears if there is an outstanding document request.

To send a document to the hospital, you may “Upload Document” directly from the message:

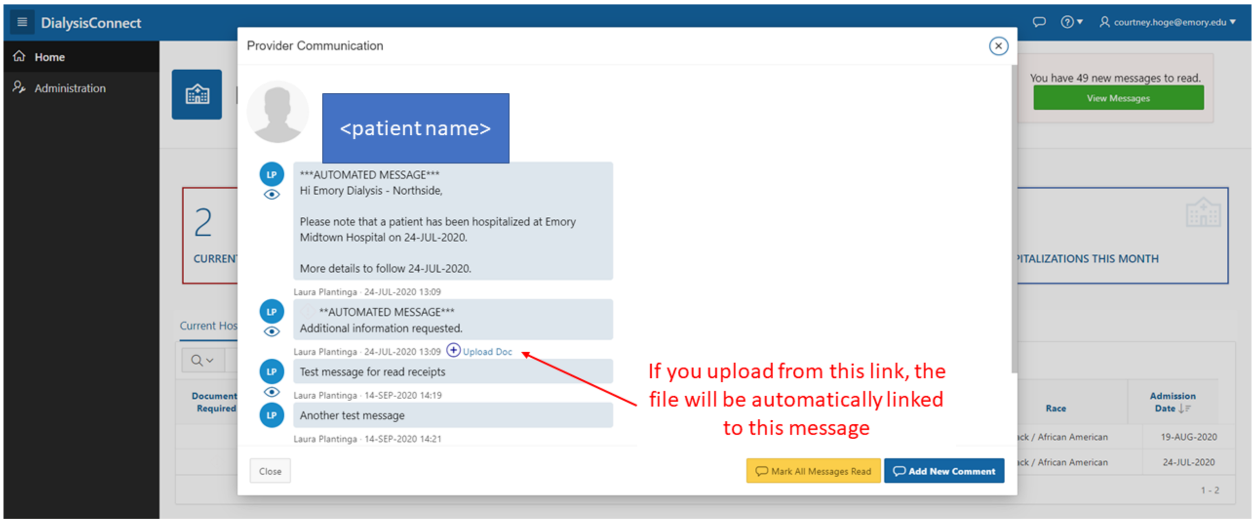

Or, you may upload on the documents page and link the document to a request:

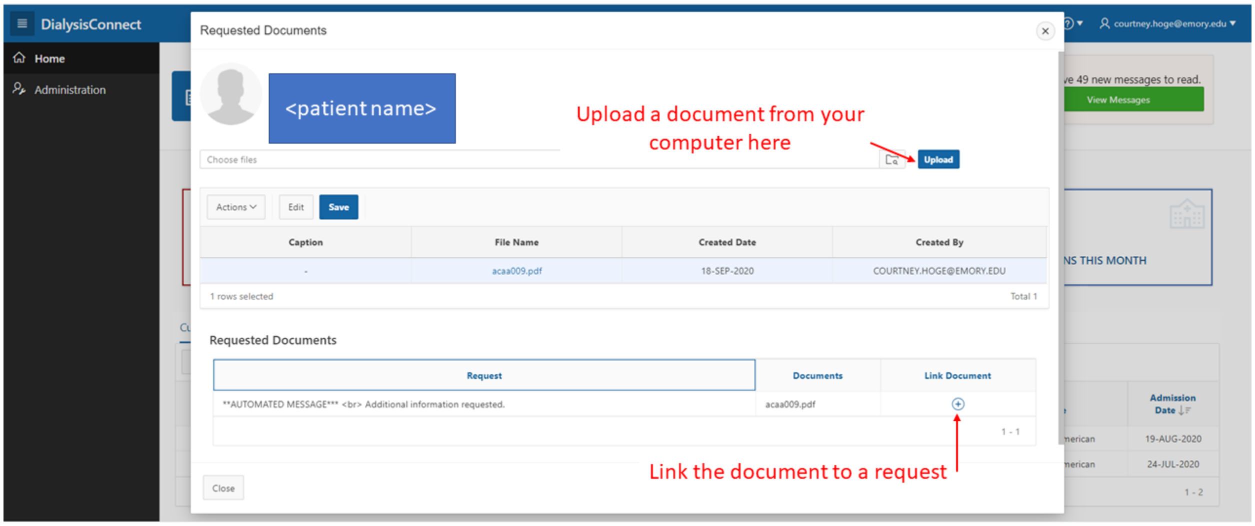

You may wish to request that the hospital confirm that information in a message is being acted upon; for this you may wish to add a “Response Required” tag to your message:

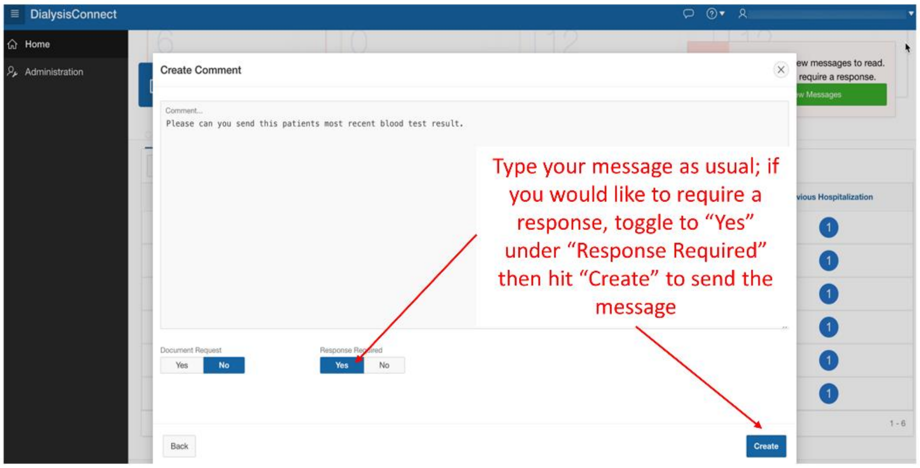

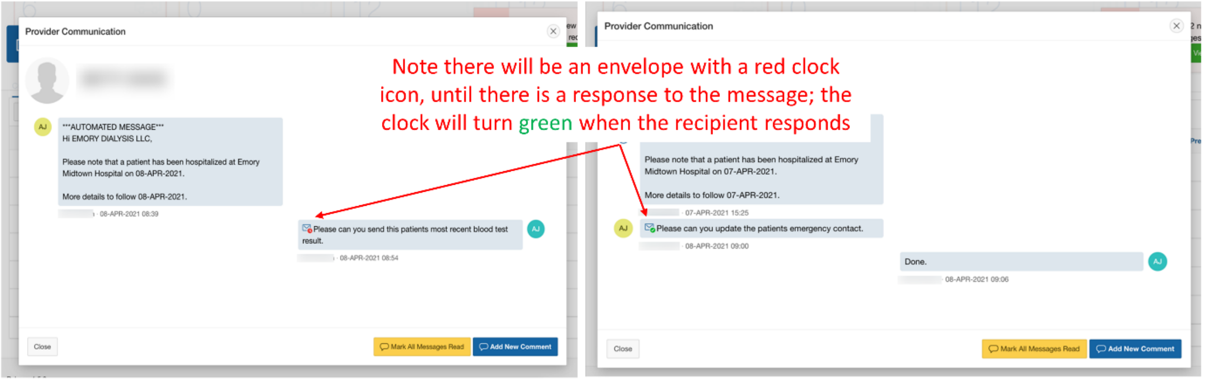

The hospital may also send a "Response Required" message to you. These messages will appear on your home page:

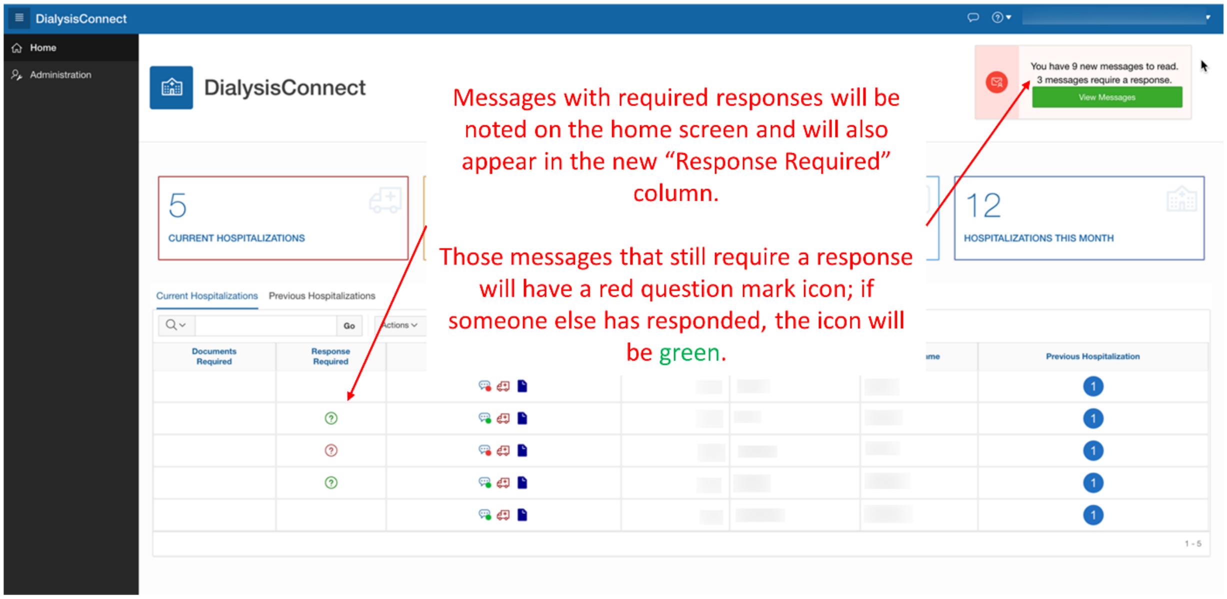

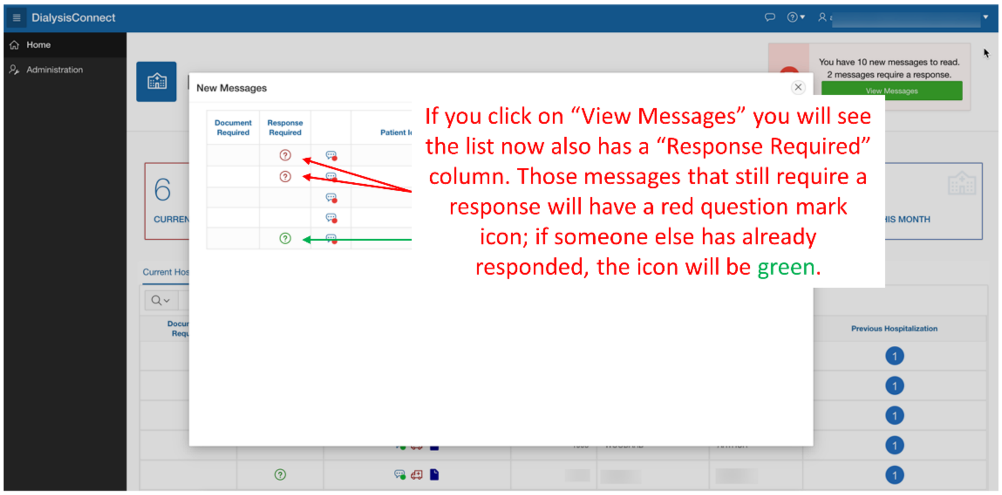

To respond to a message with a “Response Required”:

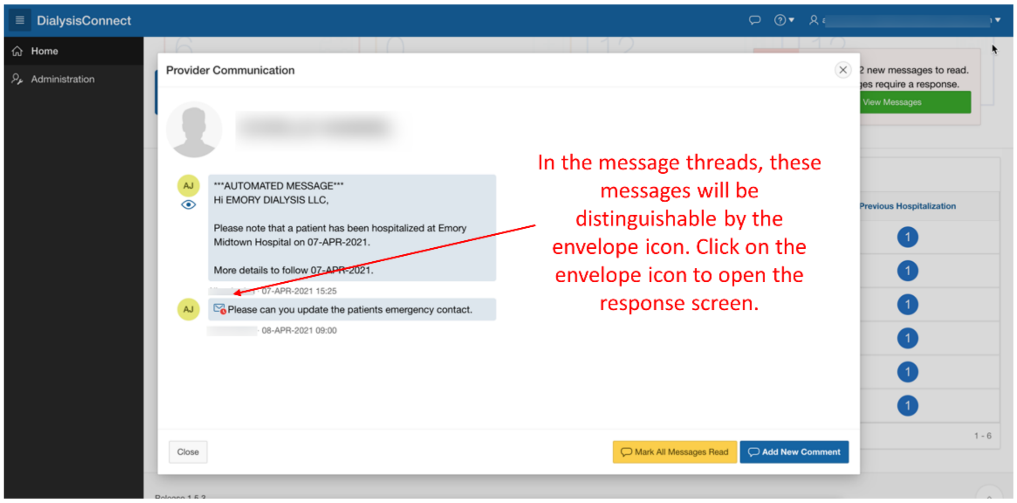

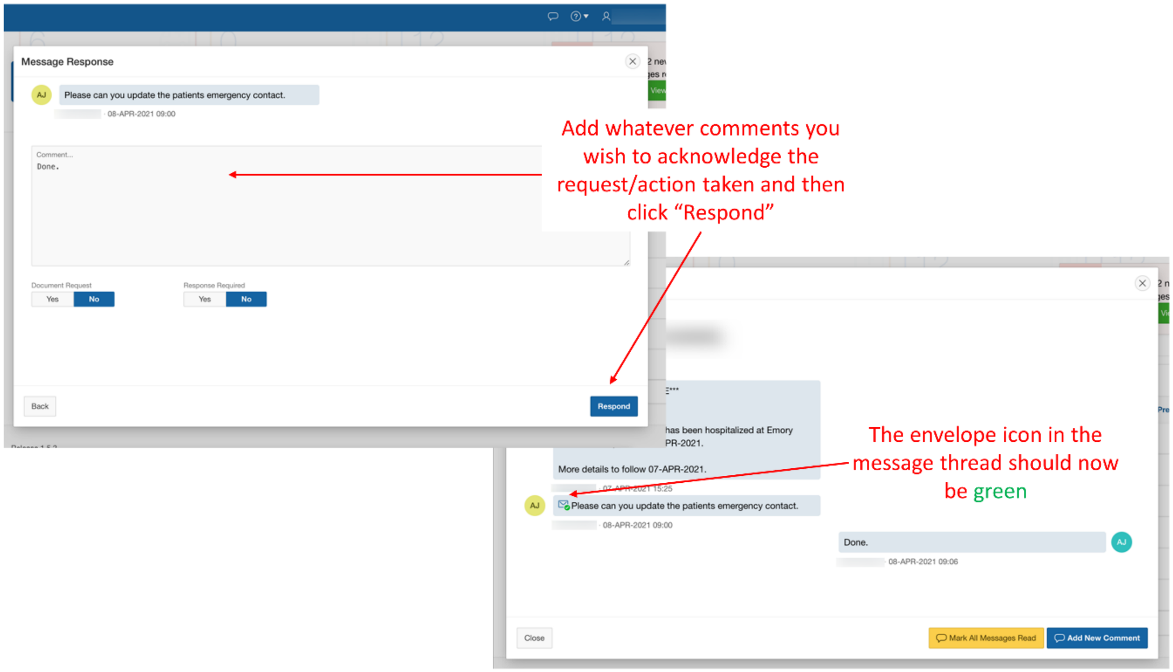

Step 3: Notification that a patient has been discharged

When the patient is discharged, you will receive a message from the hospital to inform you of the discharge:

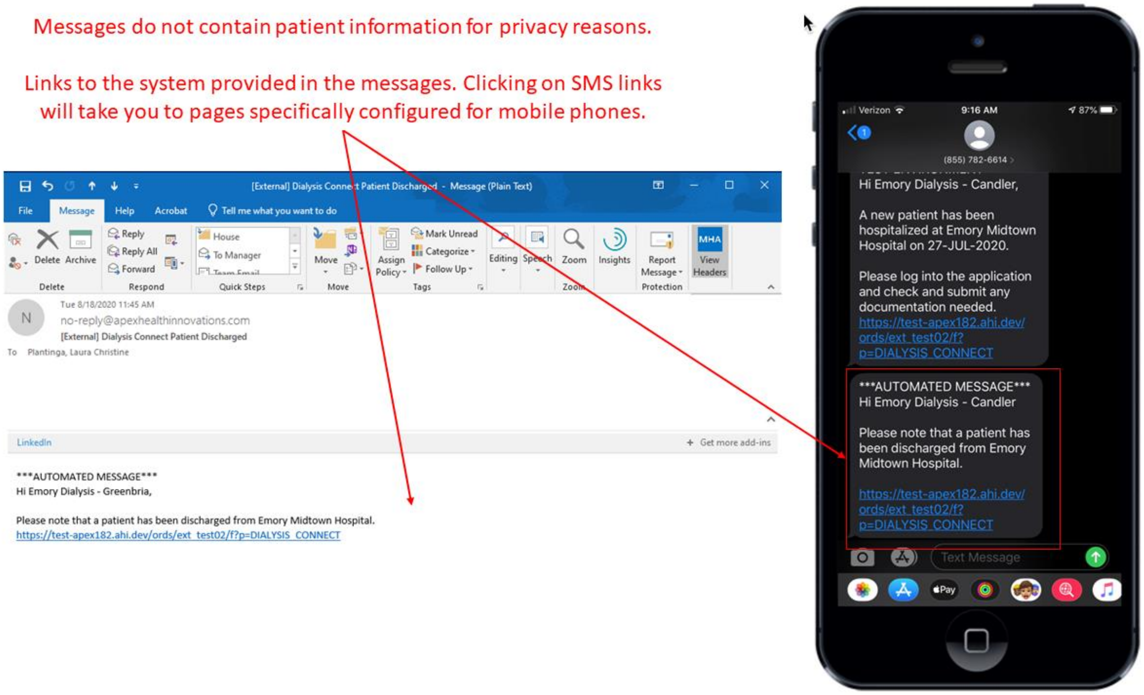

The hospital is being asked to enter some brief, critical information about the hospitalization to assist with the care transition. You will receive an official discharge summary as you are already receiving/accessing it, but this notification should be sent immediately upon discharge.

To see the discharge information, you will have to change to previous hospitalizations:

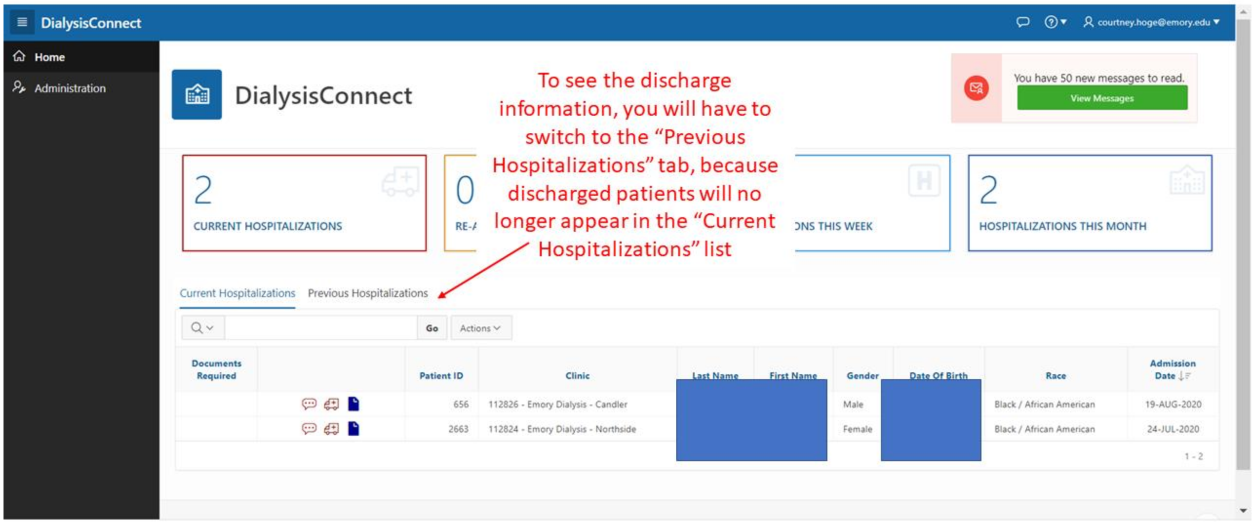
DialysisConnect
courtney.hoge@emory.edu
Home
Administration
DialysisConnect
To see the discharge information, you will have to switch to the “Previous Hospitalizations” tab, because discharged patients will no longer appear in the “Current Hospitalizations” list
You have 50 new messages to read.
View Messages
2
CURRENT HOSPITALIZATIONS
0
RE-A
ONS THIS WEEK
2
HOSPITALIZATIONS THIS MONTH
Current Hospitalizations
Previous Hospitalizations
Go
Actions
Documents Required
Patient ID
Clinic
Last Name
First Name
Gender
Date Of Birth
Race
Admission Date
656
112826 - Emory Dialysis - Candler
Male
Black / African American
19-AUG-2020
2663
112824 - Emory Dialysis - Northside
Female
Black / African American
24-JUL-2020
1 - 2

And then click the “doctor” icon:

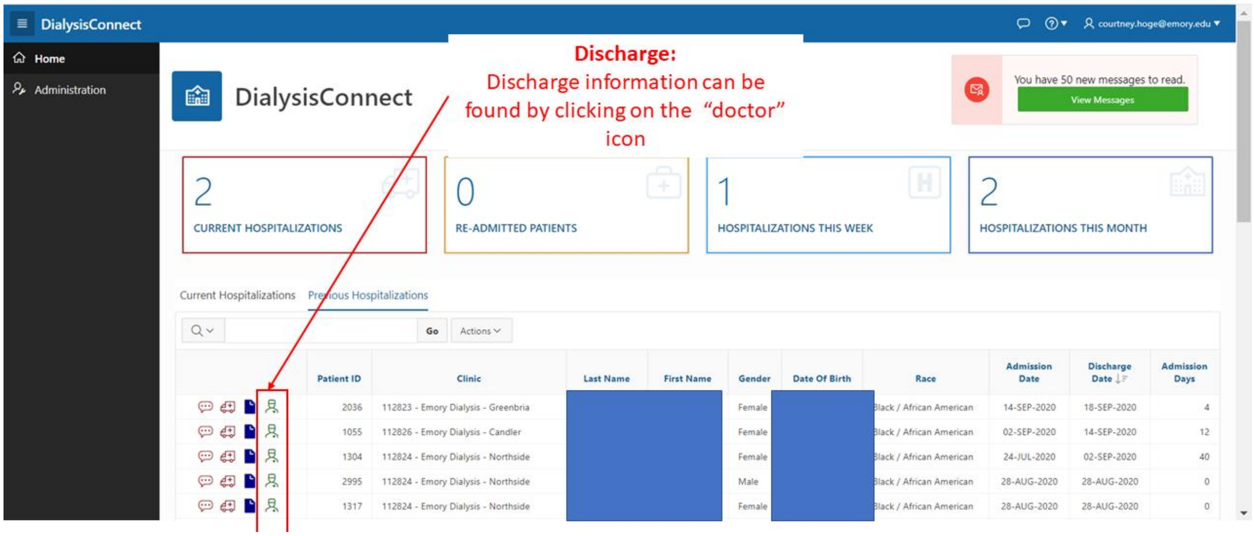

A new window with discharge information will open:

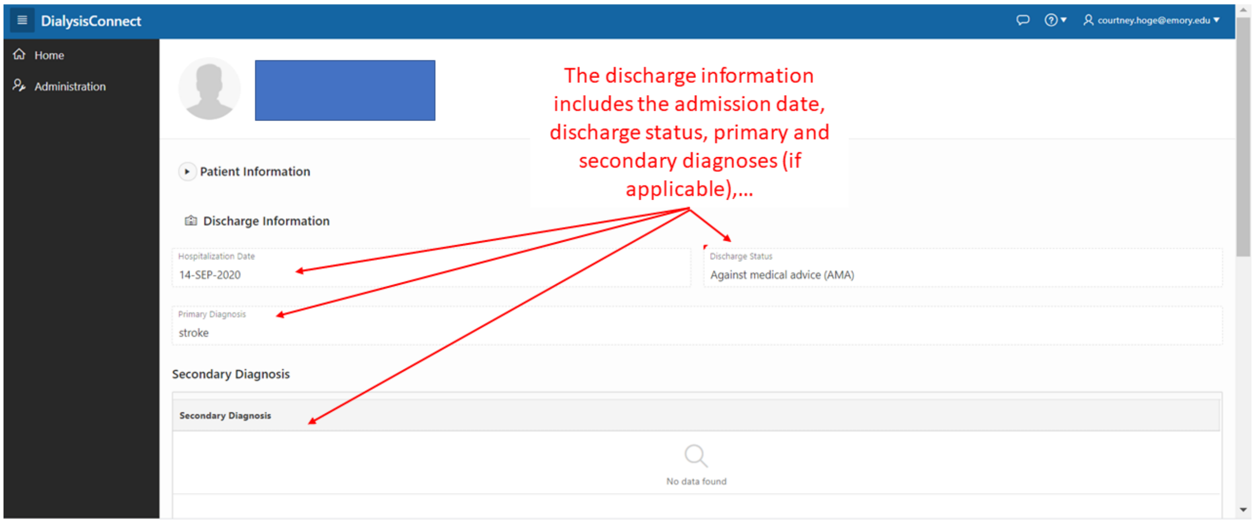

You can scroll through this window for more information:

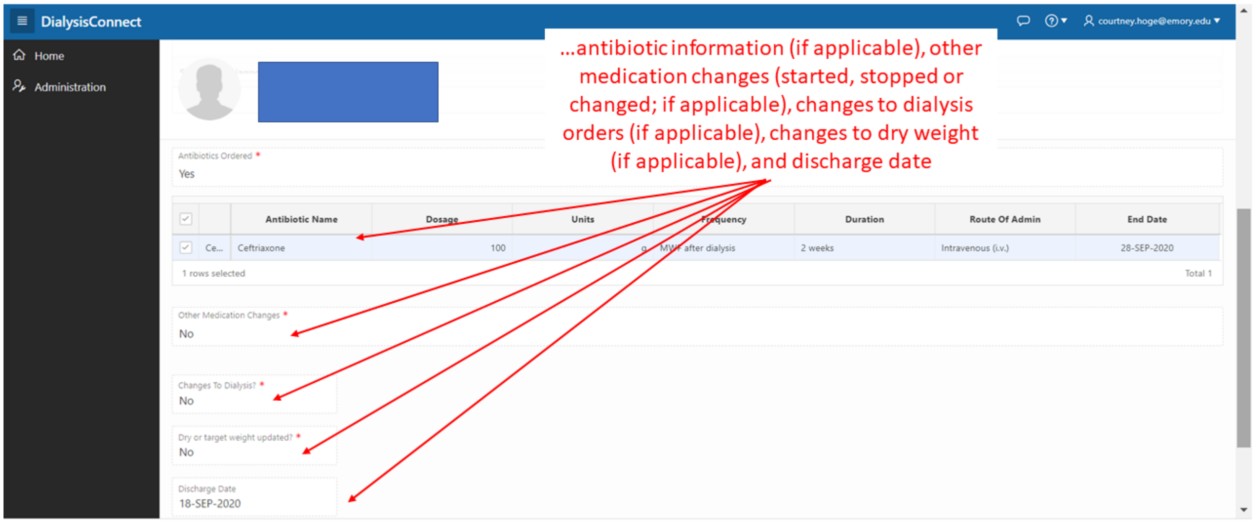

If the hospital has requested a response with their discharge:

Acknowledgements

We thank all the providers who participated in our focus groups and user testing and provided invaluable feedback to create DialysisConnect. We also thank everyone at Emory Healthcare (Atlanta, GA) and Health Services Management, Inc. (Tifton, GA), who provided not only insight into current processes but also data to support DialysisConnect.

Study Team

Emory University:
Laura Plantinga (Principal Investigator)
Kyle James (Co-Investigator)
Janice Lea (Co-Investigator)
Tahsin Masud (Co-Investigator)
Christopher O'Donnell (Co-Investigator)
Ann Vandenberg (Co-Investigator)
Courtney Hoge (Project Manager)
Christian Park (Research Assistant)

Apex Health Innovations:
Rich Mutell (Chief Executive Officer; Consultant)
John Scott (Chief Technology Officer)
Charlie Bonar (Compliance Representative)
Richard Dacre (Head of Development)
Alex Turnbull (Senior Developer)
Amber Webster (Senior Developer)
Jasper Kirby (Developer)

Johns Hopkins University:
Bernard Jaar (Consultant)

DialysisConnect

EMORY UNIVERSITY SCHOOL OF MEDICINE

apex health innovations

Study Funding

This study is funded by the National Institute of Diabetes and Digestive and Kidney Diseases (NIDDK; R18DK118467). The content of this guide is solely the responsibility of the study team and does not necessarily reflect the views of the National Institutes of Health.

NIH National Institute of Diabetes and Digestive and Kidney Diseases
