# Supplementary material for: A Web-Based, Provider-Driven Mobile App to Enhance Patient Care Coordination Between Dialysis Facilities and Hospitals: Development and Pilot Implementation Study
Source: JMIR Form Res. 2022 Jun 10;6(6):e36052. doi: 10.2196/36052 (PMC9233252; doi:10.2196/36052)
Supplement: Multimedia Appendix 6 [file formative_v6i6e36052_app6.docx]

**Provider survey for DialysisConnect**

**You are being asked to complete this survey because you have been identified as a user of DialysisConnect. We are interested in your thoughts about DialysisConnect *regardless of whether you have used the system.***

**1. Have you heard of DialysisConnect?** Yes (go to #2) No (skip to #8)

**2. (if yes to #1) How/when did you hear about DialysisConnect? (check all that apply)**

Seminars/meetings

Participation in a focus groups

Participation in individual user testing

Attendance at a group training session

Attendance at an individual training session

E-mails from the research team

Flyers in my workspace

Colleague recommendation

Other (describe: _______________________)

**3. (if yes to #1) Have you tried to access or use DialysisConnect?** Yes No

**4. (If yes to #3) Have you had any trouble accessing or using the system?** Yes No

**5. (If yes to #4) Have you reached out to the team about technical issues?**  Yes No

**6. (If yes to #5) Were your issues resolved?** Yes No

**7. (if yes to #1) Have you received emails or other communications about system updates since the initial rollout in October 2020?** Yes No

**8. (If no to #1, show this text)** **DialysisConnect is a secure, HIPAA-compliant, web-based platform to facilitate coordination of care of dialysis patients during and after hospitalizations. In this system, you can:**

**Hospital:**

- **Inform the Emory Dialysis clinics that their patient has been hospitalized**
- **Consult current patient information that is uploaded monthly from the Emory Dialysis electronic health record (*e.g*., patient’s nephrologist, dialysis schedule, comorbidities, labs, medications)**
- **Securely exchange messages and relevant documents with the dialysis clinic**
- **Add and update time-sensitive patient discharge information (including antibiotic orders, medication changes, dry weight and dialysis changes) for the dialysis clinic**

**Dialysis:**

- **Check hospitalization status for patients admitted to Emory University Hospital Midtown (EUHM)**
- **Securely exchange messages and relevant documents with EUHM, with the option to receive external messages at admission and discharge**
- **Check a patient’s discharge information**

**The web-based platform can be found at www.dialysisconnect.com. You can access the system by resetting your password using your email address (which is your username). A quick-start guide is attached here.**

**8. We are interested in your thoughts about DialysisConnect, regardless of whether you have used it.**

**Please tell us how much you agree with the following statements:**

Not at all To some extent To a great extent)

1. I can see how DialysisConnect differs from usual ways of communicating with dialysis facilities/hospitals
2. I understand how DialysisConnect could improve transitions of care for dialysis patients
3. Users of the system in this organization have a shared understanding of the purpose of DialysisConnect
4. I can see the potential value of DialysisConnect for my work with dialysis patients
5. There are key people at my institution who drive DialysisConnect forward and get others involved
6. I believe that participating in DialysisConnect is a legitimate part of my role in caring for dialysis patients
7. I am open to working with colleagues to optimize our use of DialysisConnect for patient care
8. I support DialysisConnect
9. I can easily integrate DialysisConnect into my existing work
10. DialysisConnect disrupts working relationships
11. I have confidence in other people’s ability to use DialysisConnect
12. Work is assigned to those with skills appropriate to DialysisConnect
13. Sufficient training is provided to enable staff to implement DialysisConnect
14. Sufficient resources are available to support DialysisConnect
15. The staff here agree that DialysisConnect is worthwhile
16. Management adequately supports DialysisConnect
17. I value the effects that DialysisConnect has had on my work
18. Feedback about DialysisConnect can be used to improve it in the future
19. I can easily modify how I work with DialysisConnect

**9. In your opinion, what are the potential motivator(s) to using DialysisConnect? (check all that apply)**

DialysisConnect can reduce time and energy spent gathering information from the dialysis clinic/hospital

It can help me provide more informed care or support for dialysis patients

It can help me provide more timely care or support for dialysis patients

It can improve communication with providers at my institution about dialysis patient care coordination

It can improve communication with providers outside my institution about dialysis patient care coordination

It can help me build better relationships with dialysis patients due to improved perceptions of patient-centeredness of care

It can improve dialysis patients’ clinical outcomes

It can reduce overall utilization and costs of care among dialysis patients

Other (describe ______________)

**9a. What would you say is the *biggest* potential motivator to using DialysisConnect? (choose one)**

DialysisConnect can reduce time and energy spent gathering information from the dialysis clinic/hospital

It can help me provide more informed care or support for dialysis patients

It can help me provide more timely care or support for dialysis patients

It can improve communication with providers at my institution about dialysis patient care coordination

It can improve communication with providers outside my institution about dialysis patient care coordination

It can help me build better relationships with dialysis patients due to improved perceptions of patient-centeredness of care

It can improve dialysis patients’ clinical outcomes

It can reduce overall utilization and costs of care among dialysis patients

Other (describe ______________)

**10. In your opinion, what are the potential barrier(s) to using DialysisConnect? (check all that apply)**

I don’t have time to learn a new system

I don’t have time to use the system in addition to my usual clinical duties

I don’t have a computer/tablet/phone available at the bedside

It seems like DialysisConnect duplicates my work effort

I haven’t been able to access the site

I haven’t been able to navigate the site

Not enough training or supporting documentation was provided to help me learn the system

There does not seem to be a DialysisConnect “champion” at EUHM/Emory Dialysis

Using DialysisConnect is not a priority for my clinical colleagues

Using DialysisConnect is not a priority for clinical leadership

Other (describe_______________)

**10a. What would you say is the *biggest* potential barrier to using DialysisConnect? (choose one)**

I don’t have time to learn a new system

I don’t have time to use the system in addition to my usual clinical duties

I don’t have a computer/tablet/phone available at the bedside

It seems like DialysisConnect duplicates my work effort

I haven’t been able to access the site

I haven’t been able to navigate the site

Not enough training or supporting documentation was provided to help me learn the system

There does not seem to be a DialysisConnect “champion” at EUHM/Emory Dialysis

Using DialysisConnect is not a priority for my clinical colleagues

Using DialysisConnect is not a priority for clinical leadership

Other (describe_______________)

**11. What is your role at the hospital/dialysis center?**

Physician APP Nurse Social Worker Other

**12. How long have you been working at EUHM/Emory Dialysis?**

0-6 months 6-12 months >1 year

**13. (if yes to #3) Do you intend to continue using DialysisConnect?** Yes No

**(if no to #1 or no to #3) Do you intend to start using DialysisConnect?** Yes No

**14. If you have suggestions for improvement of DialysisConnect, please describe them here.**

**15. Is there anything else you’d like us to know about the system?**
